# Transferability of Transcriptome-Based Gene-Dependency Models from Two-Dimensional Cancer Models to Patient-Derived Organoids: Blinded External Validation

**DOI:** 10.3390/biology15191782

**Published:** 2026-10-08

**Authors:** Yelda Fırat, Yılmaz Kılıçaslan

**Affiliations:** 1 Department of Computer Engineering, Artvin Çoruh University, Artvin 08000, Türkiye; yeldafirat@artvin.edu.tr; 2 Department of Artificial Intelligence and Data Engineering, Istanbul Atlas University, Istanbul 34403, Türkiye

**Keywords:** gene dependency, patient-derived organoids, functional genomics, transcriptomics, CRISPR screening, Elastic Net, external validation, cross-model transferability

## Abstract

Transferability of transcriptome-based gene-dependency models from two-dimensional cancer models to organoids remains uncertain. Expression-only Elastic Net models for 20 preselected genes were selected using patient-grouped nested cross-validation in 681 models and evaluated in a locked 171-model holdout. Historical target selection used all 852 models, making holdout performance conditional internal confirmation rather than an unbiased generalization estimate. After refitting on all 852 models, frozen models underwent exploratory evaluation in 94 Broad organoids and blinded validation in 148 Sanger organoids from 144 donors. Predictions were sealed before Sanger outcome access. Primary target-wise Spearman correlations, ranking organoids per gene, averaged 0.4094, 0.1518, and 0.1015 in holdout, Broad, and Sanger, respectively. Sanger association exceeded the within-lineage permutation null (*p* = 0.00040), but improvement over lineage-only prediction remained uncertain (difference, 0.0462; 95% confidence interval, −0.0065–0.0986). *CDK6*, *MDM4*, and *CCNE1* passed false discovery rate correction in Sanger. Retrospective within-organoid gene ranking modestly improved over lineage-only prediction in both cohorts; top-three overlap improved only in Sanger. Pancreatic results remained unvalidated projections. Findings support weak cross-domain rank transfer and limited within-organoid ranking within the selected panel, without establishing genome-wide transferability, causal explanations for attenuation, or prospective experimental or therapeutic utility.

## Simple Summary

Identifying genes cancer cells need to survive or grow may help researchers choose experimental targets. Direct testing is difficult and costly in organoids, which are small three-dimensional models grown from patient tumors. Using gene activity measurements from 852 conventional cancer models, we built predictive models for a preselected panel of 20 genes. Our primary evaluation asked whether the models could rank organoids by their dependence on each gene. Performance was weaker in organoids, but a small signal remained in 94 Broad organoids and in 148 Sanger organoids from 144 donors. Predictions for the Sanger organoids were fixed before the measured outcomes were accessed. A separate retrospective analysis examined whether the same predictions could rank the 20 genes within each organoid. This retrospective analysis showed modest improvement over tumor-lineage predictions for the full ranking, while improvement for the three strongest measured dependencies was observed only in Sanger. Pancreatic organoid results remained projections because direct measurements were unavailable. These findings provide limited evidence for ranking genes within the selected panel. They do not establish which experiments will succeed or which treatments will work in patients.

## 1. Introduction

Cancer comprises a heterogeneous group of diseases driven not only by genetic and epigenetic alterations but also by the reorganization of cellular signaling networks. This heterogeneity contributes to variable treatment responses among tumors with the same histological diagnosis and limits the effectiveness of target selection based solely on genomic alterations [1]. Gene dependency refers to a cancer cell’s requirement to express a particular gene to survive or proliferate. Identifying such dependencies and synthetic-lethal relationships offers a strategy for exposing cancer-specific vulnerabilities while comparatively sparing normal cells [2].

Large-scale functional genomic studies now enable the systematic interrogation of genes required for fitness across cancer models with diverse molecular backgrounds. The Cancer Dependency Map (DepMap) links molecular profiles of cancer cell lines to functional-screening data, enabling the identification of common and context-specific dependencies [3,4]. Genome-scale loss-of-function screens using clustered regularly interspaced short palindromic repeats (CRISPR) and CRISPR-associated protein 9 (Cas9), collectively termed CRISPR–Cas9, have made systematic gene inactivation feasible in human cells [5,6]. High-resolution libraries have improved discrimination between core-essential genes and context-specific vulnerabilities [7], whereas copy-number-associated cutting toxicity has motivated correction methods such as CERES [8]. Large-scale ribonucleic acid (RNA) interference screens have provided complementary maps of dependency and synthetic lethality [9]. Although Broad and Sanger dependency datasets show broad concordance, gene-effect estimates can vary across screening libraries, experimental protocols, and computational processing [10,11]. Methods such as Chronos seek to improve dependency estimation by accounting for cell-population dynamics [12]. Nevertheless, genome-scale CRISPR screening remains costly, technically demanding, and sample-intensive, limiting its routine application across newly derived cancer models.

The Cancer Cell Line Encyclopedia (CCLE) has established a molecular foundation for modeling associations among mutation, copy number, gene expression, treatment response, and gene dependency [13,14]. Although proteomic profiling expands this information space, messenger RNA expression remains an accessible readout of cellular state, measurable consistently across many cohorts [15]. This availability has motivated the computational prediction of dependencies in samples without direct functional screening. DeepDEP demonstrated that multi-omic profiles can predict cancer dependencies [16], while transcriptome-based dependency maps developed for The Cancer Genome Atlas showed that Elastic Net models can prioritize putative tumor vulnerabilities [17]. Other approaches have used synthetic data augmentation to address incomplete molecular profiles [18]. However, expression-dependency associations can reflect tissue of origin and shared transcriptional programs [19], and domain shifts between cell lines and tumors can compromise transferability [20]. Thus, strong performance within cell-line collections should not be equated with generalizability across biological model systems. Elastic Net is well suited to high-dimensional data because it combines regularization, variable selection, and stability in the presence of correlated features [21]. Yet model selection and performance estimation on the same observations can produce optimistic bias, necessitating nested cross-validation [22]. Likewise, samples derived from the same patient or biological source must remain within a common data partition to prevent information leakage [23].

Conventional two-dimensional (2D) cultures are scalable and well-suited to functional screening, but they incompletely recapitulate cell–cell interactions, tissue architecture, and tumor-specific transcriptional states. Patient-derived organoids are three-dimensional (3D) culture systems that retain selected structural, genetic, and functional features of their tissue of origin [24,25]. Colorectal organoid biobanks have shown that interpatient molecular diversity and drug-response phenotypes can be preserved ex vivo [26]. Comparisons with clinical outcomes also support the potential of gastrointestinal cancer organoids to reflect sensitivity or resistance to selected therapies [27,28]. These advantages make organoids valuable preclinical models for testing whether dependencies learned in 2D remain biologically relevant in more tissue-like systems. However, their epithelial predominance, incomplete representation of immune and stromal compartments, and sensitivity to culture conditions constrain direct extrapolation to patient tumors [29].

In pancreatic cancer, long-term organoid cultures have been established from both normal and neoplastic tissues [30], and growth-factor requirements have been shown to change with disease progression [31]. The pancreatic organoid cohort reported by Tiriac et al. integrated RNA sequencing (RNA-seq) profiles with treatment-response phenotypes, enabling the analysis of molecular and functional subtypes [32]. Subsequent biobanks have further demonstrated the preservation of disease features and the feasibility of individualized drug screening [33]. However, most such cohorts lack genome-scale measured CRISPR dependency outcomes. Consequently, applying an expression-based dependency model to RNA-seq data from these resources can provide biological projections or candidate hypotheses for functional testing, but cannot by itself establish measured gene dependency or therapeutic sensitivity.

Direct functional screening in organoids is technically challenging, yet genome-scale studies in intestinal organoids have identified genes associated with growth and treatment resistance [34]. Pooled screens in human colon organoids have revealed tumor-suppressor and context-dependent selection effects [35], and more recent work has identified dependencies involving WNT, mitogen-activated protein kinase, phosphoinositide 3-kinase, p53, and deoxyribonucleic acid (DNA) mismatch-repair pathways [36]. Large-scale CRISPR knockout, CRISPR interference, and CRISPR activation screens in gastric organoids have further demonstrated the feasibility of examining gene-drug interactions in 3D systems [37]. Next-generation model collections show that dependency profiles may differ between 2D and 3D culture conditions [38]. Cancer dependencies can also be mapped systematically across tumor organoid biobanks [39]. Expanded patient-derived model resources can capture substantial tumor diversity [40]. Together, these studies establish organoids as increasingly tractable functional genomics platforms while also underscoring that culture context can alter the biological relationships inferred from conventional cell lines.

Despite these advances, the extent to which transcriptome-dependency relationships learned in 2D cancer cell lines transfer to independent 3D organoid cohorts remains unresolved. A rigorous assessment requires model selection to be completed without access to 3D outcomes, predictions to be locked before outcome evaluation, and performance to be tested across independently generated CRISPR datasets. Here, we developed target-specific, expression-based Elastic Net models for a fixed panel of 20 genes using 852 pan-cancer 2D models. Candidate molecular representations were compared using patient-grouped nested cross-validation. The selected model was evaluated once in a locked 2D holdout for internal confirmation conditional on historical target selection. Without outcome-guided retraining, the frozen model was then examined exploratorily in Broad organoids and subjected to blinded independent external validation in a non-overlapping Sanger organoid cohort. Target-level findings from the two 3D cohorts were integrated in a retrospective transportability synthesis, and the locked models were subsequently projected onto an independent pancreatic organoid RNA-seq cohort lacking measured CRISPR outcomes. The primary hypothesis concerned ranking organoids by measured dependency for each gene, not ranking genes within an individual organoid. We hypothesized that this between-organoid signal would remain detectable but attenuated in 3D systems. A separate retrospective analysis evaluated gene ranking within individual organoids using the same frozen predictions. This secondary analysis addressed a different prediction task and did not replace the original primary evaluation.

## 2. Materials and Methods

### 2.1. Study Design and Data Sources

**Figure 1:**
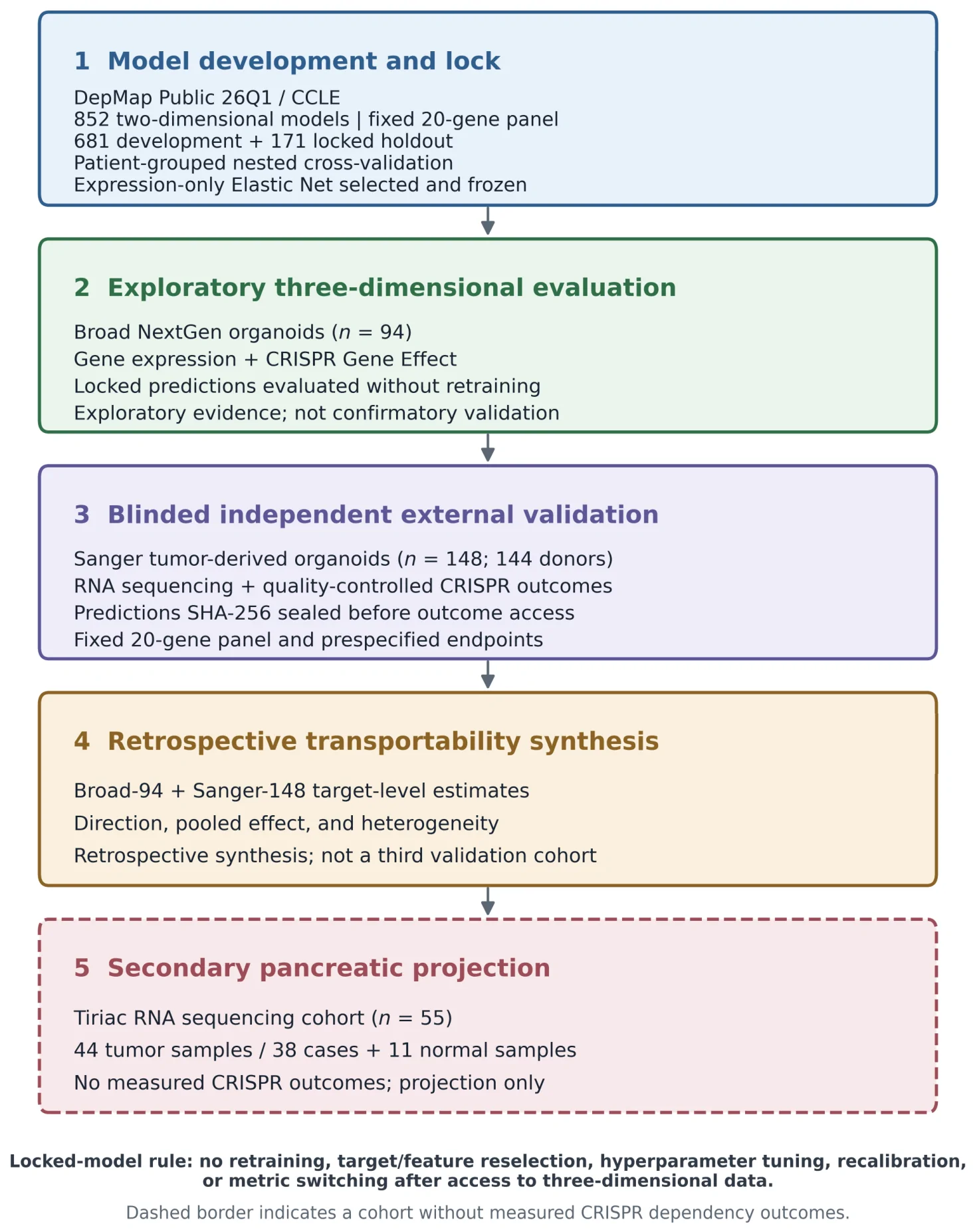
Study design, data sources, and evidence hierarchy. Target-specific Elastic Net models were developed from transcriptomic profiles and CRISPR Gene Effect values in 852 two-dimensional cancer models obtained from DepMap Public 26Q1 and the CCLE. Candidate representations were compared in 681 development models, and the selected expression-only representation was evaluated once on a locked 171-model internal holdout set; the final models were then refitted on all 852 models, keeping the targets, target-specific features, hyperparameters, and primary metric fixed. Historical target selection included the holdout models, so this evaluation provides internal confirmation conditional on the selected panel. Without retraining, these models were applied to 94 Broad NextGen organoids for exploratory evaluation and to 148 Sanger organoids representing 144 donors for blinded independent external validation. Sanger predictions were sealed using the 256-bit Secure Hash Algorithm before access to CRISPR outcomes. Broad and Sanger target-level results were subsequently combined in a retrospective transportability synthesis. The Tiriac pancreatic RNA-sequencing cohort comprised 55 samples and was used only for secondary projection because measured genome-scale CRISPR dependency outcomes were unavailable. The dashed border identifies this non-validation application cohort. The figure depicts the primary workflow; the model specification and prespecified primary metric remained unchanged. The separate retrospective within-organoid ranking analysis is described in Section 2.10.1 and Tables S17–S19 and was not a new blinded validation. Colored boxes distinguish the five labeled stages, and arrows indicate their order in the workflow. CCLE, Cancer Cell Line Encyclopedia; CRISPR, clustered regularly interspaced short palindromic repeats; DepMap, Cancer Dependency Map; RNA, ribonucleic acid; SHA-256, 256-bit Secure Hash Algorithm.

This retrospective, multi-cohort computational study evaluated whether transcriptome-based gene-dependency rankings learned in 2D cancer models could be transferred to independent 3D organoid systems. The study used publicly available or openly released molecular, transcriptomic, and functional-genomics resources; it collected no new biospecimens and performed no new CRISPR screens. The primary workflow comprised five analytically distinct layers: (i) patient-grouped 2D model development and locking; (ii) exploratory transfer assessment in Broad organoids; (iii) outcome-blinded independent external validation in Sanger organoids; (iv) retrospective synthesis of the Broad and Sanger target-level results; and (v) secondary transcriptomic projection in a pancreatic organoid cohort without measured CRISPR dependency outcomes. The primary study design, data sources, and evidence hierarchy are shown in Figure 1. A separate retrospective analysis of gene ranking within organoids was defined after the external outcomes were available (Section 2.10.1; Tables S17–S19).

As shown in Figure 1, the first layer comprised 2D model development and locking using DepMap Public 26Q1 and CCLE molecular data [3,4,13,14]. The analysis-ready population included 852 pan-cancer models with continuous CRISPR Gene Effect values for a fixed 20-gene target panel. Models were partitioned by PatientID, with ModelID used as a fallback only when PatientID was unavailable. This yielded 681 development models from 658 patient groups and 171 locked internal-holdout models from 165 patient groups. No PatientID was shared between partitions. Candidate molecular representations were compared exclusively within the development partition using patient-grouped, lineage-stratified 5 × 5 nested cross-validation. Before the locked-holdout outcomes were evaluated, the selected representation, target panel, target-specific feature identities, hyperparameters, primary metric, and prediction files were frozen. After the one-time holdout evaluation, the selected expression-only Elastic Net specification was refitted on all 852 models for downstream deployment. Only imputation, scaling, and coefficient parameters were re-estimated. The targets, features, hyperparameters, and primary metric remained fixed.

The second layer used 94 organoids from the Broad NextGen cancer model resource, with matched gene expression and CRISPR Gene Effect values for all 20 targets [38]. The all-852 deployment model was applied without Broad-specific retraining, recalibration, feature substitution, or coefficient adaptation. Because PatientID was unavailable in the original Broad evaluation records, the organoid model was initially the analysis and resampling unit. Subsequently recovered partial donor information was examined retrospectively in Section 2.10.3. Although the audited current run preserved a prediction-before-outcome sequence, Broad-94 had been accessible during earlier methodological work and was therefore classified as a retrospective exploratory transfer cohort rather than a pristine independent validation set.

The third layer used the Sanger tumor-derived organoid dependency map [39]. Following cross-resource identity auditing and conservative exclusion of Broad-linked and same-donor records, the final cohort contained 148 organoids from 144 donors. The fixed 20-target prediction matrix, cohort registry, protocol, and model and feature manifests were written and cryptographically sealed using the 256-bit Secure Hash Algorithm (SHA-256) before Sanger CRISPR outcomes were accessed. Sanger-148 was therefore treated as a one-time, outcome-blinded independent external-validation cohort, with donor structure retained in uncertainty estimation and sensitivity analyses.

In the fourth layer, the Broad-94 and Sanger-148 target-level rank correlations were combined only after the two cohorts had been evaluated separately [38,39]. The retrospective transportability synthesis assessed directional concordance, pooled target-level associations, and between-cohort heterogeneity. It was not treated as a third external validation and did not alter any component of the frozen model. Because Broad/DepMap Chronos Gene Effect and Sanger corrected CRISPR log fold change (LFC) values are assay-specific scales, the synthesis focused on rank transfer rather than absolute numerical equivalence.

The fifth layer applied the same frozen models to 55 RNA-seq samples from the Tiriac pancreatic organoid cohort, accessed through the Genomic Data Commons (GDC) ORGANOID-PANCREATIC project [32]. The resource comprised 44 tumor samples from 38 independent tumor cases and 11 normal samples from 11 independent normal cases. Because measured genome-scale CRISPR dependency outcomes were unavailable, this layer was restricted to secondary transcriptomic projection and expression-domain diagnostics and was not used to estimate predictive accuracy or therapeutic sensitivity. Across all layers, no 3D observation contributed to 2D candidate selection, feature learning, hyperparameter tuning, or deployment coefficient estimation. After access to 3D data, no retraining, recalibration, target or feature reselection, hyperparameter retuning, or replacement of the prespecified primary metric was permitted. The secondary within-organoid endpoints were evaluated retrospectively and did not modify the frozen predictions or original primary results. Detailed cohort construction, preprocessing, modeling, and statistical procedures are provided in Section 2.2, Section 2.3, Section 2.4, Section 2.5, Section 2.6, Section 2.7, Section 2.8, Section 2.9 and Section 2.10, and the archived code, frozen models, locked predictions, and verification records are described in Section 2.11.

### 2.2. Development Cohort Construction and Target Panel Definition

The analysis-ready 2D resource comprised 852 pan-cancer models with gene-dependency outcomes available for a fixed 20-gene target panel. Each model was identified by ModelID. To prevent information leakage from biologically related models, we used PatientID as the grouping variable; when PatientID was unavailable, we used ModelID as the fallback group identifier. The patient-grouped partition and fixed target panel are detailed in Table S1.

Using a locked 20% test fraction and global random seed 20260826, we divided the 852 models into 681 development models and 171 locked 2D holdout models, representing 658 and 165 unique patient groups, respectively. No PatientID overlapped between partitions. We used the development partition to compare candidate input representations, learn target-specific features, and select hyperparameters. Within the current model-development workflow, holdout outcomes were not accessed until the development decisions and prediction files had been locked. This restriction did not apply retrospectively to the earlier target-panel selection.

The response matrix comprised continuous CRISPR Gene Effect values for a 20-gene panel established before the present omics-candidate comparison. The panel included *CDK6*, *STK11*, *CCND3*, *YAP1*, *MDM4*, *EP300*, *CTNNB1*, *EGFR*, *TSC2*, *TSC1*, *IGF1R*, *FGFR1*, *CCNE1*, *E2F3*, *ERBB3*, *NFE2L2*, *BRAF*, *KEAP1*, *MAPK1*, and *RAF1*. No target was added to or removed from the panel during the current omics ablation, the Broad and Sanger 3D evaluations, or the Tiriac pancreatic organoid projection.

As documented in Table S1, the panel had been defined in an earlier 2D workflow that used all 852 models. Consequently, the 171-model holdout was independent of the current candidate representation, feature selection, hyperparameters, and model-fitting decisions, but not fully independent of historical target panel selection. Its performance was therefore interpreted as internal confirmation conditional on the selected panel, not an unbiased estimate of generalization for the complete analysis pipeline. Independent 3D validation assessed new samples for the same preselected genes; it did not evaluate transferability to unselected genes or across the genome.

After the one-time holdout evaluation, we refitted the selected representation on all 852 models for downstream 3D deployment while retaining the fixed targets, target-specific feature identities, hyperparameters, and primary metric. Only preprocessing parameters and Elastic Net coefficients were re-estimated, as described in Section 2.5.

### 2.3. Omics Data Preprocessing and Candidate Input Representations

The candidate omics blocks were derived from processed molecular data in DepMap Public 26Q1 and aligned to the 852 models defined in Section 2.2 [3,13,14]. All matrices were ordered by ModelID and verified against the training registry. Gene-level features were tracked using Entrez Gene identifiers. For each target, the response variable was the continuous CRISPR Gene Effect value; no target-dependency outcome was included in any predictor matrix.

The gene-expression block comprised 19,215 protein-coding genes, represented as log2-transformed transcripts per million (TPM) values [log2(TPM + 1)], with no subsequent logarithmic transformation. The copy-number block comprised 19,955 whole-genome sequencing-derived gene-level linear, ploidy-relative values. Somatic variants were encoded separately as binary indicators of damage-mutation and hotspot-mutation carriers for 19,626 genes. OncotreeLineage was encoded as 29 binary indicators. The source blocks, encodings, initial dimensions, and candidate representations are summarized in Table S2.

As shown in Table S2, lineage-only used the 29 lineage indicators as a non-molecular reference. Four molecular candidates were ordered by increasing representational complexity: expression-only (19,215 initial features); expression + copy number (39,170); expression + mutation (58,467), combining expression with damaging and hotspot indicators; and all omics + lineage (78,451), combining all four molecular blocks with lineage. The lineage-only reference was not eligible under the molecular one-standard-error selection rule.

Eligibility screening was relearned within every applicable training partition. Expression and copy-number features were excluded when the missing-value fraction in the training partition exceeded 0.20 or the variance was less than or equal to 1 × 10^−8^. The same variance threshold was applied to mutation indicators and to lineage indicators within the all-omics candidate set; all 29 indicators were retained for the lineage-only reference. For each target, nonfinite values were temporarily replaced with the training-partition means solely for univariate screening; the 300 eligible features with the largest absolute Pearson correlations were then retained.

For model fitting, missing values among the selected features were imputed using training-partition medians and standardized using means and standard deviations learned from the same training partition. These parameters were transferred unchanged to the corresponding validation or test observations. Filtering, screening, imputation, and standardization were repeated within each inner-training fold, each outer-training partition, and the 681-model development fit used for locked-holdout prediction. The 171 locked holdout models and all 3D data were excluded from learning feature eligibility, feature identities, imputation values, and scaling parameters.

### 2.4. Patient-Grouped Nested Cross-Validation and Model Selection

Model development and candidate-representation comparison were performed exclusively in the 681-model development partition. To separate model selection from performance estimation, we used a prespecified 5 outer × 5 inner patient-grouped, lineage-stratified nested cross-validation design [22,23]. PatientID was retained as the group identifier, with ModelID used only as a fallback when PatientID was unavailable. No patient group crossed a training-validation boundary. Within each split, OncotreeLineage categories represented by fewer than five models were collapsed into Other_Rare for stratification only; the original lineage labels remained unchanged for modeling and reporting. Each development model contributed to exactly one outer-test prediction. The full frozen validation and tuning protocol is provided in Table S3.

Within each outer-training partition, a separate five-fold inner validation was constructed. The eligibility filters, target-specific top-300 feature screening, median imputation, and standardization described in Section 2.3 were relearned in every inner-training fold and transferred unchanged to the corresponding inner-validation fold. Outer-test observations contributed to neither feature identities, preprocessing parameters, nor hyperparameter selection. Information flow was restricted to inner training, inner validation, hyperparameter selection, refitting on the complete outer-training partition, and one-time prediction of the outer-test partition.

A separate Elastic Net regression model was fitted for each of the 20 targets and each molecular representation [21]. The frozen search space was l1_ratio = {0.10, 0.50, 0.90} and alpha = {0.001, 0.01, 0.1}; we used a maximum of 20,000 iterations, a convergence tolerance of 0.0001, and cyclic coordinate updates. The inner objective was the mean target-specific Spearman correlation across the five inner-validation folds. We prioritized settings that converged in all inner folds; among them, we selected the setting with the highest mean inner Spearman correlation, resolving remaining ties in favor of a larger alpha and then a lower l1_ratio. When no setting converged in all five folds, we selected based on the number of converged folds, mean inner Spearman correlation, and larger alpha. We refit the chosen setting on the complete outer-training partition and applied it once to the outer-test partition. For the lineage-only reference, target-specific ridge regression was fitted to the 29 lineage indicators, with alpha = {0.1, 1, 10, 100} compared in the inner validation.

Predictions from the five outer-test folds were combined to generate out-of-fold (OOF) predictions for all 681 development models. The primary candidate score was the arithmetic mean of the 20 target-specific Spearman correlations, termed the macro target-wise Spearman correlation. We retained target-specific mean absolute error (MAE), root mean squared error (RMSE), coefficient of determination (R^2^), calibration intercept, and calibration slope as secondary diagnostics, which did not alter model selection. Stability of the target-specific feature sets across outer folds was summarized using pairwise Jaccard similarity.

Candidate selection used only the development OOF predictions. Uncertainty for each candidate was estimated from 2000 bootstrap resamples of PatientID clusters. The one-standard-error threshold was defined as the highest observed molecular-candidate OOF macro Spearman correlation minus the candidate’s bootstrap standard error. Among molecular candidates meeting this threshold, we selected the least complex representation according to the prespecified order: expression-only, expression + copy number, expression + mutation, and all omics + lineage. Lineage-only was a non-molecular reference and was excluded from this rule.

Table S3 further documents the development lock. For the selected candidate, the final l1_ratio and alpha pair for each target was the setting chosen most often across outer folds; ties were resolved by higher mean inner Spearman correlation, then by larger alpha. The 300 target-specific features were relearned using all 681 development models. Before indexing the 171-model outcome matrix for performance evaluation, we sealed the frozen protocol, split registry, selected representation, target-specific feature identities, hyperparameters, model manifest, and locked-holdout predictions using separate SHA-256 digests. No candidate, target, feature, hyperparameter, or primary metric was changed thereafter, and no 3D data were accessed during development.

### 2.5. Locked 2D Holdout Evaluation and Deployment Model Finalization

After development selection had been locked, only the selected expression-only representation was applied to the 171-model 2D holdout. For each target, the 300 expression features were relearned in all 681 development models, and the final target-specific l1_ratio and alpha pair defined in Section 2.4 were fixed. Median imputation, standardization, and Elastic Net coefficients were fitted using the 681 development models and applied unchanged to the 171 holdout models. Predictions for the 20 targets were written together with ModelID, PatientID, and OncotreeLineage. Before the observed holdout CRISPR Gene Effect rows were indexed for evaluation, the prediction files, model manifest, and selected-feature registry were sealed with SHA-256 digests. The one-time holdout evaluation and deployment-model finalization protocol is summarized in Table S4.

The locked holdout outcomes were evaluated once after the prediction lock. The primary endpoint was the macro target-wise Spearman correlation. We calculated target-specific Spearman correlation, MAE, RMSE, R^2^, calibration intercept, and calibration slope as secondary diagnostics. We estimated uncertainty in the macro Spearman correlation from 2000 bootstrap resamples of PatientID clusters; we retained all rows belonging to a sampled patient group together, and the 2.5th and 97.5th percentiles defined the 95% confidence interval (CI). The holdout-minus-development difference distribution was obtained by subtracting the development OOF bootstrap value from the holdout bootstrap value within each iteration. No model, feature, hyperparameter, or metric was changed during evaluation. Because historical target-panel selection used all 852 models, this stage provided internal confirmation conditional on that selection. We did not interpret its performance as an unbiased generalization estimate. The patient-cluster bootstrap did not account for uncertainty or optimism arising from historical target selection.

To evaluate information beyond tumor lineage, the lineage-only ridge reference described in Section 2.4 was refitted in the 681 development models and applied to the same 171 holdout models. The difference in macro Spearman correlation between the selected model and the lineage-only reference was estimated using common PatientID-cluster bootstrap resamples. When more than one model was associated with a patient, observed outcomes and predictions were additionally averaged at the PatientID level. With predictions fixed, complete 20-target outcome vectors were permuted among patient groups within each OncotreeLineage stratum for 2000 iterations. The one-sided empirical *p*-value was calculated as (1 + the number of null statistics greater than or equal to the observed statistic)/2001. The patient-aggregated macro Spearman correlation was retained as a sensitivity estimate.

After completing the locked 2D evaluation, we refitted the final model authorized for 3D application to all 852 models. The expression-only representation, fixed 20-target panel, 300 target-specific feature identities, target-specific l1_ratio and alpha settings, and primary metric were not reopened. We re-estimated only the median-imputation values, standardization parameters, and Elastic Net coefficients. Within the current model-fitting workflow, holdout outcomes entered coefficient estimation only in this final all-852 fit, after the internal performance estimate had been fixed. They were not used to revise candidate selection, targets, features, hyperparameters, or the reported 681-to-171 evaluation. A separate serialized bundle was recorded for each target, including feature identities and types, the imputer, scaler, fitted estimator, training-sample count, model role, and protocol hash. Nonconverged deployment fits were not accepted.

As documented in Table S4, the locked internal-evaluation models and the all-852 deployment models were analytically distinct. The former supported only the 681-to-171 performance estimate; the latter constituted the final models applied without retraining to the 3D cohorts. The deployment-model manifest, selected-feature registry, target-specific model bundles, and final lock records were SHA-256 sealed before access to any new 3D data. Subsequent 3D applications prohibited retraining, recalibration, target or feature reselection, hyperparameter retuning, and metric switching; blind predictions had to be written and cryptographically sealed before outcome merging.

### 2.6. Broad-94 Organoid Harmonization and Exploratory 3D Evaluation

The Broad-94 exploratory 3D cohort comprised 94 organoid models from the Broad NextGen resource, with both gene expression data and CRISPR Gene Effect outcomes for all 20 locked targets [38]. ModelID was used for deterministic sample alignment and analysis. PatientID was unavailable in the original Broad evaluation records; therefore, the original analysis used organoid models as the inferential and resampling units. A later audit recovered partial donor information and identified a same-donor pair; the corresponding retrospective sensitivity analyses are described in Section 2.10.3 and Tables S22–S24. The frozen harmonization, prediction-lock, and exploratory-evaluation protocol is summarized in Table S5.

Cohort-size reconciliation found 95 unique 3D organoid (3DO) models in the archived Broad screen metadata. One model, ACH-003044, had PassesQC = False; the 94 quality-control-passing ModelIDs exactly matched the frozen registry. The prediction and outcome matrices each contained 94 × 20 values, and the long-format results contained 1880 unique model–target pairs. These records support the 94-model analytical cohort (Table S22).

The expression-only deployment model, finalized in Section 2.5 and refitted to all 852 2D models, was applied without Broad-specific retraining. For each target, the 300 locked expression features were matched to the Broad expression matrix using Entrez Gene identifiers and ordered exactly as stored in the corresponding model bundle. Feature coverage was defined as the proportion of the 300 frozen predictors present in the external expression matrix for each target. The prespecified 80% minimum (at least 240 of 300 predictors) was a pragmatic input-eligibility rule that limited unavailable predictors to 20% while allowing incomplete gene coverage across data resources. The cutoff was not optimized against external predictive performance and does not establish reliable prediction at 20% missingness. All 300 features were available for every Broad target, yielding 100% target-specific coverage. We applied the 2D-fitted median-imputation values, standardization parameters, Elastic Net coefficients, and l1_ratio and alpha settings unchanged. No Broad-fitted imputation, scaling, feature selection, coefficient adaptation, or target substitution was performed. This procedure generated a finite 94-organoid × 20-target prediction matrix.

To preserve an auditable prediction-before-outcome sequence in the current run, the prediction matrix and the model, feature, and lock manifests were written and SHA-256 sealed before the observed 3D outcome matrix was extracted. After sealing, only the 20 fixed Gene Effect columns were aligned to the predictions by ModelID. No model component or prespecified primary endpoint was changed after access to the outcome; the retrospective within-organoid endpoints are reported separately in Section 2.10.1. Nevertheless, because Broad-94 had been used in prior methodological work, we did not classify the cohort as a previously unseen, blinded independent validation set; instead, we retained it for a retrospective exploratory assessment of 3D transportability.

The primary estimand was the macro target-wise Spearman correlation. We calculated target-specific Spearman correlation, MAE, RMSE, R^2^, calibration intercept, and calibration slope as secondary diagnostics and did not use them to reopen model selection. We estimated macro- and target-specific uncertainty from 5000 model-level bootstrap resamples performed with replacement within OncotreeLineage strata, preserving the observed lineage counts; 95% CIs were defined by the 2.5th and 97.5th percentiles. The lineage-only ridge reference was applied to the same 94 organoids, and the expression-only-minus-lineage difference was estimated using paired bootstrap resamples. Target-specific partial Spearman correlations, adjusted for lineage indicators; lineage subgroup estimates for groups containing at least 7 models; and leave-one-lineage-out analyses were retained as secondary sensitivity analyses.

For the panel-wide permutation test, predictions were held fixed, and each organoid’s complete 20-target outcome vector was permuted among model rows within the same OncotreeLineage stratum for 5000 iterations, thereby preserving cross-target dependence and lineage composition. The one-sided empirical *p*-value was calculated as (1 + the number of null macro Spearman statistics greater than or equal to the observed statistic)/5001. Target-specific permutation *p*-values were adjusted across the 20 targets using the Benjamini–Hochberg false discovery rate (FDR) procedure. All Broad resampling procedures used random seed 20260829. As specified in Table S5, rank transportability was the primary endpoint. Absolute-scale error, calibration, and lineage sensitivity were secondary diagnostics. Broad results were not used to retrain the model or select targets based on effect direction or statistical significance.

### 2.7. Sanger-148 Cohort Construction and Blinded Independent External Validation

The independent Sanger 3D validation cohort was constructed from the RNA-seq and CRISPR-screening records of the tumor-derived organoid dependency map [39]. Before any Sanger target outcome was accessed, 162 organoids from 157 unique donors with both RNA-seq profiles and quality-controlled CRISPR-screening records were screened for eligibility. To prevent reuse of samples represented in the Broad exploratory layer or elsewhere in the Broad NextGen resource, Sanger sample and donor identifiers were cross-referenced against the Broad mapping records and the Broad-94 registry [38,39]. Thirteen Broad-linked organoids and one additional organoid from the same donor as a Broad-linked model were conservatively excluded. The final cohort therefore comprised 148 organoids from 144 unique donors, with no exact Broad-94 identity or identifiable same-donor Broad overlap. Cohort construction, harmonization, and the outcome-blinded lock procedure are summarized in Table S6.

The final cohort included five tumor types: 81 colorectal organoids from 79 donors, 49 esophageal organoids from 49 donors, 11 ovarian organoids from 9 donors, 4 pancreatic organoids from 4 donors, and 3 gastric organoids from 3 donors. RNA-seq input and experimental CRISPR coverage for all 20 locked targets were available for every included organoid. Colorectal and esophageal subgroup analyses were prespecified as secondary, the ovarian subgroup was exploratory, and the pancreatic and gastric subgroups were treated as descriptive because of their small sample sizes. The primary external-validation analysis used all 148 organoids, with donor structure preserved in uncertainty estimation and sensitivity analyses.

Sanger TPM values were transformed as log2(TPM + 1) and aligned to the frozen expression-only feature space using Entrez Gene identifiers. For each target, the 300 expression features stored in the corresponding 2D deployment-model bundle were used in their locked order. Across targets, 3790 of 3845 unique locked genes were measured in the Sanger matrix; 55 genes were absent. Target-specific coverage ranged from 291 to 300 of 300 features (97.0–100%), exceeding the prespecified 80% minimum for all targets. Missing features were neither re-estimated from the external cohort nor replaced using Sanger-derived values; only the imputation and standardization objects learned in 2D data and the fixed Elastic Net coefficients were applied. A pre-outcome audit identified that the original coverage-key array had incorrectly counted the 55 entirely absent genes as present. The coverage metadata were corrected before outcome access, while the original and corrected blinded predictions remained bitwise identical.

Before the Sanger CRISPR outcomes were opened, the 148-organoid × 20-target blinded prediction matrix, cohort registry, protocol, model manifest, and feature manifest were written and sealed with SHA-256 digests; all 11 prespecified seal checks passed. Only after this lock was established were the Sanger outcomes accessed once and merged with the predictions in the frozen sample and target order, yielding 2960 finite prediction-outcome pairs. The external outcome was the corrected and scaled gene-level CRISPR LFC, with more negative values indicating stronger dependency. For the 13 organoids screened with two CRISPR libraries, the official Sanger covariate was applied: minLib was selected when the code was 1 and v1_1 when the code was 0. The available quality-controlled screen was used for single-library organoids. This externally defined selection rule was fixed before inspection of the target outcomes.

The primary external-validation estimand was the target-specific Spearman correlation between the blinded predictions and Sanger LFC values, followed by the arithmetic mean of the 20 target correlations (macro target-wise Spearman correlation). This primary estimand evaluates the ordering of organoids for a fixed gene and does not assess the ordering of genes within an organoid. Rank correlation was selected because the Sanger-corrected LFC and DepMap/Broad Chronos Gene Effect values are on assay-specific scales and were not assumed to be numerically comparable. Uncertainty was estimated with 5000 donor-cluster bootstrap resamples, retaining all organoids from a sampled donor together; 95% CIs were obtained from the 2.5th and 97.5th percentiles. For the null distribution, repeated organoids were first averaged within donor, predictions were held fixed, and each donor-level 20-target observed-outcome vector was permuted within tumor-type/lineage strata for 5000 iterations. The two-sided empirical *p*-value was calculated as (1 + the number of absolute null macro correlations at least as large as the observed absolute macro correlation)/5001. Target-specific permutation *p*-values were adjusted across the 20 targets using the Benjamini–Hochberg FDR procedure.

A lineage-only comparator, frozen before access to Sanger outcomes and based on patient-weighted 2D lineage means, was applied to the same cohort. We evaluated the expression-only-minus-lineage macro difference using paired donor-cluster bootstrap resamples, and we also calculated partial Spearman correlations adjusted for the primary tumor-type indicators. The influence of repeated donors was examined in two additional analyses: one retained the lexicographically first sample identifier per donor, and the other averaged predictions and outcomes within donor. An independent replay recomputed cohort size, donor count, overlap exclusions, feature coverage, target and prediction order, the complete 2960-pair grid, correlation statistics, bootstrap and permutation results, lineage adjustment, and file hashes; all 22 checks passed. As detailed in Table S6, no model, target, feature, hyperparameter, or prespecified primary metric was changed after outcome unlock. The retrospective within-organoid endpoints were evaluated separately using the unchanged predictions.

### 2.8. Retrospective Cross-Cohort Transportability Synthesis

The target-specific results from the Broad-94 exploratory evaluation and the Sanger-148 blinded independent external validation were combined retrospectively only after the two cohort-specific analyses had been completed [38,39]. The fixed 20-target panel defined in Section 2.2 was aligned using Entrez Gene identifiers, and no targets were removed based on effect direction, magnitude, or statistical significance. Because Broad used Chronos Gene Effect values and Sanger used corrected CRISPR LFC values, the synthesis addressed target-specific rank transportability rather than absolute-scale equivalence. The Tiriac pancreatic projection was excluded from this synthesis because it did not contain measured genome-scale CRISPR outcomes. The synthesis protocol is summarized in Table S7.

Directional concordance was defined as a positive Spearman correlation for the same target in both Broad-94 and Sanger-148. The number of common-positive targets was compared with a one-sided exact binomial distribution with *n* = 20 and a null success probability of 0.50. This comparison was interpreted only as a descriptive sign benchmark: dependence among targets limits the binomial interpretation, and the synthesis was defined after the cohort-specific results were known. Agreement in relative target performance was evaluated separately using the two-sided Spearman correlation between the vectors of 20 Broad and 20 Sanger target-specific correlations.

For target-level pooling, each cohort-specific Spearman correlation was transformed to the Fisher-z scale. We derived inverse-variance weights from the cohort-specific uncertainty intervals obtained from the lineage-stratified Broad bootstrap and the donor-clustered Sanger bootstrap. Because Broad donor structure was incompletely ascertained, its original uncertainty weights may overstate precision; the synthesis is conditional on this unresolved model-level independence assumption. A fixed-effects pooled estimate was calculated for each target and then transformed back to the Spearman correlation scale. The resulting two-sided meta-analytic *p*-values were adjusted across the 20 targets using the Benjamini–Hochberg FDR procedure. Because only two 3D cohorts were available, random-effects estimates and the I^2^ heterogeneity statistic were retained as descriptive sensitivity measures and were not used as confirmatory decision criteria.

The panel-wide combined 3D estimate was defined as the arithmetic mean of the 20 fixed-effect target correlations. Sensitivity to target-panel composition was assessed by resampling the 20 locked targets with replacement for 20,000 iterations, then recalculating the combined macro correlation at each iteration. We reported the 2.5th and 97.5th percentiles as a descriptive 95% interval; this interval characterizes sensitivity to the composition of the fixed target panel and is not a new-patient or new-organoid sampling interval. Attenuation from 2D to 3D was calculated for each target by subtracting the corresponding locked 2D holdout correlation from the Broad or Sanger correlation, with panel-level summaries obtained using the same target-bootstrap structure.

To evaluate whether cross-target dependence could explain the common-positive count, we performed a post hoc, correlation-aware simultaneous permutation analysis. Predictions remained fixed. In Broad, each ModelID-level 20-target observed-outcome vector was retained as a block; in Sanger, repeated organoids were first averaged within donor, and each donor-level 20-target outcome vector was retained as a block. These vectors were permuted simultaneously among units within each cohort for 50,000 iterations, preserving the cross-target covariance structure. We recalculated the number of targets remaining positive in both cohorts at each iteration and calculated the one-sided empirical *p*-value with the plus-one correction. This analysis was explicitly treated as a post hoc sensitivity analysis and did not constitute a third external validation.

Within each cohort, we subtracted the target-specific lineage-only correlation from the corresponding expression-only correlation. We used a one-sided Wilcoxon signed-rank test on the 20 paired differences to assess the incremental contribution of expression beyond lineage. A retrospective evidence classification was used for reporting only. Tier A required a positive correlation in both cohorts, a meta-analytic FDR q < 0.05, and target-level FDR q < 0.05 in at least one individual cohort. Tier B indicated positive correlations in both cohorts without fulfillment of all Tier A criteria. Tier C indicated a non-positive correlation in at least one cohort. As specified in Table S7, the evidence tiers did not alter the locked panel or any model component, and no retraining, target or feature reselection, hyperparameter tuning, metric switching, or direction-based target removal was permitted during the synthesis.

### 2.9. Secondary Transcriptomic Projection in the Tiriac Pancreatic Organoid Cohort

After completion of 2D model development, Broad-94 exploration, Sanger-148 blinded validation, and the retrospective cross-cohort synthesis, the frozen expression-only deployment models were applied to the Tiriac/ORGANOID-PANCREATIC cohort as a secondary biological projection [32]. Because measured genome-scale CRISPR dependency outcomes were unavailable, this analysis was prespecified as an application to an independent pancreatic transcriptome rather than external validation; predictive accuracy and CRISPR performance were not estimated. Fifty-five publicly available RNA-seq Spliced Transcripts Alignment to a Reference (STAR) count files from the GDC project were obtained from the official public Amazon Simple Storage Service mirror. The cohort comprised 44 tumor samples representing 38 independent tumor cases and 11 samples from 11 independent normal cases; group membership was defined from the source TissueType metadata. Cohort preparation and projection procedures are summarized in Table S8.

The tpm_unstranded field in the STAR output was used as the abundance measure. Rows mapping to the same gene symbol were summed at the TPM level before log2(TPM + 1) transformation. The resulting sample-by-feature matrix was aligned to the locked expression features of the 20 target-specific Elastic Net deployment bundles finalized in all 852 2D models. The target panel, target-specific feature identities, stored imputation and standardization objects, hyperparameters, coefficients, and projection definitions were applied without modification. No Tiriac sample contributed to model fitting, feature selection, recalibration, retuning, or endpoint definition.

Of the 3845 unique locked features used across the 20 models, 3806 were observed in the Tiriac data. Target-specific coverage ranged from 292 to 300 of 300 selected features (97.33–100%), and every target met the prespecified 80% coverage threshold. Features absent from the Tiriac matrix were represented as not-a-number (NaN) values, filled with the medians stored in the frozen all-852 deployment models before the stored standardization, and then coefficients were applied. This preserved the training preprocessing rule without estimating replacement values from Tiriac. An independent computational replay reproduced the archived predictions exactly using training-median imputation (Section 2.10.2). This procedure produced a 55-sample × 20-target projection matrix. Source file identifiers, file sizes, source-provided integrity values, local SHA-256 digests, model and feature manifests, and prediction records were retained for provenance.

When multiple tumor organoid samples originated from the same GDC case, the 20 sample-level projections were averaged within the case, and the patient/case was used as the inferential unit. For each target, median case-level projections were calculated for 38 tumor cases and 11 normal cases, together with the tumor-minus-normal median difference. Uncertainty in this difference was estimated using 5000 case-level bootstrap resamples within the tumor and normal groups, with 95% percentile intervals. Tumor and normal projection distributions were compared using two-sided Mann–Whitney U tests, and the 20 *p*-values were adjusted using the Benjamini–Hochberg FDR procedure. Cliff’s delta was reported as an effect-size measure; under the prespecified orientation, a positive value indicated a more negative, and therefore stronger, projected dependency distribution in tumors. These analyses compared frozen model outputs rather than measured dependencies or model errors.

The 20-target median projection vector for Tiriac tumor cases was compared separately with the corresponding median prediction vectors for Broad-94 and Sanger-148. This comparison assessed whether the projected pancreatic target hierarchy was consistent across organoid applications. Concordance was quantified using two-sided Spearman correlations across the 20 targets. Because all compared values were predictions from the same locked model family and Tiriac lacked measured CRISPR outcomes, these correlations were interpreted as secondary evidence of target-ranking consistency rather than independent evidence of predictive accuracy.

Expression-domain shift was evaluated across the 3790 locked expression features that are jointly available in Tiriac, Broad-94, and Sanger-148. Multivariable separation among cohorts was visualized by principal component analysis (PCA). For Tiriac versus Broad-94 and Tiriac versus Sanger-148, a standardized mean difference (SMD) was calculated for each common feature, and shift magnitude was summarized using the median absolute SMD and the proportions of features with |SMD| > 1 and |SMD| > 2. The PCA and SMD diagnostics were descriptive and were not used to recalibrate the models, delete features, remove targets, or revise projections. Table S8 defines the corresponding claim boundary: Tiriac results were described as projections or prioritizations and not as validated dependencies, confirmed dependencies, or therapeutic sensitivity.

### 2.10. Statistical Analysis

Statistical analyses preserved the prespecified role and strongest available biological independence unit of each study layer. In the development, locked 2D holdout, Broad-94, and Sanger-148 analyses, the primary rank-based measure was the Spearman correlation between predicted and observed outcomes for each target and the arithmetic mean of the 20 target correlations (macro target-wise Spearman correlation). MAE, RMSE, R^2^, calibration intercept, and calibration slope were secondary diagnostics in the development, locked-holdout, and Broad analyses and did not reopen model selection. Because Sanger-corrected LFC and DepMap/Broad Chronos Gene Effect values were not treated as numerically equivalent, the Sanger validation and cross-cohort synthesis were based on rank correlation rather than absolute-scale error. Target-wise correlation operates across organoids for each gene; within-organoid correlation instead operates across the 20 genes for each organoid. Averaging target-wise correlations does not test within-organoid gene ranking. The original analysis hierarchy, inferential units, resampling procedures, and multiplicity rules are summarized in Table S9; the retrospective within-organoid endpoints are defined in Section 2.10.1 and Tables S17–S19.

Resampling units followed the available biological grouping. Development and locked 2D holdout uncertainty used 2000 PatientID-cluster bootstrap replicates, retaining all models from a sampled patient group together. Broad-94 used 5000 model-level bootstrap replicates within OncotreeLineage strata because PatientID was unavailable in the original evaluation records; the later partial-donor sensitivities are reported separately in Section 2.10.3. Sanger-148 used 5000 donor-cluster bootstrap replicates, retaining all organoids from a sampled donor together. Tiriac tumor-normal comparisons used 5000 case-level bootstrap replicates within the 38-tumor and 11-normal groups. Unless otherwise specified, 95% percentile CIs were defined by the 2.5th and 97.5th percentiles. Paired model comparisons used common resampling indices. The standard error used in the development one-standard-error rule was derived from the 2000-replicate PatientID-cluster bootstrap distribution of OOF predictions.

For permutation analyses, predictions remained fixed and complete 20-target observed-outcome vectors were moved without disrupting the prespecified biological grouping. The locked 2D sensitivity analysis used 2000 one-sided permutations of patient-aggregated outcome vectors within OncotreeLineage strata. Broad-94 used 5000 one-sided permutations of ModelID-level outcome vectors within lineage. Sanger-148 used 5000 two-sided permutations after donor averaging, with donor-level outcome vectors moved within tumor-type/lineage strata and the absolute macro correlation used as the test statistic. Empirical *p*-values used the standard plus-one numerator and denominator correction. Broad and Sanger target-specific permutation *p*-values were adjusted separately within their 20-target families using the Benjamini–Hochberg FDR procedure.

In the retrospective cross-cohort synthesis, common-positive direction was assessed with a one-sided exact binomial benchmark (*n* = 20; null probability = 0.50), interpreted descriptively because targets were correlated and the synthesis was retrospective. Target-specific correlations were pooled on the Fisher-z scale using inverse-variance fixed-effect weights derived from cohort-specific bootstrap uncertainty, and the 20 two-sided meta-analytic *p*-values were adjusted using the Benjamini–Hochberg procedure. Random-effects estimates and I^2^ were descriptive because only two cohorts were available. Target-panel sensitivity used 20,000 target-bootstrap resamples, while cross-target dependence was assessed using 50,000 one-sided simultaneous block permutations at the Broad ModelID and Sanger donor-mean levels. Expression-only-minus-lineage target differences were evaluated separately in Broad and Sanger using one-sided Wilcoxon signed-rank tests. None of these retrospective analyses was interpreted as a new validation cohort.

In the Tiriac layer, two-sided Mann–Whitney U tests compared patient-level tumor and normal projections for each target. Benjamini–Hochberg FDR adjustment was applied across 20 tests. Cliff’s delta described effect direction. Concordance between the Tiriac tumor target-median vector and the Broad and Sanger target-median prediction vectors was evaluated using two-sided Spearman correlations across 20 targets. We used PCA and SMD measures only as descriptive domain-shift diagnostics, and they did not inform the confirmatory decision.

A retrospective coverage-threshold sensitivity analysis compared the prespecified 80% rule with stricter 90% and 95% requirements using the archived target-specific present-feature counts. The number of eligible models among the fixed 20 targets was recorded for each external cohort. Only the eligibility requirement was varied; no features were removed, no models were refitted, and no missing-feature handling was changed (Table S16).

Tests were two-sided unless explicitly prespecified as one-sided. Within each stated 20-target family, FDR q < 0.05 defined the multiplicity-adjusted target-level significance; exact *p*-values, effect sizes, and CIs were reported together where applicable. Exploratory and post hoc analyses retained those labels even when a statistical threshold was crossed, and no analysis result changed the target panel, feature identities, hyperparameters, primary metrics, or previously generated predictions. Analyses were implemented in Python 3.12.13 using NumPy 2.3.5, pandas 2.2.3, SciPy 1.17.0, scikit-learn 1.8.0, and joblib 1.5.3. Random seeds, environment files, protocols, SHA-256 manifests, and integrity checks were preserved in the archived reproducibility package.

#### 2.10.1. Retrospective Evaluation of Gene Ranking Within Organoids

A retrospective secondary analysis evaluated whether the frozen expression-only predictions ranked the 20 genes within individual organoids in agreement with measured CRISPR outcomes. This secondary analysis was defined after the external outcomes were available and was not a new blinded validation. It retained all 20 targets and all 94 Broad and 148 Sanger organoids; Tiriac was excluded because measured dependency outcomes were unavailable. No predictions, models, features, hyperparameters, or targets were changed, and the original target-wise primary analysis was retained.

For each organoid, we calculated Spearman correlation across its 20 predicted and 20 observed scores, using average ranks for ties, and summarized the correlations by their arithmetic mean. Lower scores denoted stronger measured or predicted dependency. The main comparator assigned every organoid the same per-gene mean of the observed outcomes across all 852 2D training models, with each training model weighted equally. This fixed ranking tested whether expression predictions improved on gene-level average dependency. We also reused the archived lineage-only predictions: the ridge reference in Broad and patient-weighted 2D lineage means in Sanger. No external outcomes were used to fit either comparator (Table S17).

To examine a short candidate list, we calculated the overlap between the three lowest predicted scores and the three lowest measured scores, divided by three, within each organoid. The cohort mean was expressed as a percentage. Ties at the selection boundary were to be resolved by the fixed target identifiers; none occurred. Expression-minus-comparator differences were calculated within the same organoids for both the full-ranking correlation and top-three overlap (Table S18). These endpoints assess agreement with measured CRISPR score ordering within the selected panel, not cancer selectivity, cross-gene calibration, drug response, or clinical benefit.

We estimated 95% percentile CIs using 10,000 paired bootstrap resamples: organoid models sampled within observed lineage strata for Broad, and donor clusters sampled with all their organoids retained together for Sanger. The Sanger main estimate remained organoid-weighted. A donor-level sensitivity analysis first averaged the predicted and observed 20-gene vectors within each of 144 donors, then recalculated both endpoints with equal donor weights and a 10,000-replicate donor bootstrap (Table S19). Seeds were 20260928, 20260929, and 20260930 for Broad, Sanger, and donor-mean sensitivity, respectively. All correlations were defined. Intervals were conditional on the frozen models and fixed panel; they did not include model-training or gene-panel selection uncertainty. The secondary comparisons were not adjusted for multiplicity and were interpreted as exploratory.

#### 2.10.2. Retrospective Sensitivity to Missing-Feature Imputation in Tiriac

A retrospective sensitivity analysis evaluated the effects of alternative missing-feature replacement values on Tiriac projections. This descriptive analysis was not prespecified. The archived code represented absent inputs as NaN and applied the frozen training-median imputer, followed by the stored scaler and Elastic Net estimator. This pipeline reproduced all 1100 archived Tiriac predictions exactly. The reference predictions were generated with training medians and were retained for comparison with the alternative imputation methods. The 39 absent unique features corresponded to 0–8 missing predictors per target, with the same missing-feature pattern across all 55 samples.

Using the same frozen models, we compared training-median imputation with two alternatives: zero on the log2(TPM + 1) scale before standardization, and the stored training-reference mean for each feature. These means were estimated after median imputation in the 852-model training data and correspond to zero standardized input, up to floating-point rounding. Only absent features were replaced; observed expression values, including measured zeros, were retained. We summarized absolute projection changes, Spearman correlation across the 20 genes within each profile, and top-three set overlap relative to the median-imputed reference. We evaluated sample-level results and case-averaged profiles, including 38 tumor and 11 normal cases. We also repeated the original tumor–normal comparisons using the same 5000 bootstrap resamples (seed 20260830), Mann–Whitney tests, and 20-target FDR adjustment. These are descriptive checks of projection stability, not prediction accuracy. Because the models are linear and the missing-feature pattern is shared, constant replacement values create a common offset per target; preservation of within-target sample ranks and tumor–normal differences is therefore expected algebraically (Tables S20 and S21).

#### 2.10.3. Retrospective Broad Donor Audit and Sensitivity Analyses

A retrospective metadata audit linked 16 of the 94 models to 15 PatientID values in the supplied DepMap Model.csv and nine additional models to nine source-specific donor identifiers in the archived Sanger exclusion registry. Thus, donor information was available for 25 models, forming 24 source-specific groups, while 69 models remained unresolved. ACH-002883 and ACH-002884 shared PatientID PT-N65Hvq. The DepMap and Sanger donor namespaces could not be fully reconciled, so the 24 groups do not establish 24 independently verified biological donors. No donor identity was inferred from lineage or model-name similarity (Table S22).

Retrospective sensitivity analyses used the unchanged predictions, outcomes, and 20-gene panel. Each member of the known same-donor pair was omitted in turn. A conditional full-cohort bootstrap grouped the known pair and treated unresolved models as singleton units, yielding 93 resampling groups; this assumption does not establish independence of the 69 unresolved models. In the donor-documented subset, model-level and donor-cluster bootstraps were compared on the same 25 models, and a separate analysis averaged prediction, outcome, and lineage-reference vectors within the 24 source-specific donor groups before evaluation. All analyses used 5000 paired bootstrap replicates within the observed OncotreeLineage strata (seed 20260929), retaining every model from each sampled group. Percentile 95% intervals were calculated at 2.5% and 97.5%. Target-wise correlations were reranked within each resample and averaged across all 20 targets; an undefined target correlation contributed zero, following the archived convention. Organoid weighting was retained except in the donor-mean analysis. Full-cohort and known-subset model-level replays with the same seed provide matched references; they do not replace the original CIs. Tables S23 and S24 report the primary target-wise and secondary within-organoid endpoints. These post hoc intervals were not adjusted for multiple sensitivity comparisons, and no new confirmatory permutation or FDR claim was made.

### 2.11. Code and Reproducibility Resources

The study’s reproducibility package is available on Zenodo (https://doi.org/10.5281/zenodo.22472622). The package includes custom analysis and figure-generation scripts; frozen cohort, split, target, and feature registries; trained model artifacts; locked predictions; analysis-ready inputs and processed outputs; bootstrap and permutation results; figure source data; software-environment records; and SHA-256 integrity manifests. It also contains the code, inputs, and results for the primary analyses, retrospective within-organoid gene ranking, Tiriac missing-feature imputation sensitivity, and Broad cohort-size and donor sensitivity analyses. Complete original third-party data releases remain available from the repositories identified in the Data Availability Statement.

## 3. Results

### 3.1. Selection of the Expression-Only Model in the 2D Development Cohort

**Figure 2:**
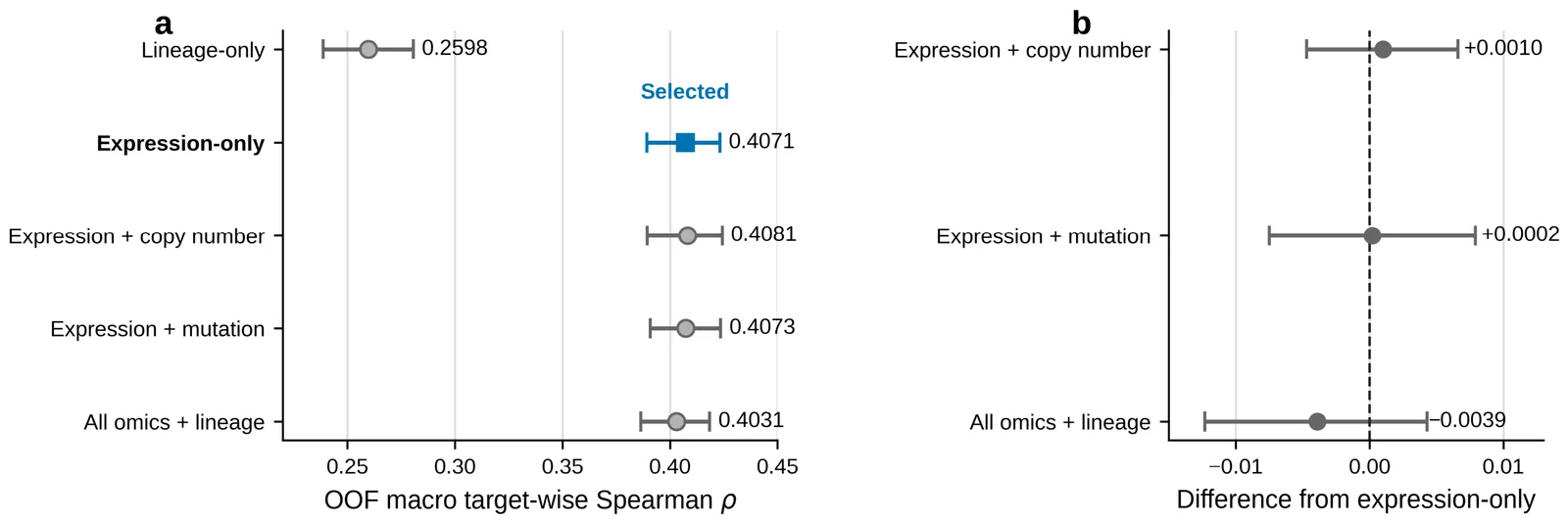
Development-cohort omics ablation and selection of the expression-only model. (**a**) Patient-grouped OOF macro target-wise Spearman correlations for the lineage-only reference and four molecular candidate representations in the 681-model development cohort. Error bars show 95% percentile confidence intervals from 2000 PatientID-cluster bootstrap replicates. The blue square denotes the expression-only candidate selected by the prespecified one-standard-error rule. (**b**) Paired differences in macro Spearman correlation between each augmented molecular representation and expression-only. Error bars show 95% paired PatientID-cluster bootstrap intervals; the vertical dashed line denotes no difference. CI, confidence interval; OOF, out-of-fold.

Five input representations—the lineage-only reference and four molecular candidates—were compared in the development partition of 681 models representing 658 patient groups, using identical patient-grouped outer folds. Candidate-level OOF macro target-wise Spearman correlations and paired differences relative to expression-only are shown in Figure 2a,b; full candidate- and target-level results are provided in Table S10.

As shown in Figure 2a, the highest observed OOF macro target-wise Spearman correlation was obtained with expression + copy number (ρ = 0.4081; 95% CI, 0.3894–0.4243). Performance was nearly identical for expression-only (ρ = 0.4071; 95% CI, 0.3893–0.4232), expression + mutation (ρ = 0.4073; 95% CI, 0.3908–0.4235), and all omics + lineage (ρ = 0.4031; 95% CI, 0.3864–0.4184). The lineage-only reference produced a lower macro correlation (ρ = 0.2598; 95% CI, 0.2388–0.2806) and, as prespecified, was not eligible for molecular candidate selection.

Relative to expression-only, paired PatientID-cluster bootstrap differences were +0.0010 (95% CI, −0.0047 to 0.0066) for expression + copy number, +0.0002 (95% CI, −0.0075 to 0.0079) for expression + mutation, and −0.0039 (95% CI, −0.0123 to 0.0043) for all omics + lineage (Figure 2b). All three intervals included zero, providing no evidence that adding copy-number, mutation, or combined omic and lineage information produced a reliable improvement in development performance.

The expression-only candidate remained within one bootstrap standard error of the highest-scoring molecular candidate and showed the greatest mean feature-set Jaccard stability across outer folds (0.565 versus 0.550–0.554 for the other molecular candidates). The prespecified one-standard-error rule therefore selected expression-only as the final molecular representation.

### 3.2. Locked 2D Holdout Performance and Deployment Model Finalization

**Figure 3:**
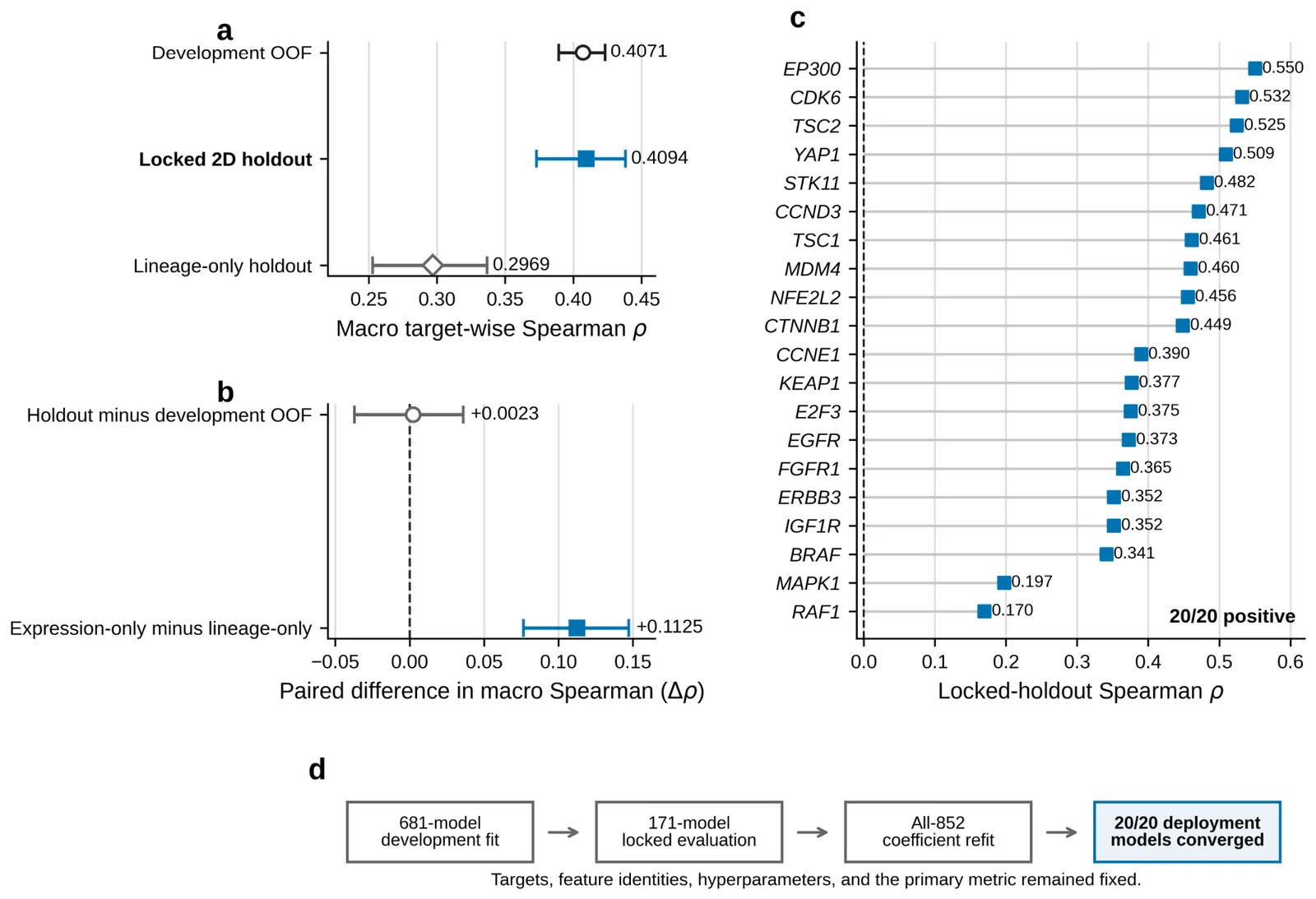
Locked two-dimensional holdout performance and deployment-model finalization. (**a**) Development OOF, locked two-dimensional holdout, and lineage-only holdout macro target-wise Spearman correlations. (**b**) Prespecified paired contrasts. Error bars in (**a**,**b**) show 95% intervals from 2000 PatientID-cluster bootstrap replicates. (**c**) Target-specific locked-holdout correlations, ordered from lowest to highest; all 20 were positive. (**d**) Finalization sequence. After the one-time holdout evaluation, the winning model was refitted on all 852 two-dimensional models while keeping the target panel, target-specific feature identities, hyperparameters, and primary metric fixed; all 20 deployment models converged before three-dimensional use. The target panel had been selected using the full source population, so holdout performance represents internal confirmation conditional on that selection, not an unbiased generalization estimate. In (**a**), the open circle, blue square, and open diamond denote the development OOF, locked 2D holdout, and lineage-only holdout estimates, respectively. In (**b**), the open circle denotes holdout minus development OOF, and the blue square denotes expression-only minus lineage-only. In (**c**), blue squares mark target-specific correlations, with gray horizontal lines connecting each estimate to zero. Dashed vertical lines in (**b**,**c**) mark zero. Arrows in (**d**) indicate the finalization sequence. 2D, two-dimensional; CI, confidence interval; OOF, out-of-fold.

After model selection was locked, the expression-only model, target-specific 300-feature sets, and final Elastic Net hyperparameters were applied once to the locked 2D holdout of 171 models representing 165 patient groups. Panel-wide, paired-contrast, target-level, and deployment-finalization results are shown in Figure 3a–d; detailed numerical results are provided in Table S11.

As shown in Figure 3a,b, the development OOF macro target-wise Spearman correlation was 0.4071 (95% CI, 0.3893–0.4232), and the locked-holdout value was 0.4094 (95% CI, 0.3730–0.4383). The holdout-minus-development difference was +0.0023 (95% CI, −0.0372 to 0.0361). The interval included zero, indicating no detectable difference within this preselected panel. This comparison does not remove the dependence introduced by historical target selection.

The lineage-only holdout reference yielded a macro correlation of 0.2969 (95% CI, 0.2529–0.3368). The expression-only-minus-lineage-only difference was +0.1125 (95% CI, 0.0765–0.1472). The patient-aggregated macro correlation was 0.4133, and the within-lineage permutation analysis across 165 patient groups yielded *p* = 0.00050. These prespecified checks supported rank information beyond lineage composition within the preselected panel. They do not establish independence from historical target selection.

All 20 locked-holdout target correlations were positive and ranged from 0.170 to 0.550 (Figure 3c; Table S11). The highest values were observed for *EP300* (ρ = 0.550), *CDK6* (ρ = 0.532), and *TSC2* (ρ = 0.525), whereas the lowest were observed for *RAF1* (ρ = 0.170), *MAPK1* (ρ = 0.197), and *BRAF* (ρ = 0.341). Absolute error, R^2^, and calibration measures remained secondary diagnostics and did not alter the rank-based primary assessment.

Following the one-time holdout evaluation, the selected model was refitted on all 852 2D models while the representation, 20-target panel, 300 target-specific feature identities, hyperparameters, and primary metric remained fixed (Figure 3d). All 20 deployment models converged. The 6000 target-feature assignments represented 3845 unique expression features. The final lock, strict audit, and independent replay passed 18/18, 41/41, and 50/50 checks, respectively, with a maximum prediction-reproduction difference of 0.0 (Table S11).

Because the historical target panel had been selected using all 852 2D models, these holdout results provide internal confirmation conditional on that selection. They do not provide an unbiased estimate of generalization for the complete analysis pipeline. The independent 3D validation assessed transfer to new organoid samples only for the same preselected panel.

### 3.3. Exploratory Transfer of the Locked Model to Broad-94 Organoids

**Figure 4:**
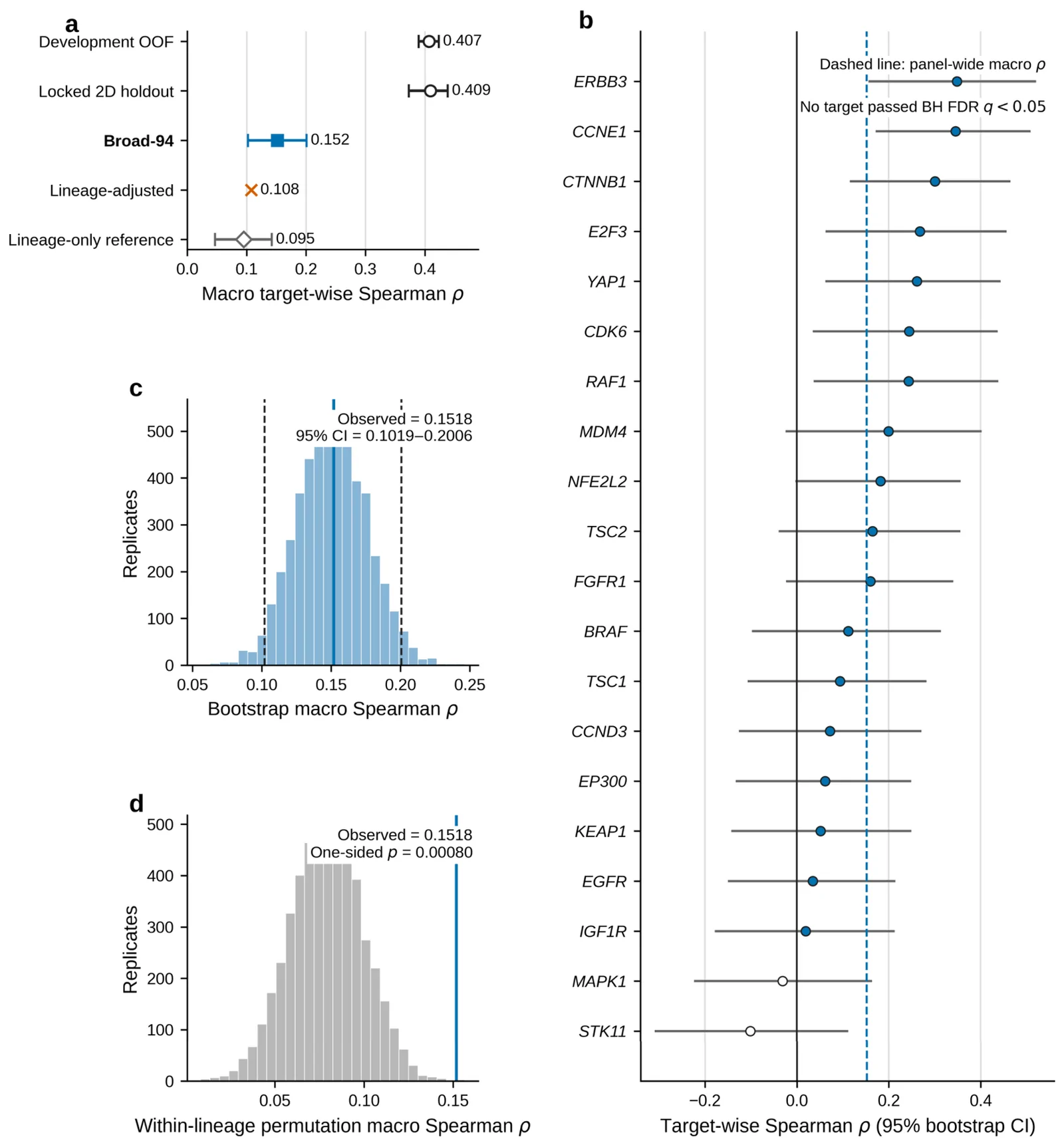
Exploratory transfer of the locked expression-only model to Broad-94 organoids. (**a**) Panel-wide, macro target-wise Spearman correlations for development OOF predictions, the locked two-dimensional holdout, the Broad-94 expression-only model, the lineage-adjusted Broad estimate, and the Broad lineage-only reference. Error bars show 95% bootstrap confidence intervals where available; the lineage-adjusted estimate is shown without an interval. (**b**) Target-wise Spearman correlations with 95% lineage-stratified bootstrap confidence intervals, ordered by point estimate; the dashed line denotes the Broad-94 panel-wide macro correlation. (**c**) Lineage-stratified bootstrap distribution of the Broad-94 macro Spearman correlation; dashed lines denote the 95% confidence interval. (**d**) Within-lineage permutation null distribution; the vertical line denotes the observed macro correlation. The original inference used 94 organoid models because donor identifiers were absent from the original evaluation records. These intervals and permutation results assume model-level independence; later partial-donor sensitivities are reported in Tables S22–S24. All 20 target-specific feature sets had 100% coverage. Eighteen target estimates were positive, but no target passed Benjamini–Hochberg false discovery rate correction at q < 0.05. Broad-94 remained exploratory and was not treated as independent external validation. In (**a**), open circles denote the development and holdout estimates, the blue square denotes Broad-94, the orange cross denotes the lineage-adjusted estimate, and the open diamond denotes the lineage-only reference. In (**b**), filled blue circles denote positive correlations, whereas open circles denote non-positive correlations; the solid black line marks zero correlation. Blue solid lines in (**c**,**d**) mark the observed macro correlation. Blue histogram bars in (**c**) show bootstrap replicates, and gray histogram bars in (**d**) show permutation-null replicates. 2D, two-dimensional; BH, Benjamini–Hochberg; CI, confidence interval; FDR, false discovery rate; OOF, out-of-fold.

The finalized expression-only deployment model was applied to 94 Broad NextGen organoids without retraining, cohort-specific feature selection, or recalibration. All 300 locked expression features were available for every target, yielding 100% feature coverage. The 94 × 20 prediction matrix was written and SHA-256-sealed before alignment with the observed 3D Gene Effect outcomes. Because Broad-94 had been accessible in earlier methodological work, this analysis remained an exploratory 3D transfer assessment rather than an independent external validation. Panel-wide, target-level, bootstrap, and permutation results are shown in Figure 4a–d; full results and lineage sensitivity analyses are provided in Table S12.

As shown in Figure 4a,c,d, the macro target-wise Spearman correlation in Broad-94 was 0.1518 (95% lineage-stratified bootstrap CI, 0.1019–0.2006). Eighteen of 20 target correlations were positive, and the median target correlation was 0.1628. The Broad-94 macro correlation was 0.2576 lower than the locked 2D holdout value. In the within-lineage permutation analysis, an association at least as large as the observed value occurred rarely (one-sided *p* = 0.00080), supporting partial but attenuated transfer of rank information.

The lineage-adjusted macro Spearman correlation was 0.1080, whereas the lineage-only reference yielded 0.0951. The paired expression-only-minus-lineage-only difference was +0.0567 (95% CI, 0.0040–0.1106). Thus, lineage composition did not fully explain the exploratory association, although it accounted for part of the observed signal.

At the target level, the highest correlations were observed for *ERBB3* (ρ = 0.3489), *CCNE1* (ρ = 0.3456), *CTNNB1* (ρ = 0.3009), *E2F3* (ρ = 0.2679), and *YAP1* (ρ = 0.2614) (Figure 4b; Table S12). *MAPK1* (ρ = −0.0307) and *STK11* (ρ = −0.1010) were the only targets with negative correlations. After adjustment of the 20 target-level permutation *p* values, no target met the Benjamini–Hochberg FDR threshold of *q* < 0.05; the lowest adjusted value was *q* = 0.0640 for *CCNE1*. Accordingly, Broad-94 was interpreted at the fixed-panel level rather than as confirmatory evidence for individual targets.

Among lineage groups containing at least seven models, macro correlations ranged from −0.0099 to 0.2786. Leave-one-lineage-out estimates ranged from 0.1202 to 0.1832, and the pooled association remained positive after every lineage exclusion. The original estimates used model-level resampling. The subsequent donor audit and sensitivity analyses below address the identified shared donor, while retaining uncertainty about unresolved donor relationships.

The donor audit identified one confirmed same-donor pair among 25 models with donor information; 69 models remained unresolved (Table S22). Omitting ACH-002883 or ACH-002884 gave macro target-wise correlations of 0.1516 and 0.1539, respectively, compared with the original 0.1518. Under the conditional full-cohort bootstrap, the expression-only estimate was 0.1518 (0.1009 to 0.2007) and its paired difference from lineage-only prediction was 0.0567 (0.0027 to 0.1081) (estimate and 95% CI; Table S23). These findings quantify sensitivity to the identified pair, conditional on treating the unresolved models as separate units.

In the 25-model donor-documented subset, the expression-only macro correlation was 0.0741 (−0.0154 to 0.1618), with an expression-minus-lineage difference of −0.0571 (−0.1502 to 0.0305) under source-specific donor-cluster resampling. Both intervals included zero, so this subset did not establish superiority over lineage. Averaging the vectors within the 24 source-specific donor groups gave 0.0727 (−0.0195 to 0.1629) for expression-only prediction. This subset is not a representative replacement for Broad-94; changes relative to the full cohort can reflect sample and lineage composition as well as grouping. Paired within-subset model-versus-cluster intervals and secondary ranking sensitivities are provided in Tables S23 and S24. Incomplete donor ascertainment prevents these analyses from verifying full-cohort independence or providing a fully donor-adjusted effective sample size.

### 3.4. Blinded Independent External Validation in Sanger-148 Organoids

**Figure 5:**
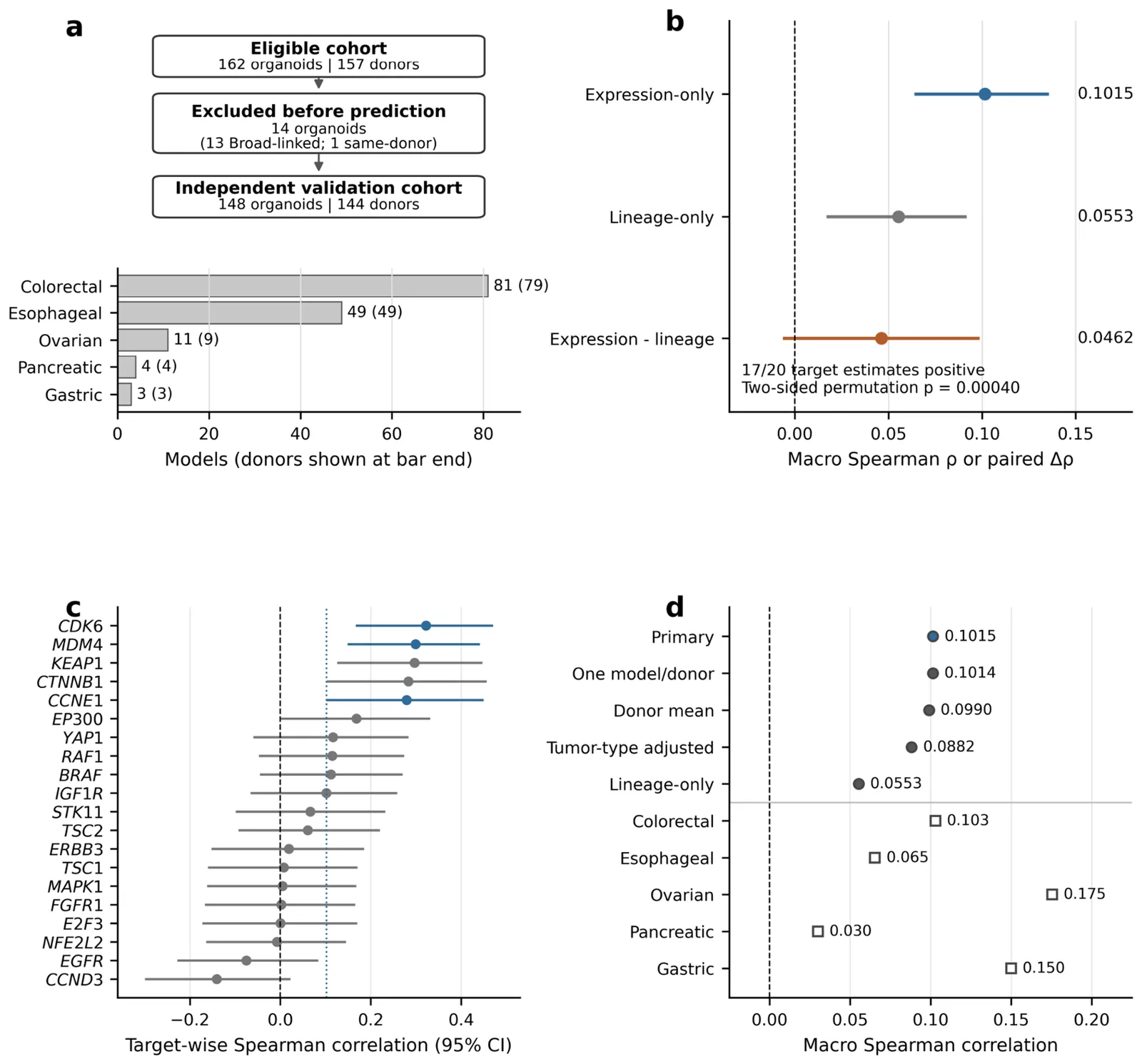
Blinded independent external validation in Sanger-148 organoids. (**a**) Cohort construction and tumor-type composition. Fourteen organoids were excluded from 162 eligible organoids representing 157 donors, leaving 148 organoids from 144 donors. (**b**) Panel-wide expression-only, lineage-only, and paired-difference estimates; error bars show 95% donor-cluster bootstrap confidence intervals. (**c**) Target-specific Spearman correlations with 95% confidence intervals. Blue points denote Benjamini–Hochberg q < 0.05; the dotted line denotes the macro correlation. (**d**) Robustness and tumor-type subgroup estimates. Predictions were SHA-256-sealed before outcome access; the primary macro correlation was 0.1015, 17/20 target estimates were positive, and the two-sided within-tumor-type donor-level permutation *p*-value was 0.00040. In (**a**), arrows indicate cohort selection. In (**b**), blue, gray, and orange points and error bars denote expression-only, lineage-only, and expression-minus-lineage estimates, respectively. In (**c**), gray points denote q ≥ 0.05, and error bars use the corresponding point colors. In (**d**), filled circles denote cohort-level estimates, with the primary estimate in blue and the sensitivity and lineage-only reference estimates in gray; open squares denote tumor-type subgroup estimates. Black dashed vertical lines in (**b**–**d**) mark zero. BH, Benjamini–Hochberg; CI, confidence interval; SHA-256, 256-bit Secure Hash Algorithm.

The outcome-blinded Sanger eligibility pool comprised 162 organoids from 157 donors with both RNA-seq profiles and quality-controlled CRISPR screening records. After excluding 13 Broad-linked organoids and one additional organoid from the same donor as a Broad-linked model, the independent validation cohort contained 148 organoids from 144 unique donors. Of the 3845 unique locked expression features used across the 20 target models, 3790 were measured in the Sanger matrix; target-specific coverage ranged from 291 to 300 of 300 features (97.0–100%). The 148 × 20 blind prediction matrix and the associated manifests were SHA-256 sealed before CRISPR LFC outcomes were accessed, and all 11 prespecified pre-outcome seal checks passed. Panel-wide validation, target-level results, and robustness analyses are summarized in Figure 5 and Table S13.

As shown in Figure 5a,b, the frozen expression-only model achieved a macro target-wise Spearman correlation of 0.1015 in the independent Sanger-148 cohort (95% CI, 0.0637–0.1357). Seventeen of the 20 target estimates were positive. After repeated organoids were averaged within donors, 5000 two-sided permutations of complete 20-target outcome vectors within tumor-type strata yielded *p* = 0.00040. Thus, the locked 2D model carried a detectable rank-ordering signal into the independent 3D cohort, although the effect size was modest.

As shown in Figure 5c and Table S13, the largest target-specific correlations were observed for *CDK6* (ρ = 0.3221; 95% CI, 0.1666–0.4702), *MDM4* (ρ = 0.2992; 95% CI, 0.1483–0.4410), *KEAP1* (ρ = 0.2967; 95% CI, 0.1256–0.4464), *CTNNB1* (ρ = 0.2831; 95% CI, 0.1031–0.4563), and *CCNE1* (ρ = 0.2792; 95% CI, 0.1014–0.4493). After Benjamini–Hochberg correction of the 20 target-level permutation *p*-values, only *CDK6*, *MDM4*, and *CCNE1* met q < 0.05 (q = 0.0040 for each). *CCND3*, *EGFR*, and *NFE2L2* were the three targets with negative correlations; no targets were removed based on direction or significance.

The tumor-type-adjusted macro Spearman correlation was 0.0882, whereas the frozen lineage-only reference achieved 0.0553 (95% CI, 0.0168–0.0917). The expression-only-minus-lineage difference was +0.0462, but its paired donor-cluster bootstrap interval included zero (95% CI, −0.0065 to 0.0986). The primary target-wise endpoint therefore did not establish superiority beyond tumor-origin information. The one-model-per-donor estimate (ρ = 0.1014) and donor-mean estimate (ρ = 0.0990) closely matched the primary result. Tumor-type subgroup estimates were 0.1030 for colorectal, 0.0654 for esophageal, 0.1755 for ovarian, 0.0300 for pancreatic, and 0.1500 for gastric organoids (Figure 5d). Ovarian results were exploratory, whereas pancreatic and gastric estimates were descriptive because of their small sample sizes.

An independent replay reproduced the cohort composition, target and prediction order, feature coverage, primary and sensitivity statistics, lock records, and file hashes, with all 22 predefined checks passing. No model retraining, target or feature selection, hyperparameter adjustment, or change to the prespecified primary metric occurred after the Sanger outcomes were opened. The retrospective within-organoid endpoints are reported separately in Section 3.7. These findings support a statistically detectable but quantitatively modest independent 3D rank-transfer signal, with the strongest target-level support for *CDK6*, *MDM4*, and *CCNE1*.

### 3.5. Retrospective Cross-Cohort Transportability Synthesis

**Figure 6:**
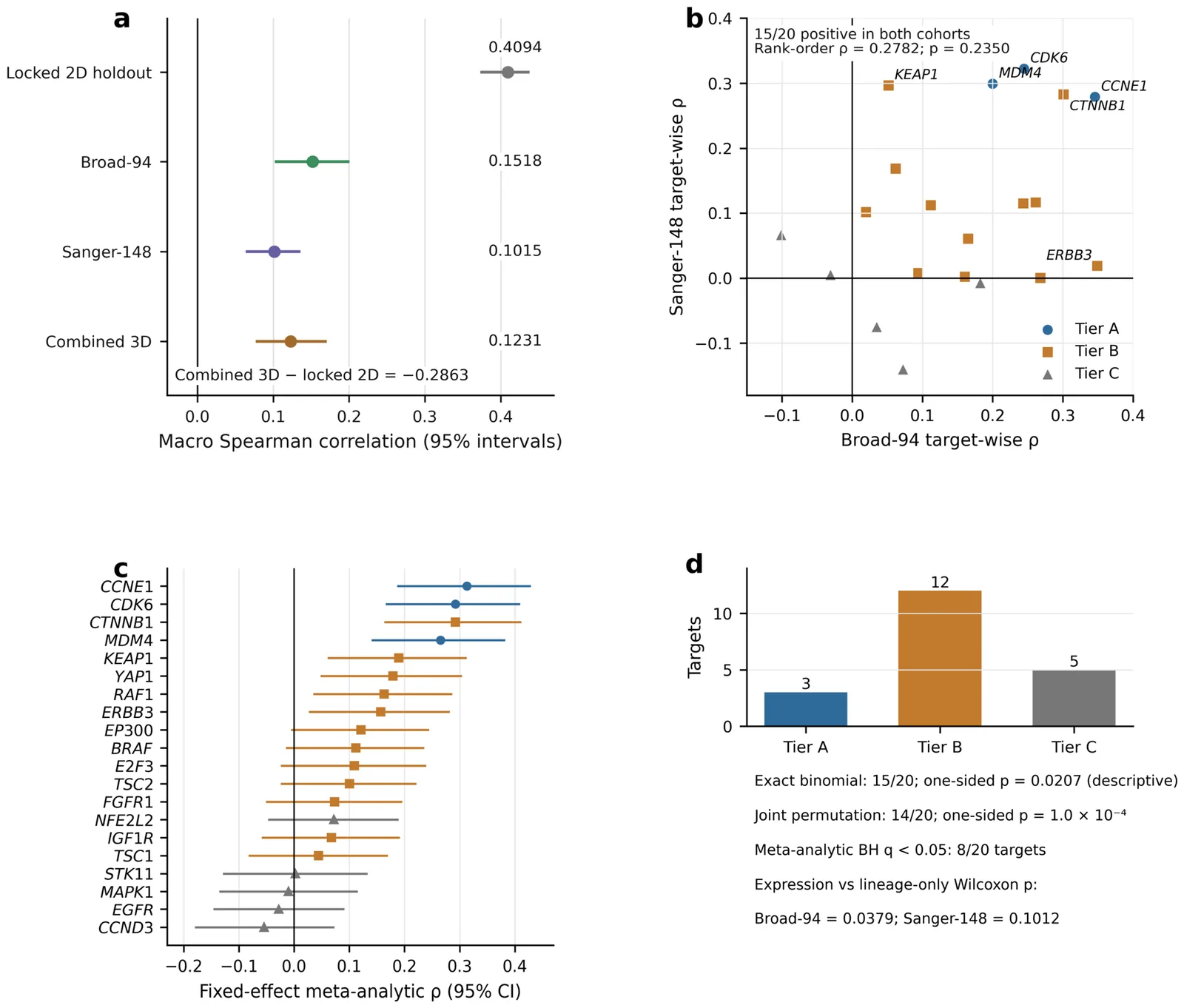
Retrospective cross-cohort transportability synthesis across Broad-94 and Sanger-148 organoids. (**a**) Macro correlations for the locked 2D holdout, Broad-94, Sanger-148, and the fixed-effect combined 3D estimate. Error bars show cohort-specific 95% bootstrap confidence intervals and, for the combined 3D estimate, a descriptive 95% target-panel bootstrap interval. (**b**) Target-wise Broad-94 versus Sanger-148 correlations, classified as Tier A, B, or C. (**c**) Fixed-effect target-level meta-analytic correlations with 95% confidence intervals. (**d**) Evidence-tier counts and sensitivity summaries. The exact-binomial benchmark was descriptive, and the 50,000-iteration correlation-aware permutation was post hoc; neither constituted a new external validation. In (**a**), gray, green, purple, and brown points and error bars denote the locked 2D holdout, Broad-94, Sanger-148, and combined 3D estimates, respectively. In (**b**,**c**), blue circles, orange squares, and gray triangles denote Tiers A, B, and C, respectively; the same tier colors are used for the bars in (**d**). Solid black reference lines in (**a**–**c**) mark zero correlation. 2D, two-dimensional; 3D, three-dimensional; BH, Benjamini–Hochberg; CI, confidence interval.

After the Broad-94 exploratory analysis and the Sanger-148 blinded external validation had been completed separately, their target-specific results were retrospectively combined for the unchanged 20-gene panel. The synthesis evaluated the direction and magnitude of rank transfer rather than the absolute agreement between the Broad Chronos Gene Effect and Sanger-corrected CRISPR LFC scales. Panel-wide and target-level synthesis results are summarized in Figure 6 and Table S14.

As shown in Figure 6a, the arithmetic mean of the 20 fixed-effect target-level meta-analytic correlations was 0.1231, with a 20,000-replicate target-panel bootstrap interval of 0.0769–0.1704. This combined 3D estimate was substantially lower than the locked 2D holdout correlation of 0.4094; the combined 3D-minus-locked-2D difference was −0.2863. The results therefore indicated measurable transfer of dependency ranking into organoids, accompanied by marked attenuation relative to performance within the 2D domain. This observed cross-domain performance contrast does not identify the biological or technical causes of attenuation.

Fifteen of the 20 frozen targets had positive correlations in both Broad-94 and Sanger-148 (75%). A one-sided exact binomial comparison against a null success probability of 0.50 yielded *p* = 0.0207. The targets were correlated, and we defined the synthesis after the cohort-specific results were known. We therefore interpreted this value only as a descriptive benchmark. In the post hoc correlation-aware sensitivity analysis, Sanger outcomes were first averaged within donors, and each unit’s complete 20-target outcome vector was permuted as a block. Fourteen of 20 targets remained positive in both cohorts, and 50,000 simultaneous permutations yielded a one-sided empirical *p* = 1.0 × 10^−4^ (Figure 6d).

As shown in Figure 6b,c and Table S14, eight targets met the combined meta-analytic Benjamini–Hochberg threshold of q < 0.05. The reporting classification comprised three Tier A, 12 Tier B, and five Tier C targets. The strongest Tier A support was observed for *CCNE1* (Broad ρ = 0.3456; Sanger ρ = 0.2792; combined ρ = 0.3128; 95% CI, 0.1865–0.4290; q = 5.18 × 10^−5^), *CDK6* (0.2447; 0.3221; combined ρ = 0.2924; 95% CI, 0.1660–0.4094; q = 1.01 × 10^−4^), and *MDM4* (0.2000; 0.2992; combined ρ = 0.2655; 95% CI, 0.1400–0.3825; q = 2.36 × 10^−4^). These targets combined positive transfer in both cohorts, multiplicity-adjusted meta-analytic support, and target-level FDR support in at least one individual cohort.

The correlation between the vectors of target-specific Broad and Sanger performance estimates was ρ = 0.2782 (two-sided *p* = 0.2350), indicating that panel-wide directional replication did not imply stable relative performance ordering across cohorts (Figure 6b). The expression-only-minus-lineage differences were positive at the panel level in Broad-94 (one-sided Wilcoxon *p* = 0.0379) but did not reach the corresponding threshold in Sanger-148 (*p* = 0.1012). Thus, the retrospective synthesis strengthened evidence for limited cross-cohort directionality but did not constitute a third external validation or support equivalent target performance across the two organoid resources.

### 3.6. Secondary Transcriptomic Projection and Domain Diagnostics in Tiriac Pancreatic Organoids

**Figure 7:**
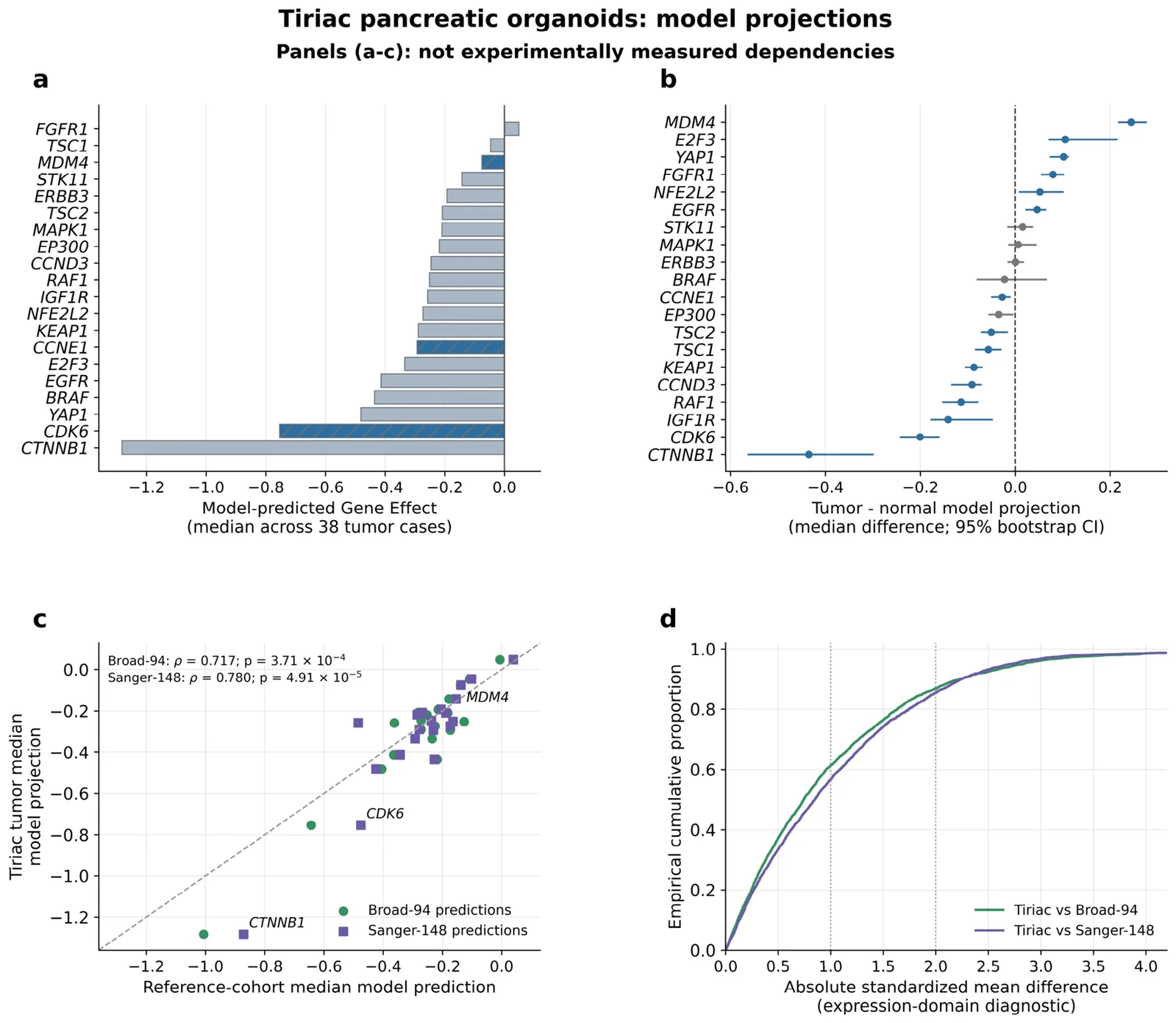
Model projections and expression-domain diagnostics in Tiriac pancreatic organoids. Panels (**a**–**c**) show model-predicted dependency scores, not experimentally measured dependencies. (**a**) Median projected Gene Effect across 38 tumor cases; blue hatched bars highlight *CDK6*, *CCNE1*, and *MDM4*, whereas gray bars represent the other targets. More negative values indicate stronger projected dependency within each model. (**b**) Tumor-minus-normal projection differences with 95% case-bootstrap confidence intervals; blue points and error bars indicate BH q < 0.05, whereas gray points and error bars indicate BH q ≥ 0.05; the vertical dashed line marks zero difference. (**c**) Tiriac tumor-median projections versus Broad-94 and Sanger-148 median predictions; the dashed line denotes numerical equality, not agreement with measured outcomes. (**d**) Expression-domain diagnostics: cumulative distributions of absolute SMD across 3790 shared features; dotted lines mark |SMD| = 1 and 2. Tiriac lacked measured CRISPR dependencies; these projections require experimental validation. BH, Benjamini–Hochberg; CI, confidence interval; CRISPR, clustered regularly interspaced short palindromic repeats; SMD, standardized mean difference.

After completion of 2D model development, Broad-94 exploration, Sanger-148 validation, and retrospective synthesis, the same frozen expression-only deployment models were applied to the independent Tiriac pancreatic organoid RNA-seq cohort for secondary biological projection. The analysis included 55 samples: 44 tumor samples representing 38 independent tumor cases and 11 normal samples representing 11 independent normal cases. Of 3845 unique locked expression features, 3806 were observed (98.99%); target-specific coverage ranged from 292 to 300 of 300 features (97.33–100%), and all targets passed the prespecified 80% coverage gate. Because no measured genome-scale CRISPR dependency outcomes were available, these analyses evaluated model-output distributions and expression-domain shift rather than predictive accuracy. Figure 7 and Table S15 present model projections, not experimentally measured dependencies, together with expression-domain diagnostics.

As shown in Figure 7a, the most negative median projection among the 38 tumor cases was observed for *CTNNB1* (−1.282), followed by *CDK6* (−0.753), *YAP1* (−0.481), *BRAF* (−0.435), and *EGFR* (−0.414). *CTNNB1* and *CDK6* ranked among the three most negative within-case projections in 37 of 38 tumor cases. Because each target was modeled separately, these values represent target-specific projected outputs rather than a common calibrated dependency scale.

Fifteen of the 20 tumor-normal model-output contrasts met Benjamini–Hochberg q < 0.05 (Figure 7b and Table S15). The largest tumor-directed difference was observed for *CTNNB1* (tumor minus normal, −0.435; 95% bootstrap CI, −0.564 to −0.298; q = 0.00439). *CDK6* also showed a more negative tumor projection (−0.200; 95% CI, −0.243 to −0.159; q = 2.09 × 10^−5^), and *CCNE1* showed a smaller difference in the same direction (−0.027; 95% CI, −0.051 to −0.009; q = 0.0260). By contrast, *MDM4* was more negative in normal cases (tumor minus normal, +0.245; 95% CI, +0.217 to +0.277; q = 1.17 × 10^−5^). These *p* and q values describe differences in frozen model outputs and do not demonstrate measured gene dependency or therapeutic sensitivity.

The 20-target median projection ranking in Tiriac tumor cases was concordant with the Broad-94 median prediction ranking (ρ = 0.717; two-sided *p* = 3.71 × 10^−4^) and the Sanger-148 median prediction ranking (ρ = 0.780; *p* = 4.91 × 10^−5^; Figure 7c). All three vectors were predictions from the same frozen model family, and Tiriac lacked measured CRISPR outcomes. These correlations therefore quantify target-hierarchy consistency rather than independent predictive accuracy.

Expression-domain diagnostics demonstrated substantial cohort shift. Across the 3790 frozen features shared by the three organoid resources, the median absolute SMD was 0.743 for Tiriac versus Broad-94 and 0.848 for Tiriac versus Sanger-148. The proportions with |SMD| > 1 were 38.7% and 43.4%, respectively, and the proportions with |SMD| > 2 were 13.2% and 14.6% (Figure 7d). PCA likewise separated the Tiriac samples from the Broad and Sanger cohorts. We reported these diagnostics without recalibration, feature deletion, target selection, or prediction modification. Accordingly, the Tiriac findings support secondary application and prioritization only, and we did not interpret them as validated dependencies.

Computational replay confirmed that the reference Tiriac projections used training-median imputation. Relative to these unchanged projections, zero filling produced a mean absolute change of 0.0114 and a maximum change of 0.1077 Gene Effect units; training-reference mean filling produced corresponding changes of 0.0017 and 0.0172. Across the 38 tumor-case profiles, mean within-profile Spearman agreement with the reference was 0.9771 for zero filling and 0.9991 for training-reference means. The top-three set was preserved in 36/38 and 38/38 tumor cases, respectively (Table S20). Zero filling therefore altered the short candidate list in two tumor cases despite high overall rank agreement.

The largest zero-fill shift occurred for *RAF1* (−0.1077), whereas the largest training-reference mean shift occurred for *CTNNB1* (−0.0172; Table S21). The tumor-median top-three hierarchy remained *CTNNB1*, *CDK6* and *YAP1* under both alternatives. Tumor–normal median differences and bootstrap limits changed by less than 0.000001; Mann–Whitney *p*-values, adjusted q-values, and the 15 significant projection contrasts were unchanged. This invariance follows from the common target-specific offsets in the linear models and does not independently validate the projections. The sensitivity analysis does not identify the most accurate imputation method because Tiriac lacks measured dependency outcomes.

The retrospective coverage-threshold check retained all 20 target models in each external cohort at 80%, 90%, and 95% (Table S16). Coverage was 100% in Broad-94, 97.0–100% in Sanger-148, and 97.33–100% in Tiriac. Because the threshold acts only as an eligibility gate and none of the models failed the stricter requirements, using 90% or 95% would leave the frozen predictions and their reported summaries unchanged.

### 3.7. Retrospective Gene Ranking Within Individual Organoids

Using the unchanged 20-gene predictions, the mean within-organoid Spearman correlation was 0.3428 (95% CI, 0.3026 to 0.3826) in Broad-94 and 0.4294 (95% CI, 0.3926 to 0.4645) in Sanger-148 (Table S17). The corresponding fixed 2D-mean comparator estimates were 0.2057 and 0.1995. Paired expression-minus-fixed-mean differences were +0.1371 (95% CI, 0.0987 to 0.1780) and +0.2299 (95% CI, 0.1981 to 0.2613), respectively. These estimates address gene ordering within each organoid and are not directly comparable to the original macro target-wise correlations of 0.1518 and 0.1015.

The archived lineage-only predictions yielded mean within-organoid correlations of 0.2745 in Broad and 0.3884 in Sanger. Expression-minus-lineage differences were +0.0684 (95% CI, 0.0334 to 0.1032) and +0.0411 (95% CI, 0.0167 to 0.0656), respectively. Thus, full-panel gene ranking showed a modest retrospective gain over lineage-only prediction in both cohorts (Table S17). This finding does not change the uncertainty in the original Sanger target-wise expression-minus-lineage difference.

Top-three overlap was 34.04% in Broad and 42.79% in Sanger, compared with 34.75% and 35.36% for lineage-only prediction (Table S18). Paired expression-minus-lineage differences were −0.71 percentage points (95% CI, −4.96 to 3.19) in Broad and +7.43 percentage points (95% CI, 4.28 to 10.74) in Sanger. The Broad interval included zero; superiority for selecting the three strongest measured dependencies was therefore not demonstrated across both cohorts.

Averaging the 20-gene vectors within each Sanger donor before evaluation gave a mean within-donor correlation of 0.4338 (95% CI, 0.3986 to 0.4675). The paired difference from lineage-only prediction was +0.0402 (95% CI, 0.0157 to 0.0652). Mean top-three overlap was 42.82%, with an expression-minus-lineage difference of +6.94 percentage points (95% CI, 4.17 to 9.95; Table S19). These results were consistent with the organoid-weighted analysis. All within-organoid findings remain retrospective and restricted to the selected 20-gene panel.

## 4. Discussion

This study tested whether transcriptome-based gene-dependency rankings learned in 2D cancer models retained a detectable but attenuated signal in patient-derived 3D organoids. Transfer was evaluated without outcome-guided retraining. The findings provide limited support for this hypothesis. We selected the expression-only representation in the development cohort. Holdout performance provided internal confirmation conditional on historical target selection. Positive panel-wide rank signals were observed in the Broad-94 exploratory cohort and the Sanger-148 blinded independent external validation cohort. However, macro correlations in Broad, Sanger, and the combined 3D analysis were markedly lower than in the locked 2D holdout. Transfer was therefore incomplete and weak. The findings support modest, context-sensitive preservation of organoid ranking for genes within the preselected panel across independently generated resources. They do not establish cross-platform equivalence. This primary finding is distinct from the secondary within-organoid gene-ranking results in Section 3.7.

Tumor lineage is central to interpreting this transfer. Expression patterns can encode tissue identity and shared transcriptional programs. A model may therefore reproduce differences in dependency between tumor types without identifying the mechanisms responsible for those dependencies. The CRISPR outcomes measure gene-depletion effects, but the fitted expression–dependency relationships remain predictive associations. Their transfer does not establish that the predictive expression features have a causal role in determining dependency. Thus, predictive generalization to organoids and mechanistic generalization are distinct claims.

For the prespecified target-wise endpoint, the added predictive value of transcriptomic modeling over tumor lineage was not established in the blinded Sanger cohort. The expression-only model exceeded the frozen lineage-only comparator numerically by 0.0462 in macro Spearman correlation. However, the paired donor-cluster bootstrap CI included zero (95% CI, −0.0065 to 0.0986). This result establishes neither superiority nor equivalence. The significant within-lineage permutation result addresses a different question: whether the prediction–outcome association exceeds a null distribution that preserves tumor-type structure. It does not establish superior performance over the lineage-only predictor. Target-specific correlations rank organoids for a given gene. They cannot by themselves establish correct gene ranking within an organoid or an increase in the yield of functional experiments. The study contributes an outcome-blinded assessment of performance attenuation from cell lines to organoids and documents uncertainty in the incremental value of transcriptomic prediction. *CDK6*, *MDM4*, and *CCNE1* remain candidates for prospective investigation. Their multiplicity-adjusted correlations do not establish target-specific superiority over lineage or practical benefit.

The retrospective within-organoid analysis directly examined this second ranking task. Full 20-gene rankings improved over a fixed 2D-mean ranking and, more modestly, over lineage-only prediction in both cohorts (Table S17). These comparisons are needed because shared differences in average dependency among genes can produce positive within-organoid correlations. A gain in full-panel correlation did not ensure a better short candidate list. Top-three overlap exceeded lineage-only prediction in Sanger but not in Broad (Table S18). The donor-mean sensitivity analysis gave similar Sanger results (Table S19). This is limited retrospective evidence of ranking agreement within a selected panel, not prospective proof that transcriptomic modeling improves experimental yield. It neither replaces the original primary analysis nor resolves its uncertainty about target-wise superiority over lineage.

Adding copy-number, mutation, and lineage information to gene expression did not reliably improve development performance. DeepDEP and transcriptome-based maps developed for The Cancer Genome Atlas use molecular profiles from cancer cell lines to estimate dependencies in functionally unscreened tumors [16,17]. Synthetic data augmentation and multi-omic integration have also been proposed to address incomplete profiles and capture more complex dependency patterns [18]. The expression-plus-copy-number candidate yielded the highest development score numerically. However, its advantage over expression-only was negligible, and the paired bootstrap interval included zero. The expression-only representation also showed the greatest feature-set stability and was selected by the prespecified one-standard-error rule. This result does not imply that copy number or mutation is biologically uninformative. This finding applies to the fixed 20-target panel, the available sample size, the 300-feature limit per target, and the linear Elastic Net framework. Within these constraints, the additional data layers did not provide a sufficiently stable incremental signal to justify greater complexity. The broad availability and relatively consistent measurement of transcriptomic data across model collections further strengthens the practical rationale for an expression-only deployment model.

Performance was lower in the organoid cohorts than in the 2D internal confirmation analysis. The macro Spearman correlation was 0.4094 in the locked 2D holdout, compared with 0.1518 in Broad-94 and 0.1015 in Sanger-148. This attenuation is interpreted as an observed cross-domain effect whose biological and technical causes remain uncertain. Computational alignment studies have documented transcriptional differences between cell lines and tumors [20]. Next-generation model resources have reported tumor-relevant transcriptional programs and culture-specific dependencies in organoids and spheroids [38], while patient-derived model collections have shown that culture conditions can modify cellular states in a subset of models [40]. These studies provide context for plausible explanations, but they do not establish the causes of the performance decline in the present cohorts. Differences in cellular state, growth geometry, medium composition, lineage composition, and functional-screening platforms may contribute individually or jointly. Their contributions were not experimentally disentangled or quantified here. The comparison used different cohorts and assay settings, rather than paired models in which growth conditions and screening platforms were varied under controlled conditions. It therefore cannot attribute the decline to 3D growth itself, identify a dominant mechanism, or exclude contributions from model limitations and cohort composition. Historical target-panel selection also limits interpretation of the 2D reference estimate. The observed attenuation supports evaluating dependency models across data domains without assigning a confirmed biological or technical explanation to the decline.

The contrasting roles of Broad-94 and Sanger-148 were central to the evidential interpretation. Broad-94 was retained as exploratory because the resource had been accessible during earlier methodological work and donor information was absent from the original evaluation records. The later audit recovered only partial donor information and confirmed a same-donor pair (Tables S22–S24); it did not establish independence of the full cohort. The audited analysis run nevertheless preserved a prediction-before-outcome sequence. Sanger-148, by contrast, was constructed after excluding Broad-linked and same-donor records, and its predictions were cryptographically sealed before the CRISPR outcomes were opened. Donor-clustered uncertainty estimation and donor-aware permutation further preserved the strongest available biological independence structure. Differences in screening libraries, protocols, and computational processing can affect Broad and Sanger dependency estimates [10,11]. Chronos was developed in part to model sources of variation in CRISPR screen dynamics [12]. Target-wise correlations were positive for 18 of 20 targets in Broad-94 and 17 of 20 in Sanger-148. This pattern suggests that the panel-wide direction was not confined to a single laboratory or processing system. Nevertheless, the Sanger target-wise expression-only-minus-lineage CI included zero, so that cohort did not establish an incremental advantage on the prespecified primary endpoint. Transcriptomic information therefore carried a detectable external rank signal, but part of that signal may reflect lineage-associated structure.

The target-level findings provided the strongest cross-cohort support for *CCNE1*, *CDK6*, and *MDM4*. *CCNE1* was positive in both organoid cohorts, passed the FDR threshold in Sanger-148, and yielded one of the largest combined estimates. This is biologically consistent with the recent Sanger organoid dependency map, in which *CCNE1* amplification in esophageal organoids was associated with *CCNE1* dependency [39]. *CDK6* produced the highest target-specific correlation in Sanger-148 and remained in Tier A in the retrospective synthesis. The Broad NextGen study reported enrichment for *CDK6* dependency in glioblastoma models that preserve a tumor-proximal glial transcriptional state. Genetic and pharmacological experiments supported this association in specific genomic contexts [38]. That work provides an example of how transcriptional state can expose context-dependent *CDK6* vulnerability. *MDM4* also showed a positive ranking signal in both cohorts. However, the current reference set provides less direct organoid-based mechanistic support for *MDM4* than for *CCNE1* or *CDK6*. *MDM4* should therefore be regarded primarily as a hypothesis for targeted functional testing. These findings identify genes whose variation across organoids was predicted with the strongest support. They do not establish that these are the three strongest dependencies within an individual organoid. More generally, their target-specific correlations do not demonstrate drug sensitivity, therapeutic index, or clinical benefit.

The retrospective synthesis distinguished directional replication at the panel level from stability in the relative ordering of target performance. Fifteen of the 20 targets were positive in both cohorts. The directionally concordant count remained above the correlation-aware null expectation when complete target vectors were permuted as blocks. In contrast, the correlation between the Broad and Sanger vectors of target-specific performance was only 0.2782 and was not statistically significant. Most targets therefore transferred in the same direction. This does not imply that they were predicted with equivalent strength in both organoid resources. The Tier A–C framework summarized these differences. Tier A identified the three targets with replicated direction, combined multiplicity-adjusted support, and individual-cohort evidence. Tier B captured directionally concordant but less strongly supported signals. Tier C indicated non-positive transfer in at least one cohort. Because these tiers were assigned retrospectively after the cohort-specific results were known, they should be treated as reporting categories rather than validated biological classes. Their reproducibility must be tested prospectively in a new cohort using prespecified criteria.

The Tiriac pancreatic organoid analysis provided a complementary application layer rather than an additional validation experiment. Pancreatic organoids have been shown to preserve important disease features and treatment-response patterns [30,31,32,33]. The Tiriac tumor-case target ranking was concordant with the median prediction rankings in Broad and Sanger. Thus, the frozen model family produced a broadly consistent target hierarchy in an independent pancreatic transcriptomic resource. However, all compared values were predictions from the same model family, and Tiriac provided no measured genome-scale CRISPR outcomes. The observed concordance therefore reflects cross-cohort consistency of model outputs, not predictive accuracy. Moreover, principal component separation and large SMDs across the shared expression features indicated substantial domain shift. *CTNNB1* had the most negative pancreatic projection but was not a Tier A target in the CRISPR synthesis. *MDM4* was projected to be more negative in normal than tumor cases. These findings further illustrate why the outputs should not be interpreted as a context-independent hierarchy of biological dependency.

The retrospective missing-feature sensitivity analysis showed high overall agreement with the original median-imputed Tiriac projections. However, zero filling changed the top-three set in two of 38 tumor cases, and individual target shifts reached 0.1077 Gene Effect units. Training-reference mean filling preserved all tumor-case top-three sets, but this agreement cannot establish greater accuracy. Constant per-target offsets explain the unchanged tumor–normal contrasts. These findings support reporting the preprocessing rule explicitly and retaining the projections as hypotheses for functional testing (Tables S20 and S21).

The computational framework may help select direct organoid experiments, but its utility requires prospective testing. Genome-scale or large-scale CRISPR studies in intestinal, colon, and gastric organoids have identified context-specific genes related to growth, treatment resistance, and gene-drug interactions [34,35,36,37]. The larger Sanger organoid biobank has likewise mapped dependencies linked to genomic and clinical features through direct perturbation [39]. Such experiments provide stronger evidence of functional causality than computational projections, but remain technically demanding and resource-intensive. Frozen expression models could be evaluated as a low-cost pre-screen for allocating scarce CRISPR or pharmacological validation capacity. The within-organoid analysis tests the relative ordering of measured scores within 20 genes. It does not establish a common calibrated dependency scale across independently fitted gene models. Its modest full-ranking gains and cohort-dependent top-three results require prospective confirmation before claiming improved target prioritization.

Several design features strengthen the study. Patient-grouped nested cross-validation reduced leakage between biologically related samples. The one-standard-error rule limited unnecessary complexity when candidate performance was nearly equivalent [22,23]. The locked 2D evaluation, the subsequent all-852 deployment refit, the Broad exploratory application, and the Sanger blinded external validation were kept analytically distinct. Targets, feature identities, hyperparameters, and the primary metric were not reopened in response to 3D outcomes. Prediction-before-outcome locks, SHA-256 manifests, independent computational replay, and bootstrap and permutation schemes matched to the available biological unit reduced opportunities for retrospective optimization. Retaining all 20 targets irrespective of effect direction or statistical significance also reduced selective reporting. The accompanying reproducibility package allows readers to trace the reported results to frozen predictions, analysis records, and integrity checks.

The study also has important limitations. First, historical selection of the fixed 20-gene panel used all 852 2D models. The 171-model holdout was independent of the current candidate representation, feature selection, hyperparameters, and model-fitting decisions. However, it was not fully independent of historical target selection. The holdout therefore provides internal confirmation conditional on that selection, not an unbiased estimate of generalization for the complete analysis pipeline. Selection-induced optimism cannot be excluded or quantified by the reported fixed-panel analysis. Second, all external performance estimates are restricted to the preselected 20 targets. Sample independence in Sanger does not establish dependency transferability for unselected genes or across the genome. Target-panel bootstrap intervals quantify sensitivity to panel composition rather than sampling uncertainty for new patients or organoids. Third, donor metadata could be recovered for only 25 of the 94 Broad models, and one same-donor pair was confirmed. The remaining 69 models and cross-source donor equivalence could not be resolved. The partial-cluster and donor-documented-subset sensitivities do not establish full-cohort independence. Original Broad CIs, permutation results and the uncertainty weights used in retrospective synthesis remain conditional on model-level independence and may overstate precision if additional dependence is present. Consequently, Broad and Sanger uncertainty estimates and effective sample sizes are not directly comparable. Fourth, Sanger-148 was dominated by colorectal and esophageal organoids. Ovarian, pancreatic, and gastric subgroups were too small to establish lineage-specific generalizability. Fifth, the differing Broad Chronos Gene Effect and Sanger-corrected LFC scales precluded direct absolute-error and calibration comparisons [10,11,12]. Sixth, target-specific linear expression-only models cannot fully represent genotype-transcriptome interactions, network structure, cell–cell interactions, or the incompletely modeled stromal and immune compartments of organoid systems [24,25,29]. Finally, the Tiriac normal group contained only 11 cases and lacked measured CRISPR dependencies, matched drug-response outcomes, and clinical endpoints. The study consequently establishes rank-transfer evidence, not prospective therapeutic utility.

Adjustment for broad tumor-type labels and comparison with a lineage-only reference cannot fully separate lineage-associated transcriptional structure from other predictive signals. The present analyses therefore do not establish the causal basis of the transferred associations. Lineage adjustment and descriptive expression-domain diagnostics do not experimentally separate the causes of performance attenuation. The present design cannot estimate the individual contributions of culture conditions, cellular state, cohort composition, and screening technology to the observed cross-domain effect.

The within-organoid endpoints introduce additional limitations. They were specified retrospectively after outcomes were available, and their CIs were not adjusted for multiple secondary comparisons. The fixed panel and frozen-model analysis do not establish genome-wide ranking performance or quantify uncertainty from retraining or selecting a different gene panel. Additional same-donor dependence remains unresolved in Broad despite the partial-donor secondary ranking sensitivities in Table S24. Finally, agreement with CRISPR score ordering is not evidence of cancer selectivity, druggability, therapeutic benefit, or increased success of prospectively chosen experiments.

Future work should test the Tier A targets using direct CRISPR perturbation in preregistered, tumor-type-balanced organoid cohorts. Gene-depletion effects should be related to target-specific inhibitor responses and, where available, to matched clinical information. Distinguishing biological from technical contributions to attenuation would require larger cohorts with patient-level identifiers, paired 2D and 3D models, controlled variation in culture conditions, and harmonized or cross-platform CRISPR measurements. Nonlinear models incorporating network or pathway structure, domain-adaptation strategies, and richer multi-omic representations are also reasonable candidates for evaluation. Their value, however, should be assessed without outcome-guided retuning and under the same prediction-lock and independent-validation discipline used here. The results document modest rank transfer within the selected gene panel. Whether the retrospective within-organoid ranking gains improve prospective experimental prioritization beyond tumor lineage remains to be established.

## 5. Conclusions

This study shows that transcriptome-based models learned in 2D cancer models retain a weak ability to rank patient-derived 3D organoids by dependency for genes in the preselected panel, without retraining and with substantial attenuation. More complex omic representations did not provide a reliable advantage over the expression-only model. Its macro Spearman correlation of 0.4094 in the locked 2D holdout provides internal confirmation conditional on historical target selection, not an unbiased generalization estimate. Performance decreased to 0.1518 in the Broad-94 exploratory cohort and to 0.1015 in the Sanger-148 blinded, independent external validation cohort. The Sanger association nevertheless exceeded the permutation-based null expectation. Eighteen of 20 target estimates were positive in Broad-94, 17 of 20 were positive in Sanger-148, and 15 of 20 were positive in both cohorts. The combined 3D macro correlation of 0.1231 therefore supports a modest, directionally reproducible rank-transfer signal rather than complete cross-system generalizability. The primary endpoint did not establish added predictive value beyond tumor lineage in Sanger. These results support transfer of predictive associations without establishing their causal basis. Broad uncertainty and retrospective pooled evidence remain conditional on incomplete donor ascertainment. The attenuation is an observed cross-domain effect; its biological and technical causes were not experimentally disentangled and remain uncertain.

*CCNE1*, *CDK6*, and *MDM4* received the strongest cross-cohort support and represent priorities for direct functional investigation rather than established therapeutic sensitivities. The Tiriac pancreatic projection showed that the frozen models could be applied to an independent transcriptomic cohort while retaining aspects of the target hierarchy. However, measured CRISPR outcomes were unavailable, and expression-domain shift was substantial. These limitations preclude interpreting that analysis as validation. The separate retrospective within-organoid analysis showed modest gains over lineage-only prediction for full 20-gene rankings in both cohorts, but a top-three overlap gain only in Sanger. These findings provide limited evidence for ranking genes within the selected panel. They do not establish dependency transferability for unselected genes or at genome scale. Prospective experimental prioritization beyond tumor lineage remains unestablished. The computational approach does not replace direct organoid CRISPR screening, pharmacological validation, or assessment of clinical utility. Prospective evaluation in larger, tumor-type-balanced, preregistered organoid cohorts will be required to establish the biological robustness and translational value of the prioritized targets.

## Data Availability

This study analyzed publicly available or openly released datasets. The 2D molecular profiles and CRISPR Gene Effect data were obtained from the DepMap Public 26Q1 release and the CCLE [3,4,13,14] (https://depmap.org/portal/data_page/?tab=allData) (accessed on 26 September 2026). The Broad NextGen organoid data were obtained from the *NextGen Model Manuscript 2026* resource and the associated Figshare dataset [38] (https://doi.org/10.6084/m9.figshare.29472362.v1). The Sanger tumor-derived organoid dependency data were obtained from the associated Figshare dataset [39] (https://doi.org/10.6084/m9.figshare.28339340). The Tiriac pancreatic organoid RNA-seq data were obtained from the GDC ORGANOID-PANCREATIC project [32] (https://portal.gdc.cancer.gov/projects/ORGANOID-PANCREATIC) (accessed on 26 September 2026). The analysis code, trained models, processed data, and supporting results are available in the study’s reproducibility repository on Zenodo [41]. Original source datasets remain available from their respective repositories under the applicable access and licensing conditions.

## Supplementary Materials

The following supporting information can be downloaded at: https://www.mdpi.com/article/10.3390/biology15191782/s1, Table S1: Development-cohort partition and fixed target panel; Table S2: Omics feature blocks and candidate input representations; Table S3: Frozen nested cross-validation, candidate-selection, and development-lock protocol; Table S4: Locked two-dimensional holdout evaluation and deployment-model finalization protocol; Table S5: Broad-94 organoid harmonization, prediction lock, and exploratory evaluation protocol; Table S6: Sanger-148 cohort construction, outcome-blinded prediction lock, and independent external-validation protocol; Table S7: Retrospective cross-cohort transportability synthesis protocol; Table S8: Tiriac pancreatic organoid cohort processing and secondary transcriptomic projection protocol; Table S9: Statistical-analysis hierarchy, inferential units, and multiplicity control; Table S10: Development-cohort candidate comparison and target-level out-of-fold performance; Table S11: Locked two-dimensional holdout performance and deployment-model finalization; Table S12: Exploratory transfer to Broad-94 organoids and target-level results; Table S13: Blinded independent external validation in Sanger-148 organoids; Table S14: Retrospective cross-cohort transportability synthesis across Broad-94 and Sanger-148 organoids; Table S15: Model projections and expression-domain diagnostics in Tiriac pancreatic organoids; Table S16: Retrospective assessment of external feature-coverage thresholds; Table S17: Retrospective evaluation of gene ranking within individual organoids; Table S18: Retrospective overlap of predicted and measured top-three gene sets; Table S19: Sanger sensitivity analysis using one averaged profile per donor; Table S20: Retrospective sensitivity of Tiriac projections and gene rankings to missing-feature imputation; Table S21: Target-specific missing features and projection shifts in Tiriac; Table S22: Broad cohort-size reconciliation and donor metadata coverage; Table S23: Retrospective Broad target-wise donor sensitivity analyses; Table S24: Retrospective Broad within-organoid ranking sensitivities.
