# Supplementary material for: Transferability of Transcriptome-Based Gene-Dependency Models from Two-Dimensional Cancer Models to Patient-Derived Organoids: Blinded External Validation
Source: Biology (Basel). 2026 Oct 8;15(19):1782. doi: 10.3390/biology15191782 (PMC13664187; doi:10.3390/biology15191782)
Supplement: Supplementary file 1 [file biology-15-01782-s001.zip › biology-4598534-supplementary.pdf]

Supplementary Materials

Transferability of Transcriptome-Based Gene-Dependency Models from Two-Dimensional Cancer Models to Patient-Derived Organoids: Blinded External Validation

Tables S1–S24

Supporting Materials and Methods Sections 2.2–2.10 and Results Sections 3.1–3.7, with additional retrospective analyses in Tables S17–S24

Table S1. Development-cohort partition and fixed target panel.

(a) Patient-grouped partition of the two-dimensional development resource

Characteristic	Value
Analysis-ready two-dimensional models	852
Development models	681
Locked two-dimensional test models	171
Development patient groups	658
Locked-test patient groups	165
PatientID overlap between partitions	0
Locked-test fraction	0.20
Random seed	20260826
Grouping variable	PatientID; ModelID fallback when PatientID was unavailable
Development validation design	5 outer × 5 inner patient-grouped, lineage-stratified nested cross-validation

Note. *Patient grouping used PatientID, with ModelID as the fallback only when PatientID was unavailable. No PatientID was shared between the development and locked-holdout partitions.*

(b) Fixed 20-gene target panel

Gene symbol	Entrez Gene identifier	Gene symbol	Entrez Gene identifier
BRAF	673	*IGF1R*	3480
CCND3	896	*KEAP1*	9817
CCNE1	898	*MAPK1*	5594
CDK6	1021	*MDM4*	4194
CTNNB1	1499	*NFE2L2*	4780
E2F3	1871	*RAF1*	5894
EGFR	1956	*STK11*	6794
EP300	2033	*TSC1*	7248
ERBB3	2065	*TSC2*	7249
FGFR1	2260	*YAP1*	10413

Note. *The target panel was fixed before the present omics-candidate comparison and was not altered after the 171-model locked holdout was opened. Because the historical target-selection workflow used all 852 2D models, the locked holdout was independent of the current candidate-representation, feature-selection, hyperparameter, and model-fitting decisions but not fully independent of historical target-panel selection. Genes are listed alphabetically; table order does not indicate a performance ranking.*

Table S2. Omics feature blocks and candidate input representations.

(a) Feature blocks used to construct the candidate representations

Feature block	Processed source	Encoding or scale	Initial features	Training-partition eligibility
Gene expression	DepMap Public 26Q1 protein-coding expression; X2D_expression.npy	log2(TPM+1); no second log transformation	19,215	Missing fraction ≤0.20 and variance >1 × 10^{-8}
Copy number	DepMap Public 26Q1 WGS gene-level copy number; X2D_copy_number.npy	Linear, ploidy-relative values	19,955	Missing fraction ≤0.20 and variance >1 × 10^{-8}
Damaging mutation	Somatic variants; X2D_mutation_damaging.npy	Binary gene-level carrier indicator	19,626	Variance >1 × 10^{-8}
Hotspot mutation	Somatic variants; X2D_mutation_hotspot.npy	Binary gene-level carrier indicator	19,626	Variance >1 × 10^{-8}
Lineage	OncotreeLineage	29 binary one-hot indicators	29	Variance >1 × 10^{-8} when screened within all-omics; all 29 indicators retained in the lineage-only reference

Note. *TPM, transcripts per million; WGS, whole-genome sequencing.*

(b) Candidate input representations compared in the development cohort

Candidate representation	Included feature blocks	Initial dimension	Analytical role
Lineage-only	Lineage	29	Non-molecular reference; excluded from the molecular one-standard-error selection rule
Expression-only	Gene expression	19,215	Molecular candidate; complexity rank 1
Expression + copy number	Gene expression + copy number	39,170	Molecular candidate; complexity rank 2
Expression + mutation	Gene expression + damaging mutation + hotspot mutation	58,467	Molecular candidate; complexity rank 3
All omics + lineage	All four molecular blocks + lineage	78,451	Molecular candidate; complexity rank 4

Note. *Initial dimensions preceded training-partition eligibility filtering. For each molecular candidate and target, the 300 eligible features with the highest absolute Pearson correlation were retained within the relevant training partition. Nonfinite values were temporarily replaced with training-partition means for screening; model fitting used training-median imputation followed by training-fitted standardization. These operations were repeated within the nested cross-validation structure. The locked 171-model holdout and all 3D data were excluded from preprocessing and feature selection.*

Table S3. Frozen nested cross-validation, candidate-selection, and development-lock protocol.

(a) Nested validation and target-specific model fitting

Component	Frozen specification	Leakage-control role
Development population	681 models representing 658 patient groups	The only population used for candidate comparison, feature learning and hyperparameter tuning
Grouping key	PatientID; ModelID fallback when PatientID was unavailable	No patient group was allowed to cross a training/validation boundary
Stratification variable	OncotreeLineage; categories with fewer than five models in the current partition were collapsed to Other_Rare for splitting only	Maintained broad lineage balance without changing model inputs or reported lineage labels
Randomization	Global seed 20260826; fold- and target-specific seeds were deterministically derived	Made the split registry and model fits reproducible
Outer validation	Five patient-grouped, lineage-stratified folds	Each development model contributed to an outer-test prediction exactly once
Inner tuning	Five patient-grouped, lineage-stratified folds within each outer-training partition	Separated hyperparameter selection from outer performance estimation
Fold-local feature learning	Eligibility filtering, absolute-Pearson top-300 screening, median imputation and standardization refitted in each training partition	Prevented validation or test observations from defining features or transformations
Molecular estimator	Target-specific Elastic Net regression for each of 20 targets	Allowed sparse and grouped effects in a high-dimensional setting
Elastic Net grid	l1_ratio={0.10, 0.50, 0.90}; alpha={0.001, 0.01, 0.1}	Prespecified search space; no outcome-dependent grid expansion
Fit controls	max_iter=20,000; tolerance=0.0001; cyclic coordinate updates	Convergence was audited for every selected outer model
Inner objective	Mean target-specific Spearman correlation across the five inner-validation folds	Matched the prespecified rank-based prediction objective
Hyperparameter rule	Fully converged settings first; then highest mean inner Spearman, larger alpha and lower l1_ratio. If none fully converged: most converged folds, highest mean inner Spearman, then larger alpha	Applied a deterministic target-specific choice rule
Outer refit	Selected setting refitted on the complete outer-training partition and applied once to the outer-test partition	Generated leakage-controlled out-of-fold predictions
Lineage-only reference	Target-specific Ridge regression; alpha={0.1, 1, 10, 100}	Non-molecular comparator; not eligible to win the molecular candidate-selection rule

Note. *All split, preprocessing, feature-selection, and tuning operations were learned only from the relevant training partition. Rare-lineage collapsing was used for fold stratification only and did not alter the original lineage labels used in analysis or reporting.*

Table S3. Cont.

(b) Candidate selection and development lock

Component	Frozen specification	Role in the analysis
Primary candidate score	Mean of the 20 target-specific OOF Spearman correlations (macro Spearman)	Sole primary score for candidate ranking
Secondary diagnostics	Target-wise MAE, RMSE, R^2, calibration intercept/slope and outer-fold feature-set Jaccard stability	Reported for diagnosis; not used to reopen the selection rule
Selection uncertainty	2,000 PatientID-cluster bootstrap replicates of the development OOF predictions	Estimated the bootstrap standard error and 95% interval while preserving patient clustering
One-standard-error rule	Best molecular OOF macro Spearman minus the bootstrap standard error of that best candidate	Defined the performance-equivalent candidate set
Complexity order	Expression-only < expression + copy number < expression + mutation < all omics + lineage	Selected the least complex molecular candidate meeting the one-standard-error threshold
Final target settings	Most frequently selected outer-fold l1_ratio and alpha pair; ties resolved by mean inner Spearman and then larger alpha	Converted fold-specific tuning into one frozen setting per target
Final development feature sets	Top 300 target-specific features relearned using all 681 development models after candidate selection	Prepared the selected model for one-time locked-test prediction without using locked-test outcomes
Development lock	Candidate identity, split registry, decision table and file digests recorded before locked-test outcome evaluation	Prevented candidate, feature, hyperparameter or metric changes after the holdout was opened
Information excluded during development	Locked 171-model outcomes and all three-dimensional data during preprocessing, hyperparameter tuning and candidate selection	Locked two-dimensional outcomes were opened only after prediction lock; three-dimensional data remained unused throughout development

Note. *OOF, out-of-fold; MAE, mean absolute error; RMSE, root mean squared error; R^2, coefficient of determination; SHA-256, 256-bit Secure Hash Algorithm. Initial target-specific feature selection and all preprocessing steps were repeated within the applicable training partition. In the current omics-candidate workflow, locked-holdout predictions were written and cryptographically sealed before the observed holdout outcome matrix was indexed for evaluation; no 3D file was read during development. The historical target-panel independence limitation is described in Table S1.*

Table S4. Locked two-dimensional holdout evaluation and deployment-model finalization protocol.

(a) One-time locked-holdout evaluation

Component	Frozen or implemented specification	Purpose
Analytical role	One-time locked two-dimensional internal holdout evaluation; not an independent external validation	Estimated performance of the frozen current model while retaining the historical target-panel limitation described in Table S1
Training and evaluation populations	681 development models (658 patient groups) and 171 locked-test models (165 patient groups); zero PatientID overlap	Kept all patient groups on one side of the split
Frozen model specification	Expression-only winner; 20 target-specific Elastic Net models; 300 target-specific expression features and final l1_ratio and alpha pair per target	Prevented post-holdout candidate, target, feature or hyperparameter changes
Development fit and holdout prediction	Median imputation, standardization and coefficients fitted on the 681-model development set and applied unchanged to the 171-model test set	Excluded locked-test observations from all fitted transformations
Prediction lock and outcome access	Model/feature manifests and locked_holdout_predictions.npy/.csv were SHA-256 sealed before the observed locked-test Gene Effect rows were indexed for evaluation	Established a verifiable prediction-before-outcome sequence
Primary and secondary measures	Primary: mean of 20 target-wise Spearman correlations. Secondary: target-wise Spearman, MAE, RMSE, R^2 and calibration intercept/slope	Kept candidate evaluation rank-based while retaining error and calibration diagnostics
Cluster-bootstrap uncertainty	2,000 bootstrap resamples of PatientID clusters; all rows from a sampled patient group retained; percentile 95% intervals	Preserved within-patient dependence
Development-to-holdout difference	Iteration-wise locked-holdout macro Spearman minus the corresponding development OOF bootstrap value	Quantified change from development OOF performance without reopening selection
Lineage-only comparison	Target-specific Ridge reference refitted on 681 models and applied to the same 171 models; model difference calculated with common holdout cluster resamples	Estimated incremental rank information beyond lineage alone
Within-lineage permutation sensitivity	Models aggregated to PatientID means; 20-target observed outcome vectors permuted among patient groups within each OncotreeLineage stratum for 2,000 iterations; one-sided empirical $p = (1 + \text{null} \geq \text{observed})/2{,}001$	Tested whether association exceeded that expected from lineage composition while predictions remained fixed

Note. *The holdout was evaluated once after the prediction lock. OOF, out-of-fold; MAE, mean absolute error; RMSE, root mean squared error; R^2, coefficient of determination; SHA-256, 256-bit Secure Hash Algorithm. The patient-aggregated macro Spearman correlation was retained as an additional sensitivity estimate.*

Table S4. Cont.

(b) Final all-852 deployment model and lock

Component	Frozen or implemented specification	Purpose
Training population	All 852 analysis-ready two-dimensional models	Used all available two-dimensional information only after the 681→171 evaluation was complete
Components held fixed	Expression-only candidate, 20-target panel, 300 target-specific feature identities, target-specific l1_ratio and alpha settings and primary metric	Prevented the locked-test result from changing model specification
Components re-estimated	Training medians, standardization means/standard deviations and Elastic Net coefficients	Allowed final coefficient estimation on all 852 models without feature or tuning reselection
Target-specific model bundles	20 serialized models containing feature indices/names/types, imputer, scaler, estimator, training count, model role and protocol hash	Made every downstream prediction traceable to a complete preprocessing-model bundle
Convergence requirement	All 20 deployment fits required convergence under max_iter=20,000 and tolerance=0.0001	Prevented non-converged models from entering external application
Separation from reported evaluation	The all-852 refit was not used to revise the development OOF or locked-test performance estimates	Preserved the reported 681→171 evaluation as the internal performance estimate
Deployment lock records	DEPLOYMENT_MODEL_LOCK.json and FINAL_2D_MODEL_LOCK.json linked the model manifest, selected-feature record, protocol and preceding lock files by SHA-256 digests	Created an auditable chain from development selection to final deployment
Three-dimensional application rule	No retraining, target/feature reselection, hyperparameter tuning or metric switching after access to new three-dimensional outcomes; blind predictions written and hash-locked before outcome merging	Maintained prediction-before-outcome separation in subsequent Broad and Sanger applications
Integrity verification	Model counts, split counts, patient overlap, target count, finite predictions, convergence and absence of three-dimensional input during development were checked before package sealing	Required all predefined verification checks to pass before release

Note. *The deployment refit used the locked-holdout outcomes only for final coefficient estimation after the internal evaluation had been completed; the outcomes were not used for candidate, target, feature, hyperparameter, or metric selection. The deployment model was the only selected molecular model authorized for subsequent 3D prediction and could not revise the reported 2D internal performance estimate.*

Table S5. Broad-94 organoid harmonization, prediction lock, and exploratory evaluation protocol.

(a) Cohort harmonization and prediction-before-outcome lock

Component	Frozen or implemented specification	Purpose
Analytical role	Retrospective exploratory three-dimensional transfer evaluation; not an independent external validation	The cohort had been accessible in earlier methodological work, so the current prediction-before-outcome sequence did not make it a pristine blind validation cohort.
Cohort resource	94 Broad NextGen organoid models with gene-expression data and CRISPR Gene Effect outcomes for all 20 locked targets	Provided a common expression-outcome evaluation set without changing the fixed target panel.
Analysis and alignment key	ModelID; PatientID was unavailable in the Broad evaluation records	Enabled deterministic expression-prediction-outcome alignment, while limiting inference to the model level. Original Broad inference assumed model independence; partial donor information and conditional sensitivities are reported in Tables S22–S24.
Frozen deployment model	Expression-only model refitted on all 852 two-dimensional models; 20 target-specific Elastic Net bundles	No Broad observation contributed to training, feature selection, hyperparameter tuning or coefficient estimation.
Target-specific feature sets	300 locked Entrez-identified expression features per target; target identities and l1_ratio and alpha settings unchanged	Preserved the exact feature and model specification authorized by the deployment lock.
Feature-coverage gate	Prespecified minimum coverage 80% (at least 240/300 frozen predictors); observed coverage 300/300 for every target (100%).	Pragmatic input-eligibility rule limiting unavailable predictors to 20%; not optimized against external performance. For the retrospective threshold check, see Table S16.
Three-dimensional preprocessing	Deployment-bundle medians, standardization parameters and Elastic Net coefficients applied unchanged in locked feature order	No Broad-fitted imputation, scaling, feature relearning or coefficient adaptation was permitted.
Outcome-separated prediction output	A finite 94-organoid × 20-target prediction matrix was written before outcome merging	Separated prediction generation from evaluation and retained every locked target.
Prediction-before-outcome seal	Prediction matrix, feature/model manifests and lock record were SHA-256 sealed before the current-run three-dimensional outcome matrix was extracted	Created an auditable temporal sequence despite the cohort's exploratory historical status.
Outcome access and integrity checks	After sealing, the 20 observed Gene Effect columns were aligned by ModelID; 21/21 core and 62/62 independent recomputation checks passed	Verified target order, finite values, lock integrity and reproducibility without reopening the model.

Note. *Broad-94 was retained as an exploratory 3D evaluation because it had been accessible during earlier methodological work. The audited current run nevertheless preserved a prediction-before-outcome sequence. Feature coverage was complete for all 20 target-specific 300-gene sets; no outcome-guided substitution or refitting was performed.*

Table S5. Cont.

(b) Exploratory performance estimation and sensitivity analyses

Component	Frozen or implemented specification	Purpose
Primary estimand	Arithmetic mean of the 20 target-specific Spearman correlations (macro target-wise Spearman)	Measured panel-wide rank transfer from the two-dimensional model to Broad organoids.
Target-level estimates	Spearman correlation for every locked target; no target removed according to effect direction or statistical result	Prevented selective reporting of favorable targets.
Secondary diagnostics	Target-wise MAE, RMSE, R^2, calibration intercept and calibration slope	Assessed absolute-scale transfer but did not alter the rank-based primary analysis.
Lineage-stratified bootstrap	5,000 model-level resamples with replacement within OncotreeLineage; percentile 95% intervals	Preserved observed lineage counts while quantifying uncertainty.
Patient-clustering limitation	PatientID was unavailable; resampling and primary inference therefore used organoid models rather than patient clusters	Makes possible same-donor dependence an explicit limitation. Original Broad inference assumed model independence; partial donor information and conditional sensitivities are reported in Tables S22–S24.
Lineage-only reference	Target-specific Ridge reference applied to the same 94 models; molecular-minus-lineage difference evaluated with paired bootstrap samples	Estimated incremental rank information beyond tumor-lineage composition.
Lineage-adjusted association	Target-wise partial Spearman correlations after adjustment for lineage indicators	Assessed whether pooled associations persisted after accounting for lineage composition.
Panel-wide permutation test	5,000 within-lineage permutations of complete 20-target outcome rows with predictions fixed; one-sided empirical $p=(1 + \text{null} \geq \text{observed})/5{,}001$	Preserved cross-target outcome structure and lineage composition under the null.
Multiple-testing control	Benjamini-Hochberg false discovery rate adjustment across 20 target-level permutation p values	Controlled multiplicity for target-specific exploratory inference.
Lineage subgroup analysis	Macro Spearman reported for lineage groups containing at least seven organoid models	Avoided numerical subgroup claims for extremely small lineages.
Leave-one-lineage-out sensitivity	Macro Spearman recomputed after excluding each lineage in turn	Evaluated whether the pooled signal was driven by a single lineage.
Resampling randomization	Global Broad-analysis seed 20260829; deterministic bootstrap and permutation sequences	Made the exploratory resampling workflow reproducible.
Post-outcome restrictions	No retraining, recalibration, feature/target reselection, hyperparameter tuning or metric switching	Separated Broad evaluation from model development and later Sanger validation.

Note. Broad-94 inference was model-level because PatientID was unavailable in the original evaluation records; later partial-donor sensitivities appear in Tables S22–S24. MAE, mean absolute error; RMSE, root mean squared error; R^2, coefficient of determination; FDR, false discovery rate; SHA-256, 256-bit Secure Hash Algorithm. All Broad outcomes were used only after prediction locking and were not used to retrain, recalibrate, or otherwise modify the frozen model.

Table S6. Sanger-148 cohort construction, outcome-blinded prediction lock, and independent external-validation protocol.

(a) Cohort construction and outcome-blinded prediction preparation

Component	Frozen or implemented specification	Independence or analytical role
Analytical role	One-time, outcome-blinded independent external validation of the frozen all-852 expression-only deployment model.	Kept the Sanger cohort outside model development and outside the Broad-94 exploratory analysis.
Screened resource	162 organoids representing 157 unique donors with RNA-seq data and quality-controlled CRISPR screening records.	Defined eligibility without using the locked 20-target Sanger outcomes for model selection or tuning.
Cross-resource identity audit	Sanger sample and donor identifiers were cross-referenced against Broad NextGen mappings and the previously used Broad-94 registry.	Prevented exact-model reuse and identifiable donor linkage across the exploratory and independent cohorts.
Conservative exclusions	13 Broad-linked organoids plus one additional organoid from the same donor as a Broad-linked model were excluded.	Removed all Broad-linked models and the additional same-donor record before prediction.
Final validation cohort	148 organoids representing 144 donors; no Broad-link flag, same-donor Broad link or exact Broad-94 identity overlap remained.	Established the cohort used for the single external-validation evaluation.
Tumor-type composition	Colorectal 81 models/79 donors; esophageal 49/49; ovarian 11/9; pancreatic 4/4; gastric 3/3.	Supported prespecified secondary analyses in the two largest groups and cautious interpretation of smaller groups.
Assay eligibility	RNA-seq input and CRISPR screening coverage were present for all included organoids; all 20 locked targets were experimentally covered.	Produced a complete fixed-panel evaluation without outcome-dependent target exclusion.
RNA-seq harmonization	Sanger TPM values were transformed as log2(TPM+1) and mapped to locked Entrez Gene identifiers.	Aligned the external transcriptome with the two-dimensional deployment feature space without refitting the model.
Frozen feature mapping	The fixed 300 expression features for each target were ordered exactly as stored in the corresponding deployment-model bundle.	Prevented Sanger data from changing feature identity, order or target-specific model specification.
Feature coverage and missingness	3,790 of 3,845 unique locked genes were measured; 55 were absent. Target-wise coverage was 291-300/300 (97.0%-100%), above the frozen 80% threshold. Absent inputs were handled only by the two-dimensional fitted imputer.	Allowed application without Sanger-fitted imputation, scaling, feature selection or coefficient adaptation. The rationale is given in Table S5; the retrospective threshold check is in Table S16.
Pre-outcome coverage correction	Before outcome access, the coverage key array was restricted to the 3,790 measured genes. Original and corrected blind predictions were bitwise identical; only coverage reporting changed.	Documented a metadata correction without altering predictions or scientific results.
Blind prediction and cryptographic lock	A 148 × 20 prediction matrix, cohort registry, protocol, model manifest and feature manifest were written and SHA-256 sealed; all 11 prespecified seal checks passed before Sanger outcome access.	Created a verifiable prediction-before-outcome sequence.

Note. *The feature-coverage correction was completed before Sanger CRISPR outcomes were accessed. The original and corrected prediction arrays were bitwise identical; only the coverage metadata were corrected. TPM, transcripts per million; SHA-256, 256-bit Secure Hash Algorithm.*

Table S6. Cont.

(b) External outcome, primary estimand, and robustness analyses

Component	Frozen or implemented specification	Independence or analytical role
External outcome	Corrected and scaled gene-level Sanger CRISPR log fold-change (LFC); more negative values indicate stronger dependency.	Defined the measured external endpoint independently of the prediction model.
CRISPR library selection	For 13 dual-library organoids, the official Sanger covariate selected minLib when code=1 and v1_1 when code=0; the available quality-controlled screen was used for single-library organoids.	Applied a pre-outcome, externally defined rule rather than choosing the result that best matched predictions.
Complete evaluation grid	148 organoids × 20 locked targets = 2,960 finite prediction-outcome pairs in the frozen sample and target order.	Prevented missing-result or direction-based target filtering.
Primary estimand	Target-wise Spearman correlation between blind predictions and Sanger LFC, followed by the arithmetic mean across the 20 targets (macro target-wise Spearman).	Gave every locked target equal weight in the primary external-validation statistic.
Scale rationale	Rank correlation was primary because Sanger corrected LFC and Broad/DepMap Chronos Gene Effect are not on an identical absolute scale; absolute-scale error metrics were not used for the primary external-validation decision.	Avoided treating assay-specific numerical scales as directly interchangeable.
Donor-cluster bootstrap	5,000 donor resamples with replacement (seed 20260829); all organoids from a sampled donor were retained together; percentile 95% confidence intervals.	Preserved within-donor dependence in macro and target-wise uncertainty estimates.
Within-lineage permutation	Predictions were fixed; repeated organoids were first averaged by donor, then each donor-level 20-target outcome vector was permuted within tumor-type/lineage strata for 5,000 iterations (seed 20260830). The two-sided empirical p value was (1 + the number of absolute null statistics at least as large as the observed absolute statistic)/5,001.	Tested the panel-wide association while preserving cross-target structure and broad tumor-type composition.
Multiple testing	Benjamini-Hochberg false discovery rate correction across the 20 target-level permutation p values.	Controlled target-wise multiplicity without changing the fixed panel.
Lineage-only comparator	A pre-outcome frozen comparator based on patient-weighted two-dimensional lineage means was applied to the same 148 organoids.	Estimated rank information attributable to tumor origin alone.
Incremental and adjusted analyses	Expression-minus-lineage macro difference used paired donor-cluster bootstrap samples; partial Spearman adjusted for primary tumor-type indicators.	Separated molecular information from lineage-associated structure without retuning the expression model.
Repeated-donor sensitivities	One model per donor retained the lexicographically first sample identifier (144 models); a separate donor-mean analysis averaged predictions and outcomes within donor.	Assessed whether the four repeated-donor records materially affected the primary result.
Subgroup hierarchy	Colorectal and esophageal analyses were prespecified secondary; ovarian was exploratory; pancreatic and gastric were descriptive only.	Prevented small subgroups from being interpreted as confirmatory evidence.
Independent replay and no-retuning rule	Cohort size, donor count, overlap exclusion, 2,960-pair completeness, feature coverage, target/prediction order, Spearman statistics, bootstrap, permutation, lineage adjustment and file hashes were independently recomputed; 22/22 checks passed. No model, target, feature, hyperparameter or metric was changed after outcome unlock.	Confirmed computational integrity and preserved the one-way external-validation design.

***Note.** LFC, log fold-change; FDR, false discovery rate. The validation cohort was evaluated once after the prediction lock. The rank-based primary estimand was retained because assay-specific dependency scales were not treated as numerically interchangeable. The independent replay reproduced all prespecified integrity and statistical checks without changing predictions or analysis rules.*

Table S7. Retrospective cross-cohort transportability synthesis protocol.
(a) Directional concordance and target-level meta-analytic synthesis

Component	Frozen or implemented specification	Analytical role or interpretation
Analytical role	Retrospective synthesis of already completed Broad-94 exploratory and Sanger-148 blinded independent external-validation target results.	Summarized cross-cohort transportability; it was not treated as a third external validation or confirmatory replication.
Input records	Frozen 20-target Broad-94 and Sanger-148 performance records plus the locked two-dimensional holdout target metrics; no model refitting or new feature learning.	Kept the synthesis downstream of all cohort-specific prediction and evaluation steps.
Included endpoints	Broad: target-wise Spearman correlation against Chronos Gene Effect in 94 ModelID units. Sanger: target-wise Spearman correlation against corrected CRISPR LFC in 148 organoids, with donor-aware uncertainty.	Used rank-based outcomes while retaining the distinct assay and clustering structures of the two cohorts. Original Broad inference assumed model independence; partial donor information and conditional sensitivities are reported in Tables S22–S24.
Evidence excluded from the CRISPR synthesis	The Tiriac pancreatic projection lacked measured genome-scale CRISPR outcomes and was not pooled with the Broad and Sanger CRISPR results.	Prevented a projection-only endpoint from being treated as measured dependency evidence.
Target harmonization	The same 20 locked targets were aligned by Entrez Gene identifier and frozen target order; no target was removed according to direction, effect size or significance.	Preserved the prespecified panel and prevented selective target inclusion.
Directional concordance	A target was directionally concordant when both the Broad-94 and Sanger-148 Spearman correlations were greater than zero.	Defined the cross-cohort sign-replication count without using an effect-size threshold.
Exact-binomial benchmark	One-sided exact binomial comparison of the common-positive count with n=20 and p0=0.50.	Used only as a descriptive sign benchmark. Correlated targets violate the independence assumption; the synthesis was retrospective.
Cross-cohort target-rank concordance	Two-sided Spearman correlation between the vectors of 20 Broad and 20 Sanger target-specific correlations.	Assessed whether relative target performance ordering was retained across cohorts.
Cohort-specific uncertainty	Broad uncertainty from 5,000 lineage-stratified model-level bootstrap replicates; Sanger uncertainty from 5,000 donor-cluster bootstrap replicates.	Retained the analysis unit available in each cohort when deriving target-level uncertainty. Original Broad inference assumed model independence; partial donor information and conditional sensitivities are reported in Tables S22–S24.
Target-wise fixed-effect pooling	Each cohort correlation was Fisher-z transformed; inverse-variance weights were derived from cohort-specific bootstrap intervals (lineage-stratified model-level resampling for Broad; donor-cluster resampling for Sanger); the pooled z estimate was back-transformed to Spearman rho.	Produced one combined rank-transfer estimate for every locked target.
Target-level inference and multiplicity	Two-sided meta-analytic p values followed by Benjamini-Hochberg false discovery rate correction across the 20 targets.	Controlled multiplicity without changing the fixed panel.
Heterogeneity sensitivity	Random-effects estimates and I^2 were retained as descriptive outputs only because two cohorts provide unstable heterogeneity estimation.	Prevented overinterpretation of between-cohort heterogeneity.
Macro synthesis and target-panel bootstrap	Macro 3D performance was the arithmetic mean of the 20 pooled target correlations. The 20 targets were resampled with replacement for 20,000 iterations; percentile 95% intervals were calculated.	Quantified sensitivity to the composition of the locked target panel; intervals were descriptive rather than new-cohort sampling intervals.
Two-dimensional-to-three-dimensional attenuation	For each target, Broad or Sanger rho minus the corresponding locked two-dimensional holdout rho; macro differences and 95% intervals used the same 20,000 target resamples.	Quantified domain-related performance attenuation without reopening model selection.

Note. *The synthesis used previously completed Broad-94 and Sanger-148 target results and did not constitute a third external validation. LFC, log fold-change; FDR, false discovery rate; I^2, heterogeneity statistic. Target-panel bootstrap intervals describe sensitivity to the composition of the locked 20-target panel and should not be interpreted as new-cohort sampling intervals.*

Table S7. Cont.

(b) Correlation-aware sensitivity analysis and evidence tiers

Component	Frozen or implemented specification	Analytical role or interpretation
Permutation analysis units	Broad used ModelID-level rows because PatientID was unavailable. Sanger repeated organoids were averaged within donor before permutation.	Matched the strongest available independence unit in each cohort. Original Broad inference assumed model independence; partial donor information and conditional sensitivities are reported in Tables S22–S24.
Blockwise simultaneous permutation	Predictions remained fixed; each unit's complete 20-target observed outcome vector was moved as one block among units within each cohort.	Preserved cross-target covariance and the cohort-specific analysis-unit structure.
Correlation-aware repetitions	50,000 simultaneous two-cohort permutations; the number of targets remaining positive in both cohorts was recomputed in every iteration.	Evaluated the directional result under a null distribution that retained target correlation.
Empirical permutation p value	One-sided p=(1 + number of null common-positive counts ≥ observed count)/(50,001).	Applied a finite-sample correction to the post-hoc sensitivity test.
Interpretation of joint permutation	Post-hoc correlation-aware sensitivity analysis only; not a new cohort, independent validation or confirmatory replication.	Kept the evidence hierarchy explicit.
Expression-versus-lineage comparison	Target-wise expression-only rho minus lineage-only rho, evaluated separately in Broad and Sanger by one-sided Wilcoxon signed-rank tests.	Assessed whether molecular predictions added rank information beyond tumor origin at the target-panel level.
Tier A: strongest cross-cohort support	Positive rho in both cohorts, meta-analytic BH q<0.05, and BH q<0.05 in at least one individual cohort.	Required directional agreement, combined multiplicity control and individual-cohort support.
Tier B: directional support	Positive rho in both cohorts but failure to meet all Tier A criteria.	Separated replicated direction from stronger statistical support.
Tier C: inconsistent or uncertain	At least one cohort had rho ≤ 0.	Flagged sign inconsistency without deleting the target.
Use of evidence tiers	Retrospective reporting classification only; tiers did not change models, targets, features, hyperparameters, metrics or the synthesis inputs.	Prevented evidence grading from becoming outcome-guided model revision.
No-retuning and no-cherry-picking rule	No retraining, feature/target reselection, hyperparameter tuning, metric switching or outcome-direction target removal was allowed during the retrospective synthesis.	Preserved all previously locked prediction and evaluation decisions.

Note. *The exact-binomial sign benchmark and the 50,000-iteration joint permutation were descriptive or post hoc because the 20 targets were correlated and the synthesis was planned after the cohort-specific results were known. Evidence tiers were reporting labels only. No target, model, feature, hyperparameter, or metric was changed on the basis of synthesis results.*

Table S8. Tiriac pancreatic organoid cohort processing and secondary transcriptomic projection protocol.
(a) Cohort construction, expression processing, and frozen-model application

Component	Frozen or implemented specification	Analytical role or interpretation
Analytical role	Secondary biological/transcriptomic projection after completion of two-dimensional development, Broad-94 exploration, Sanger-148 validation and retrospective synthesis.	The Tiriac cohort was not classified as external validation because genome-scale measured CRISPR dependency outcomes were unavailable.
Source project	GDC ORGANOID-PANCREATIC / Tiriac pancreatic organoid cohort; open STAR-count files obtained from the official public S3 mirror.	Used an independent pancreatic organoid transcriptomic resource without adding it to model development.
File inventory	55 open RNA-seq STAR-count files; source file identifiers, sizes, source-provided MD5 values, S3 locations and local SHA-256 digests retained in the source manifest.	Preserved file-level provenance and integrity.
Cohort composition	44 tumor samples from 38 independent tumor cases and 11 normal samples from 11 independent normal cases.	Separated sample count from the case-level inferential population.
Group definition	Tumor versus normal status taken from the source TissueType field; all records were annotated as Next Generation Cancer Model samples.	Used source metadata without outcome-driven relabeling.
Expression quantity	tpm_unstranded from the GDC STAR output.	Used the available abundance field as the model input quantity.
Duplicate-gene handling	Rows mapping to the same gene symbol were summed at the TPM level.	Produced one abundance value per consolidated gene symbol before transformation.
Expression transformation	log2(TPM+1).	Matched the scale expected by the locked expression-only deployment model.
Frozen model	Twenty target-specific expression-only Elastic Net deployment bundles finalized in all 852 two-dimensional models.	No Tiriac sample contributed to fitting, feature selection or tuning.
Components held fixed	Twenty-target panel; target-specific feature identities; stored imputation and standardization objects; l1_ratio-alpha settings; fitted coefficients and projection metric definitions.	Prevented model revision in the application cohort.
Unique locked-feature coverage	3,806 of 3,845 unique selected features observed in the Tiriac expression data (98.99%).	Documented overall compatibility of the application matrix with the frozen model space.
Target-specific coverage gate	292-300 of 300 selected features present per target (97.33%-100%); prespecified pass threshold ≥80%; all 20 targets passed.	Prevented application of a target model with inadequate input coverage. The rationale is given in Table S5; the retrospective threshold check is in Table S16.
Missing locked features	Absent features were initialized as NaN and filled with the medians stored in each frozen all-852 deployment model before the stored scaler and coefficients were applied.	Preserved the training preprocessing rule without fitting replacement values in Tiriac. All 1,100 original predictions were reproduced exactly. Retrospective alternatives are evaluated in Tables S20 and S21.
Projection output	A 55-sample × 20-target matrix of predicted Gene Effect values.	Generated model outputs only; no observed CRISPR outcome matrix was available for accuracy estimation.
No-retuning rule	No retraining, recalibration, target/feature reselection, hyperparameter tuning or metric switching in response to Tiriac data or summaries.	Kept the pancreatic analysis downstream of the frozen model pipeline.

Note. *Tiriac dependency scores are model projections, not experimentally measured dependencies. No measured genome-scale CRISPR dependency outcomes were available. GDC, Genomic Data Commons; STAR, Spliced Transcripts Alignment to a Reference; S3, Amazon Simple Storage Service; TPM, transcripts per million; MD5, MD5 message-digest algorithm; SHA-256, 256-bit Secure Hash Algorithm.*

Table S8. Cont.

(b) Case-level comparisons, cross-cohort concordance, and expression-domain diagnostics

Component	Frozen or implemented specification	Analytical role or interpretation
Inferential unit	Repeated tumor organoid samples from the same GDC case were averaged; analyses used 38 tumor and 11 normal case-level records.	Reduced within-case replication and defined the patient/case as the unit of inference.
Target-wise group summaries	Median case-level projection in tumor and normal groups for each of the 20 locked targets.	Summarized the central projected dependency distribution within each biological group.
Tumor-normal estimand	Tumor median minus normal median for each target.	Negative values indicated a more negative (stronger) projected dependency in tumors.
Bootstrap uncertainty	5,000 patient/case-level bootstrap replicates; percentile 95% interval from the 2.5th and 97.5th percentiles.	Quantified uncertainty in the projected tumor-normal median difference.
Distributional test	Two-sided Mann-Whitney U test comparing tumor and normal case-level projections for each target.	Assessed group separation without assuming normality.
Multiple testing	Benjamini-Hochberg false discovery rate correction across the 20 target-wise tumor-normal tests.	Controlled multiplicity within the fixed panel.
Effect-size orientation	Cliff delta; positive values were defined as a more negative and therefore stronger predicted dependency distribution in tumors.	Kept the effect direction consistent with the negative Gene Effect convention.
Interpretation of group tests	Tests compared frozen model outputs, not measured CRISPR dependencies or model errors.	Prevented tumor-normal projection differences from being presented as validation.
Cross-cohort target-rank concordance	Two-sided Spearman correlation of the 20-target Tiriac tumor median projection vector with the corresponding Broad-94 and Sanger-148 median prediction vectors.	Assessed whether the target hierarchy was retained across organoid transcriptomic cohorts.
Concordance boundary	All compared values were predictions from the same locked model family; no Tiriac CRISPR outcome was available.	Treated concordance as secondary biological consistency, not independent predictive accuracy.
Common domain-shift space	3,790 locked expression features jointly available in Tiriac, Broad-94 and Sanger-148.	Restricted domain diagnostics to a common feature space.
Multivariable shift diagnostic	Principal component analysis of the common locked expression space.	Visualized cohort separation without changing model inputs or predictions.
Feature-wise shift diagnostic	Standardized mean difference for Tiriac versus Broad-94 and Tiriac versus Sanger-148; summaries included median absolute SMD and percentages with $\|SMD\|>1$ and $\|SMD\|>2$.	Quantified the breadth and magnitude of expression-domain shift.
Use of shift diagnostics	Diagnostic reporting only; no recalibration, feature deletion, target removal or model revision was allowed.	Preserved the frozen application analysis despite observed domain differences.
Claim boundary	Allowed terminology: projected, applied and prioritized. Disallowed for the Tiriac layer: validated dependency, confirmed dependency or therapeutic sensitivity.	Aligned the reported claim with the absence of measured genome-scale CRISPR outcomes.
Reproducibility setting	Analysis seed 20260830; 5,000 bootstrap replicates; source and output manifests archived with cryptographic digests.	Made the secondary analysis traceable and repeatable.

Note. *Tumor-normal statistics compared frozen model outputs rather than observed dependencies. Cross-cohort concordance compared target-median predictions from the same locked model family and was not an independent accuracy test. PCA, principal component analysis; SMD, standardized mean difference; FDR, false discovery rate. Domain-shift diagnostics were descriptive and did not alter any model or target decision.*

Table S9. Statistical-analysis hierarchy, inferential units, and multiplicity control.

(a) Analysis layers, inferential units, and uncertainty estimation

Analysis layer	Primary estimand and inferential unit	Uncertainty or resampling specification
Development candidate comparison	681 models representing 658 PatientID groups; target-wise OOF Spearman correlations and their 20-target arithmetic mean (macro Spearman).	2,000 PatientID-cluster bootstrap replicates. Bootstrap standard error defined the one-standard-error candidate rule; percentile intervals were retained for uncertainty reporting.
Locked two-dimensional holdout	171 models representing 165 PatientID groups; target-wise Spearman and macro Spearman. Patient-aggregated estimates were retained as sensitivity analyses.	2,000 PatientID-cluster bootstrap replicates. Development-to-holdout and expression-versus-lineage comparisons used iteration-matched or common cluster resamples.
Broad-94 exploratory three-dimensional evaluation	94 organoid ModelIDs; PatientID was unavailable. Target-wise Spearman and macro Spearman were the rank-based estimands.	5,000 lineage-stratified model-level bootstrap replicates. Expression-versus-lineage differences used paired resamples; model-level inference was retained as a stated limitation. Original Broad inference assumed model independence; partial donor information and conditional sensitivities are reported in Tables S22–S24.
Sanger-148 blinded external validation	148 organoids representing 144 donors; target-wise Spearman and macro Spearman, with donor-level sensitivity estimates.	5,000 donor-cluster bootstrap replicates. All organoids from a sampled donor were retained together; paired model comparisons used the same donor resamples.
Retrospective Broad-Sanger synthesis	The fixed 20-target panel was the synthesis unit. Target correlations were combined after Fisher-z transformation; the macro summary was the mean of the 20 combined correlations.	Inverse-variance weights were derived from cohort-specific bootstrap uncertainty. Target-panel sensitivity used 20,000 target bootstrap replicates; random-effects estimates and I^2 were descriptive.
Tiriac pancreatic projection	Repeated tumor organoids were averaged within GDC case; 38 tumor and 11 normal cases were the inferential units. The target estimand was the tumor-minus-normal median projection difference.	5,000 case-level bootstrap replicates within tumor and normal groups. Target-rank concordance, PCA and SMD analyses were secondary descriptive diagnostics, not performance validation.

Note. *Percentile 95% intervals were defined by the 2.5th and 97.5th bootstrap percentiles unless a subsection explicitly stated another purpose. OOF, out-of-fold; GDC, Genomic Data Commons; PCA, principal component analysis; SMD, standardized mean difference;* I^2*, heterogeneity statistic. Resampling followed the strongest biological grouping available in each cohort.*

Table S9. Cont.

(b) Hypothesis tests, multiplicity, implementation, and interpretation rules

Analysis or test	Direction and multiplicity rule	Analytical interpretation
Locked two-dimensional within-lineage permutation	2,000 permutations of patient-aggregated 20-target outcome vectors within OncotreeLineage; one-sided empirical p with plus-one correction.	Panel-level sensitivity to lineage composition; predictions remained fixed and the result did not reopen model selection.
Broad-94 permutation and target tests	5,000 one-sided permutations of complete 20-target outcome vectors among ModelIDs within lineage. Target-level permutation p values were adjusted across 20 targets by Benjamini-Hochberg FDR.	Exploratory panel-wide chance assessment and target diagnostics; PatientID was unavailable. Original Broad inference assumed model independence; partial donor information and conditional sensitivities are reported in Tables S22–S24.
Sanger-148 permutation and target tests	5,000 two-sided permutations after donor averaging; complete outcome vectors were moved among donors within tumor-type/lineage strata. Target-level p values were adjusted across 20 targets by Benjamini-Hochberg FDR.	Primary blind external-validation chance assessment while preserving donor dependence and broad tumor-type composition.
Cross-cohort directional sign benchmark	One-sided exact binomial comparison of the number of targets with positive correlations in both cohorts; n=20 and null success probability 0.50.	Descriptive benchmark only. Target dependence limits the binomial interpretation; the synthesis was retrospective.
Cross-target joint permutation	50,000 one-sided simultaneous permutations; Broad ModelID and Sanger donor-mean 20-target outcome vectors were preserved as blocks. Empirical p used plus-one correction.	Post-hoc correlation-aware sensitivity analysis; did not constitute a new external validation. Original Broad inference assumed model independence; partial donor information and conditional sensitivities are reported in Tables S22–S24.
Target-level meta-analytic tests	Two-sided fixed-effect Fisher-z tests; 20 p values adjusted by Benjamini-Hochberg FDR. Random-effects estimates and I^2 were descriptive.	Summarized cross-cohort target support without treating Broad and Sanger dependency scales as numerically interchangeable.
Expression versus lineage-only comparison	Target-wise expression-minus-lineage Spearman differences evaluated separately in Broad and Sanger using one-sided Wilcoxon signed-rank tests.	Secondary assessment of molecular information beyond lineage; no pooled confirmatory claim across cohorts.
Tiriac tumor-normal projection tests	Two-sided Mann-Whitney U tests for each target; 20 p values adjusted by Benjamini-Hochberg FDR. Cliff delta reported effect direction.	Compared frozen predicted outputs, not observed CRISPR dependencies or therapeutic sensitivity.
Tiriac target-rank concordance and domain diagnostics	Two-sided Spearman tests across 20 target-median predictions for Broad and Sanger comparisons. PCA and SMD summaries had no confirmatory p-value.	Secondary concordance and distribution-shift diagnostics; not an independent accuracy analysis.
Software implementation	Python 3.12.13; NumPy 2.3.5; pandas 2.2.3; SciPy 1.17.0; scikit-learn 1.8.0; joblib 1.5.3. Random seeds and environment files were archived.	Preserved a versioned computational environment for independent verification.
General reporting rule	Tests were two-sided unless explicitly prespecified as one-sided. FDR $q<0.05$ defined multiplicity-adjusted target-level significance within the stated 20-target family.	Exact p values, effect sizes and intervals were reported together; exploratory and post-hoc analyses remained descriptive even when thresholds were crossed.

Note. *Empirical permutation p values used the standard plus-one numerator and denominator correction. FDR, false discovery rate; LFC, log fold-change. No analysis result was used to change the locked target panel, feature identities, hyperparameters, primary metric, or previously generated predictions.*

Table S10. Development-cohort candidate comparison and target-level out-of-fold performance.

(a) Candidate-level omics ablation in the 681-model development cohort

Candidate representation	OOF macro Spearman ρ	95% bootstrap interval	Mean feature-set Jaccard	Decision
Lineage only	0.2598	0.2388–0.2806	Not calculated	Reference
Expression only	0.4071	0.3893–0.4232	0.565	Selected
Expression + copy number	0.4081	0.3894–0.4243	0.554	Not selected
Expression + mutation	0.4073	0.3908–0.4235	0.554	Not selected
All omics + lineage	0.4031	0.3864–0.4184	0.550	Not selected

(b) Paired PatientID-cluster bootstrap differences relative to expression-only

Augmented representation	Observed Δρ	95% paired bootstrap interval	Interpretation
Expression + copy number	+0.0010	-0.0047 to +0.0066	Interval includes zero
Expression + mutation	+0.0002	-0.0075 to +0.0079	Interval includes zero
All omics + lineage	-0.0039	-0.0123 to +0.0043	Interval includes zero

Note. Candidate-level intervals and paired differences were obtained from 2,000 PatientID-cluster bootstrap replicates of the development OOF predictions. The lineage-only model was a non-molecular reference and was excluded from the molecular one-standard-error selection rule. Jaccard values summarize mean pairwise overlap of target-specific feature sets across the five outer folds; a Jaccard value was not calculated for the fixed lineage-only input set. OOF, out-of-fold.

Table S10. Cont.

(c) Target-level OOF Spearman correlations for the selected expression-only model

Gene symbol	OOF Spearman ρ	Gene symbol	OOF Spearman ρ
CDK6	0.693	*IGF1R*	0.407
STK11	0.516	*FGFR1*	0.392
CCND3	0.461	*CCNE1*	0.369
YAP1	0.415	*E2F3*	0.442
MDM4	0.480	*ERBB3*	0.317
EP300	0.457	*NFE2L2*	0.305
CTNNB1	0.352	*BRAF*	0.280
EGFR	0.449	*KEAP1*	0.402
TSC2	0.432	*MAPK1*	0.283
TSC1	0.432	*RAF1*	0.257

Note. Targets are shown in the fixed panel order and not ranked by performance. All 20 target-specific development OOF correlations were positive; their arithmetic mean was 0.4071 after reporting precision. Entrez Gene identifiers are provided in Table S1. The locked 171-model holdout and all three-dimensional data were excluded from this candidate comparison.

Table S11. Locked two-dimensional holdout performance and deployment-model finalization.

(a) Panel-wide holdout performance and lineage comparison

Component	Result	Interpretation
Analytical role	One-time locked two-dimensional internal holdout; not an independent external validation	Retained the historical target-panel limitation described in Table S1
Evaluation cohort	171 models representing 165 PatientID groups; zero PatientID overlap with development	All patient-related rows remained on one side of the split
Development OOF macro Spearman ρ	0.4071 (95% CI, 0.3893-0.4232)	Development estimate from the selected expression-only candidate
Locked-holdout macro Spearman ρ	0.4094 (95% CI, 0.3730-0.4383)	Primary one-time locked-holdout estimate
Holdout minus development OOF	+0.0023 (95% CI, -0.0372 to 0.0361)	The interval included zero; no detectable difference was observed within the preselected panel.
Positive locked-holdout targets	20/20	No target was removed according to direction or performance
Patient-aggregated holdout macro ρ	0.4133	Sensitivity estimate after averaging repeated models within PatientID
Lineage-only holdout macro ρ	0.2969 (95% CI, 0.2529-0.3368)	Non-molecular tumor-origin reference
Expression-only minus lineage-only	+0.1125 (95% CI, 0.0765-0.1472)	Paired PatientID-cluster bootstrap interval excluded zero
Within-lineage permutation	One-sided p=0.00050; 2,000 permutations; 165 PatientID groups	Observed 20-target outcome vectors permuted within lineage while predictions remained fixed

Note. Confidence intervals were percentile intervals from 2,000 PatientID-cluster bootstrap replicates. The development and holdout values were not used to reopen the model-selection rule. OOF, out-of-fold; CI, confidence interval.

Table S11. Cont.

(b) Target-level development and locked-holdout performance

Gene symbol	Entrez ID	Development OOF ρ	Locked-holdout ρ	Holdout MAE	Holdout R^2
CDK6	1021	0.693	0.532	0.350	0.195
STK11	6794	0.516	0.482	0.236	0.233
CCND3	896	0.461	0.471	0.187	0.411
YAP1	10413	0.415	0.509	0.214	0.202
MDM4	4194	0.480	0.460	0.166	0.300
EP300	2033	0.457	0.550	0.336	0.235
CTNNB1	1499	0.352	0.449	0.203	0.524
EGFR	1956	0.449	0.373	0.219	0.168
TSC2	7249	0.432	0.525	0.354	0.185
TSC1	7248	0.432	0.461	0.320	0.158
IGF1R	3480	0.407	0.352	0.202	0.086
FGFR1	2260	0.392	0.365	0.211	0.186
CCNE1	898	0.369	0.390	0.146	0.331
E2F3	1871	0.442	0.375	0.198	0.173
ERBB3	2065	0.317	0.352	0.140	0.179
NFE2L2	4780	0.305	0.456	0.183	0.346
BRAF	673	0.280	0.341	0.182	0.280
KEAP1	9817	0.402	0.377	0.365	0.121
MAPK1	5594	0.283	0.197	0.203	0.306
RAF1	5894	0.257	0.170	0.210	-0.021

Note. Targets are shown in the fixed panel order and not ranked by performance. All 20 locked-holdout correlations were positive. MAE, mean absolute error; R^2, coefficient of determination; OOF, out-of-fold.

Table S11. Cont.

(c) Target-level lineage comparison and secondary diagnostics

Gene symbol	Lineage-only holdout ρ	Expression-only - lineage-only Δρ	Holdout RMSE	Calibration intercept	Calibration slope
CDK6	0.478	+0.054	0.487	-0.172	0.695
STK11	0.314	+0.168	0.301	-0.043	0.793
CCND3	0.370	+0.101	0.269	-0.069	0.909
YAP1	0.357	+0.152	0.285	0.152	1.466
MDM4	0.247	+0.213	0.238	0.075	1.348
EP300	0.375	+0.175	0.459	0.125	1.474
CTNNB1	0.301	+0.148	0.310	-0.007	1.205
EGFR	0.272	+0.100	0.351	0.029	1.387
TSC2	0.409	+0.115	0.476	0.114	1.464
TSC1	0.351	+0.110	0.412	0.026	1.751
IGF1R	0.231	+0.121	0.267	-0.124	0.651
FGFR1	0.139	+0.225	0.358	-0.014	0.916
CCNE1	0.214	+0.176	0.221	0.259	2.073
E2F3	0.268	+0.107	0.256	-0.060	0.792
ERBB3	0.369	-0.017	0.300	0.082	1.474
NFE2L2	0.306	+0.150	0.280	0.077	1.535
BRAF	0.335	+0.006	0.282	-0.041	0.676
KEAP1	0.253	+0.124	0.481	0.057	1.654
MAPK1	0.246	-0.049	0.290	0.075	1.288
RAF1	0.102	+0.067	0.288	-0.059	0.447

Note. Target-level differences are descriptive and were not subjected to a target-selection rule. The prespecified panel-level expression-only minus lineage-only contrast used paired PatientID-cluster bootstrap replicates. RMSE, root mean squared error.

Table S11. Cont.

(d) Final all-852 deployment model and integrity verification

Component	Frozen or verified result	Analytical role
Deployment training population	All 852 analysis-ready two-dimensional models	Refit only after the 681-to-171 evaluation was complete
Components retained unchanged	Expression-only representation; 20 targets; 300 target-specific feature identities; target-specific l1_ratio-alpha settings; primary metric	Prevented holdout results from revising model specification
Components re-estimated	Training medians, standardization parameters and Elastic Net coefficients	Used all available two-dimensional information for final coefficient estimation
Target-feature inventory	6,000 target-feature selections representing 3,845 unique expression features	Preserved the frozen 300-feature set for each of 20 targets
Convergence	20/20 target-specific deployment models converged	No non-converged fit entered external application
Final deployment lock	18/18 lock-verification checks passed	Linked model, feature, protocol and preceding lock records by SHA-256
Strict audit	41/41 checks passed	Verified model counts, split counts, patient overlap, finite predictions, convergence and absence of 3D input
Independent replay	50/50 checks passed; maximum prediction-reproduction difference=0.0	Reproduced the locked-holdout predictions and macro Spearman exactly
Three-dimensional application rule	No retraining, target/feature reselection, hyperparameter tuning or metric switching; predictions written and hash-locked before outcome merging	Preserved the one-way prediction-before-outcome design

Note. The all-852 refit could not revise the reported development or locked-holdout performance estimates. No three-dimensional data contributed to training or finalization. SHA-256, 256-bit Secure Hash Algorithm.

Table S12. Exploratory transfer to Broad-94 organoids and target-level results.

(a) Panel-wide performance, lineage comparison, and analysis integrity

Measure	Result	Interpretation
Analytical role	Retrospective exploratory 3D transfer; not an independent external validation	Broad-94 had been accessible during earlier methodological work.
Cohort and target panel	94 organoid models; 20 locked targets	No target was removed according to direction or statistical result.
Frozen model	Expression-only Elastic Net deployment model refitted on all 852 2D models	No Broad observation contributed to training, feature selection, tuning or coefficient estimation.
Feature coverage	300/300 features for every target (100%)	No outcome-guided substitution or Broad-specific feature relearning was required.
Prediction-before-outcome sequence	Finite 94 × 20 prediction matrix written and SHA-256 locked before current-run outcome extraction	Preserved audited one-way information flow despite exploratory historical status.
Development OOF macro Spearman	0.4071	Internal development reference.
Locked 2D holdout macro Spearman	0.4094	Frozen two-dimensional holdout reference.
Broad-94 macro Spearman	0.1518 (95% CI, 0.1019-0.2006)	Primary exploratory panel-wide rank-transfer estimate.
Broad minus locked-2D difference	-0.2576	Shows attenuation after transfer from 2D to Broad organoids.
Median target-wise Spearman	0.1628	Median across the fixed 20-target panel.
Direction consistency	18/20 positive; 2/20 negative	Panel-level direction was predominantly positive.
Lineage-adjusted macro Spearman	0.1080	Partial Spearman after lineage-indicator adjustment.
Lineage-only macro Spearman	0.0951	Non-molecular reference in the same 94 organoids.
Expression-only minus lineage-only	0.0567 (95% CI, 0.0040-0.1106)	Paired lineage-stratified bootstrap contrast.
Within-lineage permutation	One-sided p=0.00080; 5,000 permutations	Complete 20-target outcome rows permuted within lineage while predictions remained fixed.
Target-level multiplicity	0/20 targets with BH FDR q<0.05; lowest q=0.0640 for CCNE1	No individual target was treated as confirmatorily replicated in Broad-94.
Integrity verification	21/21 core checks; 62/62 independent re-audit checks passed	Verified alignment, finite values, lock integrity, feature coverage and metric recomputation.

Note. CI, confidence interval; FDR, false discovery rate; OOF, out-of-fold; SHA-256, 256-bit Secure Hash Algorithm. PatientID was unavailable in the original Broad evaluation records; the original bootstrap and primary inference used 94 model units. Partial donor recovery and retrospective sensitivities are reported in Tables S22–S24; original intervals and permutation results remain conditional on model-level independence. The prediction-before-outcome sequence refers to the audited current analysis and does not convert Broad-94 into a pristine independent validation cohort.

Table S12. Cont.

(b) Target-level rank-transfer estimates

Gene	Spearman ρ	95% bootstrap CI	Lineage-adjusted ρ	Lineage-only ρ	BH q
ERBB3	0.3489	0.1585 to 0.5183	0.1763	0.2576	0.2831
CCNE1	0.3456	0.1746 to 0.5064	0.3103	-0.0435	0.0640
CTNNB1	0.3009	0.1180 to 0.4629	0.1809	0.3188	0.1460
E2F3	0.2679	0.0654 to 0.4542	0.2574	0.1280	0.1460
YAP1	0.2614	0.0648 to 0.4411	0.2138	0.1842	0.1460
CDK6	0.2447	0.0373 to 0.4349	0.2281	0.0434	0.1460
RAF1	0.2436	0.0397 to 0.4365	0.2019	0.2074	0.1460
MDM4	0.2000	-0.0217 to 0.3999	0.2143	0.2096	0.3584
NFE2L2	0.1824	-0.0003 to 0.3541	0.2107	0.0473	0.1460
TSC2	0.1651	-0.0365 to 0.3538	0.1632	0.1125	0.1460
FGFR1	0.1605	-0.0210 to 0.3384	0.0596	-0.1119	0.5468
BRAF	0.1121	-0.0950 to 0.3113	0.1135	0.1340	0.1835
TSC1	0.0940	-0.1042 to 0.2799	0.0637	-0.0123	0.3072
CCND3	0.0725	-0.1232 to 0.2692	0.0080	0.2621	0.6055
EP300	0.0619	-0.1305 to 0.2466	0.0698	0.0886	0.3584
KEAP1	0.0518	-0.1395 to 0.2470	-0.1426	-0.0620	0.8214
EGFR	0.0350	-0.1474 to 0.2125	-0.0145	0.0237	0.5714
IGF1R	0.0197	-0.1755 to 0.2106	0.0790	-0.0964	0.4339
MAPK1	-0.0307	-0.2211 to 0.1614	-0.1266	0.2068	0.8382
STK11	-0.1010	-0.3064 to 0.1096	-0.1075	0.0038	0.8382

Note. Targets are ordered by the Broad-94 Spearman point estimate for presentation only; all 20 frozen targets were analyzed. Confidence intervals were obtained from 5,000 lineage-stratified model-level bootstrap replicates. Benjamini-Hochberg q values adjust the 20 target-level within-lineage permutation p values. No target met q<0.05.

Table S12. Cont.

(c) Target-level permutation and absolute-scale diagnostics

Gene	Permutation p	MAE	RMSE	R²	Calibration intercept	Calibration slope
ERBB3	0.1416	0.2038	0.2916	0.1193	-0.0104	0.9922
CCNE1	0.0032	0.2651	0.3874	0.0023	0.1695	2.5975
CTNNB1	0.0584	0.5479	0.6827	-0.6248	-0.3139	0.3450
E2F3	0.0438	0.2056	0.2728	-0.1021	-0.2057	0.4397
YAP1	0.0534	0.3906	0.5923	-0.2291	0.9040	3.9062
CDK6	0.0238	0.4746	0.5627	-0.8824	0.0732	0.4936
RAF1	0.0466	0.2178	0.2955	0.0036	0.0059	0.6584
MDM4	0.2292	0.2676	0.3822	0.0856	0.2420	2.3345
NFE2L2	0.0370	0.2296	0.2852	-0.1859	0.0384	0.6525
TSC2	0.0538	0.1818	0.2336	-0.0352	-0.1240	0.4125
FGFR1	0.4101	0.1250	0.1627	-0.1695	-0.0599	0.3649
BRAF	0.0826	0.2710	0.3555	-0.4277	-0.0290	0.1540
TSC1	0.1690	0.2443	0.3149	-0.0844	-0.1393	0.2805
CCND3	0.5147	0.1643	0.1988	-0.1369	-0.1296	0.3684
EP300	0.2330	0.2709	0.3599	-0.0226	-0.1991	0.2890
KEAP1	0.7393	0.2404	0.3172	-0.0378	-0.3161	-0.0995
EGFR	0.4571	0.4249	0.5663	-0.0602	-0.2714	0.6332
IGF1R	0.3037	0.2703	0.3483	-0.2243	-0.1240	0.3073
MAPK1	0.8382	0.2538	0.3082	-0.4649	-0.0680	-0.2783
STK11	0.8116	0.2496	0.3294	-0.0245	-0.1171	0.2415

Note. Permutation p values are target-specific, one-sided within-lineage empirical values before multiplicity adjustment; the corresponding Benjamini-Hochberg q values are shown in Panel B. MAE, mean absolute error; RMSE, root mean squared error; R^2, coefficient of determination. Absolute-scale and calibration measures were secondary diagnostics and did not alter the rank-based primary analysis or the exploratory status of the Broad-94 cohort.

Table S12. Cont.

(d) Lineage subgroup and leave-one-lineage-out sensitivity analyses

Lineage	Models	Macro ρ	Median target ρ	Positive targets
Esophagus/Stomach	24	0.0531	0.0713	13/20
Pancreas	23	0.1247	0.1364	14/20
Bowel	18	-0.0099	-0.0650	9/20
Breast	14	0.1123	0.1538	15/20
Prostate	7	0.2786	0.3750	15/20

Leave-one-lineage-out analysis

Excluded lineage	Excluded n	Remaining n	Macro ρ	Change from all 94
Esophagus/Stomach	24	70	0.1832	+0.0314
Pancreas	23	71	0.1504	-0.0015
Bowel	18	76	0.1769	+0.0250
Breast	14	80	0.1306	-0.0212
Prostate	7	87	0.1202	-0.0316
Ovary/Fallopian Tube	2	92	0.1574	+0.0056
Uterus	2	92	0.1469	-0.0049
Ampulla of Vater	2	92	0.1456	-0.0062
Biliary Tract	2	92	0.1524	+0.0006

Note. Subgroup estimates were displayed only for lineages with at least seven Broad organoid models. Leave-one-lineage-out estimates were computed after removing each lineage in turn, including lineages too small for standalone subgroup reporting. The pooled macro Spearman remained positive after every exclusion. These analyses are sensitivity diagnostics, not independent lineage-specific validations.

Table S13. Blinded independent external validation in Sanger-148 organoids.

(a) Cohort construction and panel-wide validation

Characteristic	Result	Interpretation or audit role
Screened resource	162 organoids / 157 donors	RNA-sequencing and quality-controlled CRISPR records available before outcome access
Conservative exclusions	14 organoids	13 Broad-linked organoids plus one additional organoid from the same donor as a Broad-linked model
Final external-validation cohort	148 organoids / 144 donors	No Broad-link flag, same-donor Broad link, or exact Broad-94 identity overlap remained
Blind prediction-outcome grid	2,960 pairs	148 organoids × 20 frozen targets
Measured locked genes	3,790 / 3,845	55 unique locked genes were absent from Sanger RNA-sequencing data
Target-wise feature coverage	291-300 / 300 (97.0%-100%)	All targets exceeded the frozen 80% coverage threshold
Prediction-before-outcome seal	11 / 11 checks passed	Prediction matrix and model/feature manifests were SHA-256 sealed before outcome access
Primary expression-only macro Spearman	0.1015 (95% CI, 0.0637-0.1357)	5,000 donor-cluster bootstrap replicates
Panel-wide permutation	Two-sided $p = 0.00040$	5,000 donor-level permutations within tumor-type/lineage strata
Positive target-wise estimates	17 / 20	All 20 frozen targets retained
Targets with BH $q < 0.05$	3 / 20	CDK6, MDM4, and CCNE1
Tumor-type-adjusted macro Spearman	0.0882	Partial Spearman after primary tumor-type indicators
Lineage-only macro Spearman	0.0553 (95% CI, 0.0168-0.0917)	Frozen non-molecular reference
Expression minus lineage-only	+0.0462 (95% CI, -0.0065 to 0.0986)	Paired donor-cluster bootstrap interval crossed zero
One model per donor	0.1014	Lexicographically first sample retained for each of 144 donors
Donor-mean estimand	0.0990	Predictions and outcomes averaged within donor
Independent recomputation	22 / 22 checks passed	Cohort, targets, statistics, lock records, and hashes independently verified

Note. *CI, confidence interval; BH, Benjamini-Hochberg; SHA-256, 256-bit Secure Hash Algorithm. The Sanger cohort was not used for model development, feature selection, hyperparameter tuning, or the Broad-94 exploratory analysis.*

Table S13. Cont.

(b) Target-level rank-transfer estimates and multiple-testing results

Gene	Spearman ρ	95% CI lower	95% CI upper	Permutation p	BH q	Result
CDK6	0.3221	0.1666	0.4702	0.00020	0.0040	FDR q < 0.05
STK11	0.0662	-0.0988	0.2321	0.53009	0.7573	Positive; q >= 0.05
CCND3	-0.1404	-0.2993	0.0222	0.09218	0.3073	Negative; q >= 0.05
YAP1	0.1162	-0.0600	0.2834	0.36233	0.6588	Positive; q >= 0.05
MDM4	0.2992	0.1483	0.4410	0.00060	0.0040	FDR q < 0.05
EP300	0.1682	0.0017	0.3313	0.06679	0.2671	Positive; q >= 0.05
CTNNB1	0.2831	0.1031	0.4563	0.14617	0.4176	Positive; q >= 0.05
EGFR	-0.0752	-0.2279	0.0838	0.40252	0.6709	Negative; q >= 0.05
TSC2	0.0605	-0.0926	0.2198	0.50210	0.7573	Positive; q >= 0.05
TSC1	0.0077	-0.1604	0.1707	0.97401	0.9798	Positive; q >= 0.05
IGF1R	0.1015	-0.0664	0.2583	0.22935	0.5097	Positive; q >= 0.05
FGFR1	0.0022	-0.1671	0.1653	0.89222	0.9798	Positive; q >= 0.05
CCNE1	0.2792	0.1014	0.4493	0.00040	0.0040	FDR q < 0.05
E2F3	0.0001	-0.1726	0.1701	0.97980	0.9798	Positive; q >= 0.05
ERBB3	0.0187	-0.1526	0.1852	0.88342	0.9798	Positive; q >= 0.05
NFE2L2	-0.0076	-0.1644	0.1448	0.92841	0.9798	Negative; q >= 0.05
BRAF	0.1120	-0.0452	0.2700	0.22535	0.5097	Positive; q >= 0.05
KEAP1	0.2967	0.1256	0.4464	0.01120	0.0560	Positive; q >= 0.05
MAPK1	0.0048	-0.1623	0.1674	0.92601	0.9798	Positive; q >= 0.05
RAF1	0.1147	-0.0476	0.2734	0.26235	0.5247	Positive; q >= 0.05

Note. *Targets are shown in the frozen lock order. Spearman correlations compare blinded expression-only predictions with Sanger gene-level log fold-change values across 148 organoids. Confidence intervals were obtained from 5,000 donor-cluster bootstrap replicates. Permutation p values are two-sided donor-level values calculated within tumor-type/lineage strata; BH q values control the false discovery rate across 20 targets. More negative Sanger log fold-change indicates stronger measured dependency.*

Table S13. Cont.

(c) Lineage-adjusted, lineage-only, and donor-sensitivity estimates

Gene	Tumor-type-adjusted ρ	Lineage-only ρ	Expression - lineage Δρ	Donor-mean ρ	One model/donor ρ
CDK6	0.2917	0.2173	0.1048	0.3397	0.3554
STK11	0.0064	-0.0743	0.1405	0.0680	0.0527
CCND3	-0.1346	-0.0447	-0.0957	-0.1379	-0.1227
YAP1	0.1005	0.2428	-0.1266	0.0774	0.0772
MDM4	0.2867	0.0735	0.2257	0.3096	0.3005
EP300	0.1359	-0.0136	0.1819	0.1651	0.1617
CTNNB1	0.0776	0.3603	-0.0773	0.2664	0.2561
EGFR	-0.0297	0.1342	-0.2094	-0.0755	-0.0664
TSC2	0.0494	0.0760	-0.0156	0.0521	0.0668
TSC1	0.0609	-0.0445	0.0522	0.0029	0.0176
IGF1R	0.1485	0.0353	0.0661	0.0931	0.1156
FGFR1	0.0414	-0.0468	0.0490	0.0121	0.0006
CCNE1	0.2862	-0.0738	0.3530	0.2885	0.3028
E2F3	0.0575	-0.1539	0.1540	-0.0022	-0.0170
ERBB3	0.0486	-0.0719	0.0907	0.0120	0.0355
NFE2L2	0.0458	0.1437	-0.1513	-0.0091	-0.0112
BRAF	0.0599	0.0485	0.0634	0.1207	0.1243
KEAP1	0.1967	0.3493	-0.0526	0.2770	0.2693
MAPK1	-0.0137	-0.1743	0.1791	0.0069	0.0078
RAF1	0.0480	0.1225	-0.0078	0.1142	0.1023

Note. *Tumor-type-adjusted values are partial Spearman correlations after primary tumor-type indicators. The lineage-only reference was frozen before outcome access. Donor-mean estimates average repeated organoids within donor; the one-model-per-donor sensitivity retains the lexicographically first sample for each donor.*

Table S13. Cont.

(d) Tumor-type subgroups, feature coverage, and validation integrity

Tumor type	Models	Donors	Macro ρ	Positive targets	Targets assessed	Interpretation
Colorectal	81	79	0.1030	15/20	20	Prespecified secondary
Esophageal	49	49	0.0654	13/20	20	Prespecified secondary
Ovarian	11	9	0.1755	15/20	20	Exploratory
Pancreatic	4	4	0.0300	9/20	20	Descriptive Only
Gastric	3	3	0.1500	12/20	20	Descriptive Only

Validation-integrity checks

Check	Observed result	Status
Complete outcome grid	2,960 / 2,960 finite values	PASS
Target order	Matched frozen 20-target lock order	PASS
Prediction order	Matched frozen Sanger cohort order	PASS
Feature coverage	All 20 targets above 80%; observed 97.0%-100%	PASS
Dual-library organoids	13; prespecified minLib/v1_1 rule applied	PASS
Pre-outcome coverage correction	Initial and corrected blind predictions bit-identical	PASS
Outcome direction	Mean depleted LFC -0.9133 vs non-depleted -0.1359	PASS
Independent recomputation	22 / 22 predefined checks	PASS

Note. *Colorectal and esophageal estimates were prespecified secondary analyses. Ovarian results were exploratory; pancreatic and gastric results were descriptive because of very small sample sizes. The pre-outcome coverage correction changed only the coverage report and not the blind prediction array. LFC, log fold-change.*

Table S14. Retrospective cross-cohort transportability synthesis across Broad-94 and Sanger-148 organoids.

(a) Panel-wide synthesis, sign replication, and evidence-tier summary

Measure	Result	Interpretation
Broad-94 cohort	94 organoid models	Exploratory 3D transfer cohort
Sanger-148 cohort	148 organoids / 144 donors	Blinded independent external-validation cohort
Common-positive targets	15 / 20	Targets with positive Spearman correlations in both cohorts
Exact-binomial sign benchmark	One-sided p = 0.0207	Descriptive because the synthesis was retrospective and targets were correlated
Correlation-aware simultaneous permutation	14 / 20 common-positive donor-aggregated target effects; one-sided empirical p = 1.0×10^{-4}	Post hoc sensitivity analysis preserving cross-target structure
Fixed-effect combined macro Spearman	0.1231	Mean of the 20 fixed-effect target-level meta-analytic correlations
Target-panel bootstrap 95% CI	0.0769 to 0.1704	20,000 target-bootstrap replicates
Locked 2D holdout macro Spearman	0.4094	Reference performance before 3D transfer
Combined 3D minus locked 2D	-0.2863	Attenuation relative to the locked 2D holdout
Meta-analytic BH q < 0.05	8 / 20 targets	Target-level fixed-effect meta-analysis
Evidence tiers	Tier A = 3; Tier B = 12; Tier C = 5	Tier A required positivity in both cohorts, meta-analytic q < 0.05, and target-level q < 0.05 in at least one cohort
Cross-cohort rank-order correlation	Spearman ρ = 0.2782; two-sided p = 0.2350	Agreement in relative target ordering across the two 3D cohorts

Note. BH, Benjamini-Hochberg; CI, confidence interval. The exact-binomial comparison was descriptive only; target dependence limits its binomial interpretation. The correlation-aware simultaneous permutation was performed post hoc and did not constitute a new external validation.

Table S14. Cont.

(b) Target-level fixed-effect synthesis results

Gene	Broad-94 ρ	Sanger-148 ρ	Fixed-effect meta ρ	95% CI	BH q	Tier
CDK6	0.2447	0.3221	0.2924	0.1660 to 0.4094	1.01×10^{-4}	Tier A
STK11	-0.1010	0.0662	0.0022	-0.1290 to 0.1333	0.9740	Tier C
CCND3	0.0725	-0.1404	-0.0543	-0.1799 to 0.0730	0.5039	Tier C
YAP1	0.2614	0.1162	0.1789	0.0476 to 0.3042	0.0260	Tier B
MDM4	0.2000	0.2992	0.2655	0.1400 to 0.3825	2.36×10^{-4}	Tier A
EP300	0.0619	0.1682	0.1213	-0.0058 to 0.2445	0.1363	Tier B
CTNNB1	0.3009	0.2831	0.2922	0.1631 to 0.4114	1.02×10^{-4}	Tier B
EGFR	0.0350	-0.0752	-0.0280	-0.1462 to 0.0911	0.7178	Tier C
TSC2	0.1651	0.0605	0.1003	-0.0242 to 0.2217	0.1902	Tier B
TSC1	0.0940	0.0077	0.0441	-0.0828 to 0.1696	0.5839	Tier B
IGF1R	0.0197	0.1015	0.0675	-0.0586 to 0.1915	0.3922	Tier B
FGFR1	0.1605	0.0022	0.0734	-0.0512 to 0.1957	0.3542	Tier B
CCNE1	0.3456	0.2792	0.3128	0.1865 to 0.4290	5.18×10^{-5}	Tier A
E2F3	0.2679	0.0001	0.1093	-0.0243 to 0.2392	0.1902	Tier B
ERBB3	0.3489	0.0187	0.1571	0.0267 to 0.2822	0.0460	Tier B
NFE2L2	0.1824	-0.0076	0.0720	-0.0471 to 0.1890	0.3542	Tier C
BRAF	0.1121	0.1120	0.1120	-0.0150 to 0.2355	0.1675	Tier B
KEAP1	0.0518	0.2967	0.1896	0.0604 to 0.3126	0.0168	Tier B
MAPK1	-0.0307	0.0048	-0.0103	-0.1356 to 0.1155	0.9195	Tier C
RAF1	0.2436	0.1147	0.1631	0.0343 to 0.2865	0.0379	Tier B

Note. *Targets are shown in the frozen lock order. Fixed-effect meta-analytic correlations were obtained on the Fisher-z scale using inverse-variance weights derived from cohort-specific bootstrap uncertainty intervals and were transformed back to the Spearman-correlation scale. Tier A targets were positive in both cohorts, had combined BH q < 0.05, and had target-level BH q < 0.05 in at least one cohort. Tier B targets were positive in both cohorts without meeting all Tier A criteria. Tier C targets had a non-positive result in at least one cohort.*

Table S14. Cont.

(c) Cohort-specific lineage comparison and additional synthesis diagnostics

Diagnostic or contrast	Result	Interpretation
Broad-94 expression-only vs lineage-only	One-sided Wilcoxon signed-rank $p = 0.0379$	Target-wise expression-only ρ minus lineage-only ρ
Sanger-148 expression-only vs lineage-only	One-sided Wilcoxon signed-rank $p = 0.1012$	Target-wise expression-only ρ minus lineage-only ρ
Broad-94 positive targets	18 / 20	Exploratory 3D transfer cohort
Sanger-148 positive targets	17 / 20	Blinded independent external-validation cohort
Strongest Tier A target	CCNE1: Broad ρ = 0.3456; Sanger ρ = 0.2792; meta ρ = 0.3128; $q = 5.18 \times 10^{-5}$	Highest combined target-level estimate
Additional Tier A targets	CDK6: meta ρ = 0.2924, $q = 1.01 \times 10^{-4}$; MDM4: meta ρ = 0.2655, $q = 2.36 \times 10^{-4}$	Cross-cohort support with target-level FDR evidence in at least one cohort
Highest Tier B meta-analytic targets	CTNNB1: meta ρ = 0.2922, $q = 1.02 \times 10^{-4}$; KEAP1: 0.1896, q = 0.0168; ERBB3: 0.1571, q = 0.0460	Positive in both cohorts without Tier A status
Cross-cohort attenuation	Broad minus locked 2D = -0.2576; Sanger minus locked 2D = -0.3079	Lower performance was observed across domains; biological and technical causes remain unresolved.

Note. *The lineage-only reference was frozen before outcome access in each cohort. The Wilcoxon analyses were secondary panel-level comparisons across the 20 locked targets and were not used for target selection. CTNNB1 met combined BH $q < 0.05$ but remained Tier B because neither cohort achieved target-level BH $q < 0.05$ individually. FDR, false discovery rate.*

Table S14. Cont.

(d) Target-level sign pattern and evidence-tier assignment

Gene	Broad/Sanger sign	Positive in both cohorts	Evidence tier
CDK6	+/+	Yes	Tier A
STK11	-/+	No	Tier C
CCND3	+/-	No	Tier C
YAP1	+/+	Yes	Tier B
MDM4	+/+	Yes	Tier A
EP300	+/+	Yes	Tier B
CTNNB1	+/+	Yes	Tier B
EGFR	+/-	No	Tier C
TSC2	+/+	Yes	Tier B
TSC1	+/+	Yes	Tier B
IGF1R	+/+	Yes	Tier B
FGFR1	+/+	Yes	Tier B
CCNE1	+/+	Yes	Tier A
E2F3	+/+	Yes	Tier B
ERBB3	+/+	Yes	Tier B
NFE2L2	+/-	No	Tier C
BRAF	+/+	Yes	Tier B
KEAP1	+/+	Yes	Tier B
MAPK1	-/+	No	Tier C
RAF1	+/+	Yes	Tier B

Note. *The sign pattern uses the Broad-94 target-wise Spearman-correlation sign followed by the Sanger-148 sign. This panel supports the reported counts of 15 common-positive targets, 3 Tier A targets, 12 Tier B targets, and 5 Tier C targets. The classification was used for reporting only and did not alter the prespecified target panel, feature sets, hyperparameters, or trained models.*

Table S15. Model projections and expression-domain diagnostics in Tiriac pancreatic organoids.

Dependency scores and tumor–normal contrasts are model projections, not experimentally measured dependencies. Cross-cohort concordance compares model predictions; expression-domain diagnostics describe differences in input distributions.

(a) Cohort composition, frozen-feature coverage, and analytical role

Component	Result	Interpretation
Analytical role	Secondary biological/transcriptomic projection; not external validation	Tiriac had no measured genome-scale CRISPR dependency outcome
RNA-sequencing samples	55	44 tumor samples and 11 normal samples
Case-level inferential population	38 independent tumor cases and 11 independent normal cases	Repeated tumor organoids were averaged within GDC case
Locked target models	20	The target panel and target-specific models were unchanged
Unique locked expression features	3,845	Union of the 20 target-specific 300-feature sets
Unique features observed in Tiriac	3,806 / 3,845 (98.99%)	Absent frozen features were represented according to the locked projection protocol
Target-wise feature coverage	292-300 / 300 (97.33%-100%)	All targets exceeded the prespecified 80% coverage gate
Model adaptation	None	No retraining, recalibration, target/feature reselection, or hyperparameter tuning
Analysis seed and bootstrap	Seed 20260830; 5,000 case-level bootstrap replicates	Reproducibility setting for tumor-normal projection contrasts

Target-specific frozen-feature coverage

Target	Selected features	Present features	Coverage	80% gate
CDK6	300	298	99.33%	PASS
STK11	300	297	99.00%	PASS
CCND3	300	297	99.00%	PASS
YAP1	300	297	99.00%	PASS
MDM4	300	297	99.00%	PASS
EP300	300	297	99.00%	PASS
CTNNB1	300	292	97.33%	PASS
EGFR	300	300	100.00%	PASS
TSC2	300	298	99.33%	PASS
TSC1	300	298	99.33%	PASS
IGF1R	300	298	99.33%	PASS
FGFR1	300	298	99.33%	PASS
CCNE1	300	296	98.67%	PASS
E2F3	300	298	99.33%	PASS
ERBB3	300	299	99.67%	PASS
NFE2L2	300	296	98.67%	PASS
BRAF	300	298	99.33%	PASS
KEAP1	300	299	99.67%	PASS
MAPK1	300	297	99.00%	PASS
RAF1	300	297	99.00%	PASS

Note. *Feature coverage was assessed before any tumor-normal comparison. All model bundles used the locked target-specific feature order and coefficients. The Tiriac cohort did not contribute to model fitting or selection. GDC, Genomic Data Commons.*

Table S15. Cont.

(b) Target-level case projections and tumor-normal contrasts

Gene	Tumor median	Normal median	Tumor - normal	95% bootstrap CI	Cliff delta	Two-sided p	BH q	Focal	Top-3 cases	External evidence context
CTNNB1	-1.2821	-0.8476	-0.4346	-0.5641 to -0.2979	0.5981	0.0029	0.0044	No	37	Two-cohort directional
CDK6	-0.7534	-0.5532	-0.2002	-0.2431 to -0.1588	0.9043	6.28×10^{-6}	2.09×10^{-5}	Yes	37	Sanger FDR + Broad positive
YAP1	-0.4810	-0.5834	0.1023	0.0726 to 0.1128	-0.9282	3.54×10^{-6}	1.77×10^{-5}	No	24	Two-cohort directional
BRAF	-0.4354	-0.4131	-0.0223	-0.0812 to 0.0670	0.0478	0.8199	0.8631	No	12	Two-cohort directional
EGFR	-0.4139	-0.4601	0.0462	0.0218 to 0.0659	-0.7273	2.83×10^{-4}	5.15×10^{-4}	No	2	Inconsistent/uncertain
E2F3	-0.3342	-0.4399	0.1057	0.0703 to 0.2157	-0.8086	5.40×10^{-5}	1.20×10^{-4}	No	1	Two-cohort directional
CCNE1	-0.2930	-0.2656	-0.0273	-0.0506 to -0.0091	0.4689	0.0195	0.0260	Yes	0	Sanger FDR + Broad positive
KEAP1	-0.2889	-0.2021	-0.0868	-0.1058 to -0.0684	0.9761	1.08×10^{-6}	1.08×10^{-5}	No	0	Two-cohort directional
NFE2L2	-0.2727	-0.3251	0.0524	0.0076 to 0.1027	-0.4737	0.0183	0.0260	No	1	Inconsistent/uncertain
IGF1R	-0.2573	-0.1160	-0.1413	-0.1782 to -0.0470	0.8086	5.40×10^{-5}	1.20×10^{-4}	No	0	Two-cohort directional
RAF1	-0.2511	-0.1373	-0.1138	-0.1537 to -0.0772	0.9904	7.49×10^{-7}	1.08×10^{-5}	No	0	Two-cohort directional
CCND3	-0.2462	-0.1555	-0.0907	-0.1350 to -0.0702	0.9187	4.46×10^{-6}	1.78×10^{-5}	No	0	Inconsistent/uncertain
EP300	-0.2182	-0.1838	-0.0345	-0.0564 to -0.0033	0.3780	0.0600	0.0750	No	0	Two-cohort directional
MAPK1	-0.2095	-0.2158	0.0063	-0.0145 to 0.0453	-0.1675	0.4084	0.4805	No	0	Inconsistent/uncertain
TSC2	-0.2079	-0.1577	-0.0502	-0.0721 to -0.0151	0.6077	0.0024	0.0041	No	0	Two-cohort directional
ERBB3	-0.1929	-0.1939	0.0010	-0.0161 to 0.0187	0.0144	0.9522	0.9522	No	0	Two-cohort directional
STK11	-0.1422	-0.1581	0.0159	-0.0165 to 0.0378	-0.1244	0.5412	0.6013	No	0	Inconsistent/uncertain
MDM4	-0.0756	-0.3202	0.2446	0.2167 to 0.2774	-0.9569	1.75×10^{-6}	1.17×10^{-5}	Yes	0	Sanger FDR + Broad positive
TSC1	-0.0459	0.0105	-0.0565	-0.0846 to -0.0288	0.7990	6.62×10^{-5}	1.32×10^{-4}	No	0	Two-cohort directional
FGFR1	0.0486	-0.0311	0.0797	0.0544 to 0.1036	-0.8804	1.10×10^{-5}	3.14×10^{-5}	No	0	Two-cohort directional

Note. *More negative projected Gene Effect values indicate stronger projected dependency within each target-specific model. Negative tumor-minus-normal values indicate a stronger projected dependency distribution in tumor cases. Positive Cliff delta was defined as a more negative tumor distribution. The tests compare frozen model outputs; they do not test experimentally measured dependencies, predictive accuracy or therapeutic sensitivity. Fifteen of 20 contrasts had BH q < 0.05.*

Table S15. Cont.

(c) Cross-cohort target-ranking concordance

Comparison	Targets	Spearman ρ	Two-sided p	Interpretation
Tiriac tumor vs Broad-94	20	0.7173	3.71×10^{-4}	Secondary concordance of median predictions from the same locked model family
Tiriac tumor vs Sanger-148	20	0.7805	4.91×10^{-5}	Secondary concordance of median predictions from the same locked model family
Broad-94 vs Sanger-148	20	0.9248	5.54×10^{-9}	Reference concordance between the two prior organoid prediction vectors

Gene	Tiriac tumor median	Broad-94 median prediction	Sanger-148 median prediction
CDK6	-0.7534	-0.6432	-0.4754
STK11	-0.1422	-0.1779	-0.1541
CCND3	-0.2462	-0.2721	-0.2368
YAP1	-0.4810	-0.4049	-0.4243
MDM4	-0.0756	-0.1391	-0.1375
EP300	-0.2182	-0.2521	-0.2849
CTNNB1	-1.2821	-1.0060	-0.8711
EGFR	-0.4139	-0.3643	-0.3430
TSC2	-0.2079	-0.2829	-0.2684
TSC1	-0.0459	-0.1083	-0.1019
IGF1R	-0.2573	-0.3622	-0.4844
FGFR1	0.0486	-0.0060	0.0393
CCNE1	-0.2930	-0.1735	-0.2303
E2F3	-0.3342	-0.2347	-0.2920
ERBB3	-0.1929	-0.2145	-0.2050
NFE2L2	-0.2727	-0.2249	-0.1730
BRAF	-0.4354	-0.2175	-0.2272
KEAP1	-0.2889	-0.2719	-0.2779
MAPK1	-0.2095	-0.1818	-0.1882
RAF1	-0.2511	-0.1271	-0.1639

Note. *Concordance was calculated across the fixed 20-target panel. Because all compared values were predictions from the same locked model family and Tiriac lacked measured CRISPR outcomes, these correlations represent cross-cohort target-hierarchy consistency rather than independent predictive accuracy.*

Table S15. Cont.

(d) Expression-domain diagnostics and claim boundaries

Diagnostic or boundary	Result	Interpretation
Common frozen expression space	3,790 features	Features jointly available for Tiriac, Broad-94, and Sanger-148 domain diagnostics
PCA diagnostic	Tiriac samples separated distinctly from Broad-94 and Sanger-148	Descriptive multivariable evidence of cohort/domain shift
Tiriac vs Broad-94	3,779 finite SMD values; median |SMD| = 0.7434; |SMD| > 1 = 38.74%; |SMD| > 2 = 13.15%	Substantial feature-wise expression-domain shift
Tiriac vs Sanger-148	3,785 finite SMD values; median |SMD| = 0.8485; |SMD| > 1 = 43.41%; |SMD| > 2 = 14.61%	Substantial feature-wise expression-domain shift
Use of domain diagnostics	Reporting only	No recalibration, feature deletion, target removal, or model revision was allowed
Permitted claim language	Projected; applied; prioritized	Appropriate for a cohort without measured genome-scale CRISPR dependency outcomes
Disallowed claim language	Validated dependency; confirmed dependency; therapeutic sensitivity	Would overstate the evidence level of the Tiriac analysis
Principal limitation	No measured CRISPR Gene Effect matrix; normal group n = 11	Model accuracy could not be calculated and tumor-normal contrasts require caution

Note. *PCA, principal component analysis; SMD, standardized mean difference. Domain-shift diagnostics were descriptive and did not change the target panel, feature sets, hyperparameters, or model predictions. Tumor-normal q values describe differences in frozen model outputs and must not be interpreted as target validation.*

Table S16. Retrospective assessment of external feature-coverage thresholds.

This retrospective sensitivity analysis compared feature-coverage thresholds. The prespecified 80% input-eligibility rule was retained; 90% and 95% were evaluated as stricter alternatives using the archived feature-coverage counts.

External cohort	Available predictors per target	Observed coverage	Models passing 80%	Models passing 90%	Models passing 95%
Broad-94	300/300	100%	20/20	20/20	20/20
Sanger-148	291-300/300	97.0%-100%	20/20	20/20	20/20
Tiriac-55	292-300/300	97.33%-100%	20/20	20/20	20/20

Note. Coverage equals the number of frozen predictors present in the external expression matrix divided by 300, calculated separately for each target model. The 80%, 90%, and 95% requirements correspond to at least 240, 270, and 285 available predictors, respectively. These are target-model eligibility counts, not counts of organoids or patients.

The 80% requirement was a pragmatic safeguard against substantial feature absence, not an empirically optimized cutoff or a validated tolerance for 20% missingness. All observed model-specific coverages exceeded 95%, so the stricter requirements would retain the same models and leave frozen predictions and their reported summaries unchanged. This check did not modify feature availability, model coefficients, or missing-feature handling and should not be interpreted as an imputation sensitivity analysis.

Source: the archived new_3d_feature_coverage.csv files for Broad-94, Sanger-148, and Tiriac in the reproducibility package. The recorded available-feature counts were divided by 300 and compared with each threshold. The SHA-256 digest of each source coverage file matched its prediction-lock record.

Table S17. Retrospective evaluation of gene ranking within individual organoids.

This retrospective secondary analysis was defined after external outcomes were available. Unlike the primary target-specific Spearman correlation, which ranks organoids for a given gene, this endpoint ranks the fixed 20 genes within each organoid. The archived predictions and measured outcomes were used for all 94 Broad and 148 Sanger organoids; Sanger represented 144 donors. No model retraining, external recalibration or target selection was performed. Tiriac was excluded because measured dependency outcomes were unavailable.

Spearman correlation was calculated across the 20 genes within each organoid, then averaged across organoids. The fixed 2D mean reference used each gene's arithmetic mean measured dependency across all 852 deployment-training models and assigned the same vector to every organoid. The second comparator used archived lineage-only predictions. Neither comparator was fitted to external outcomes. Native Gene Effect and corrected/scaled LFC outcomes were evaluated separately by cohort; lower values indicated stronger dependency.

Method or paired contrast	Broad94: mean Spearman [95% CI]	Sanger148: mean Spearman [95% CI]
Expression model	0.3428 [0.3026, 0.3826]	0.4294 [0.3926, 0.4645]
Fixed 2D mean reference	0.2057 [0.1606, 0.2488]	0.1995 [0.1654, 0.2335]
Lineage-only reference	0.2745 [0.2370, 0.3119]	0.3884 [0.3493, 0.4254]
Expression minus fixed 2D mean	0.1371 [0.0987, 0.1780]	0.2299 [0.1981, 0.2613]
Expression minus lineage-only	0.0684 [0.0334, 0.1032]	0.0411 [0.0167, 0.0656]

Note. CI, confidence interval. Intervals are the 2.5th and 97.5th percentiles of 10,000 paired bootstrap resamples. The original Broad analysis sampled organoids within observed lineage strata because donor identifiers were absent from its evaluation records. Later partial-donor ranking sensitivities are reported in Table S24. Sanger donors were sampled as clusters, retaining all organoids of each sampled donor and an organoid-weighted mean. Seeds were 20260928 and 20260929, respectively. Tied values received average ranks; no undefined correlations or incomplete gene–organoid pairs occurred. The archived lineage-reference implementations differed by cohort, so results were not pooled.

Interpretation. The positive paired differences support ranking measured dependencies within this selected panel. These coefficients do not replace the original target-specific correlations or establish genome-wide prioritization, therapeutic efficacy or organoid-specific clinical benefit. Confidence intervals condition on the frozen panel and models; they do not capture uncertainty from gene selection or retraining. This retrospective endpoint is not a new blinded validation.

Table S18. Retrospective overlap of predicted and measured top-three gene sets.

For each organoid, the three lowest predicted scores and the three lowest measured scores defined the strongest predicted and observed dependencies. Top-three overlap was the number of genes common to these two sets divided by three. The table reports the mean overlap across organoids. Differences are expressed in percentage points, not relative percentage changes. This is a descriptive secondary endpoint, not overall classification accuracy.

Method or paired contrast	Broad94: value [95% CI]	Sanger148: value [95% CI]
Expression model (%)	34.04 [29.79, 37.94]	42.79 [39.46, 46.17]
Fixed 2D mean reference (%)	26.60 [22.34, 30.85]	8.56 [6.26, 11.11]
Lineage-only reference (%)	34.75 [30.50, 39.01]	35.36 [32.44, 38.32]
Expression minus fixed 2D mean (percentage points)	7.45 [1.77, 12.77]	34.23 [30.68, 37.61]
Expression minus lineage-only (percentage points)	-0.71 [-4.96, 3.19]	7.43 [4.28, 10.74]

Note. Confidence intervals used the same 10,000 paired resamples and sampling units as Table S17. Exact ties at the top-three boundary were assigned deterministically by ascending frozen target identifier; no boundary ties occurred in measured outcomes, expression predictions or either reference. No multiple-comparison adjustment was applied to these secondary contrasts. All 20 panel genes and all organoids were retained.

Interpretation. The expression model recovered an average of 1.02 of the three measured strongest dependencies in Broad and 1.28 in Sanger. In Sanger, overlap exceeded the lineage-only reference by 7.43 percentage points (95% CI 4.28–10.74). In Broad, the difference was −0.71 points (−4.96 to 3.19); superiority over the lineage-only reference for selecting the first three genes was not demonstrated. The full-ranking and top-three findings should therefore be reported together.

Table S19. Sanger sensitivity analysis using one averaged profile per donor.

For each of the 144 Sanger donors, the 20-gene expression-prediction, observed-outcome and lineage-reference vectors were averaged across that donor's organoids before ranks were calculated. The fixed 2D mean reference was unchanged. Spearman correlation and top-three overlap were recomputed for each donor profile and averaged with equal donor weights. Percentile 95% confidence intervals used 10,000 paired donor bootstrap resamples (seed 20260930). This differs from the primary Sanger148 analysis, which retained individual organoids and used donor-cluster resampling.

Method or paired contrast	Within-profile Spearman [95% CI]	Top-three overlap or difference [95% CI]
Expression model	0.4338 [0.3986, 0.4675]	42.82 [39.35, 46.06] %
Fixed 2D mean reference	0.2041 [0.1700, 0.2379]	8.56 [6.25, 10.88] %
Lineage-only reference	0.3937 [0.3562, 0.4298]	35.88 [33.10, 38.66] %
Expression minus fixed 2D mean	0.2298 [0.1989, 0.2601]	34.26 [30.79, 37.73] pp
Expression minus lineage-only	0.0402 [0.0157, 0.0652]	6.94 [4.17, 9.95] pp

Note. pp, percentage points. All 144 donor profiles retained all 20 genes. No undefined correlations or top-three boundary ties occurred. Model and reference rows report overlap percentages; contrast rows report percentage-point differences. The sensitivity analysis preserved the direction and approximate magnitude of the Sanger148 full-ranking and top-three comparisons.

Reproducibility. Tables S17–S19 derive from 07_within_organoid_ranking_revision/results/summary_metrics.csv in the accompanying reproducibility package. The parent analysis directory, 07_within_organoid_ranking_revision/, contains run_analysis.py, the protocol and its SHA-256 digest, and the input and result subdirectories, including per-profile results, bootstrap replicates and validation_checks.json. Source hashes and frozen prediction locks were checked, the original target-wise metrics were reproduced, all within-profile correlations were independently checked with scipy.stats.spearmanr, and the first 25 bootstrap replicates were checked by direct row expansion.

Table S20. Retrospective sensitivity of Tiriac projections and gene rankings to missing-feature imputation.

Training-median imputation was the reference method for this retrospective sensitivity analysis. The archived code, input and model hashes were verified, and all 55 × 20 frozen predictions were reproduced exactly. The same frozen models were evaluated with zero filling on the log2(TPM + 1) scale before standardization and with stored training-reference means. These means were estimated after median completion of the 852-model training data. Only the 39 absent unique features were changed; measured zeros and all other observed values were retained. No model fitting or external recalibration was performed.

Comparison with the original training-median reference	Zero filling	Training-reference mean
Mean absolute change across 1,100 sample–target predictions	0.011402	0.001705
Maximum absolute change across 1,100 predictions	0.107673	0.017193
Mean within-profile Spearman vs reference: all 49 cases	0.9734	0.9993
Identical top-three sets: all 49 cases	46/49	49/49
Mean within-profile Spearman vs reference: 38 tumor cases	0.9771	0.9991
Mean top-three set overlap: 38 tumor cases (%)	98.25	100.00
Identical top-three sets: 38 tumor cases	36/38	38/38
Identical top-three sets: 11 normal cases	10/11	11/11
Identical top-three sets: sample-level sensitivity	51/55	55/55
Spearman agreement of the 20-target tumor-median hierarchy	0.9850	1.0000

Note. Prediction changes are in projected Gene Effect units. Case profiles average all organoid samples from the same case before ranking; there were 38 tumor and 11 normal cases. Lower scores define the stronger projected dependencies. Top-three overlap is set intersection divided by three, averaged across profiles; it is not accuracy against measured dependencies. Ties use average ranks and ascending target keys for selection; no top-three boundary ties occurred. No uncertainty interval is assigned to these deterministic method comparisons. The original first-ranked target was retained in all 49 cases under both alternatives.

Interpretation. Zero filling changed the top-three set in two tumor cases and one normal case. Training-reference means preserved all case-level top-three sets. The tumor-median top-three hierarchy remained CTNNB1, CDK6 and YAP1. Spearman concordance of the tumor-median hierarchy with Broad/Sanger predictions was 0.6692/0.7383 under zero filling and 0.7173/0.7805 under training-reference means (original: 0.7173/0.7805). These findings assess stability of projections; Tiriac has no measured dependency outcomes.

Table S21. Target-specific missing features and projection shifts in Tiriac.

For each frozen target model, 300 expression predictors were retained. The absence pattern was shared by all 55 samples. Signed shifts below are alternative minus training-median prediction, calculated independently from the stored coefficients, feature scales and replacement values. A negative shift denotes a more negative projected dependency score. The original projections were not replaced by either sensitivity scenario.

Target	Missing predictors / 300	Missing predictors with nonzero coefficient	Zero minus median shift	Training-reference mean minus median shift
CDK6	2	2	−0.030420	−0.002076
STK11	3	1	−0.008580	−0.000785
CCND3	3	1	0.000000	−0.001385
YAP1	3	0	0.000000	0.000000
MDM4	3	0	0.000000	0.000000
EP300	3	1	−0.005906	−0.000090
CTNNB1	8	4	+0.002064	−0.017193
EGFR	0	0	0.000000	0.000000
TSC2	2	0	0.000000	0.000000
TSC1	2	0	0.000000	0.000000
IGF1R	2	1	−0.022442	−0.000049
FGFR1	2	0	0.000000	0.000000
CCNE1	4	0	0.000000	0.000000
E2F3	2	2	−0.037562	−0.003538
ERBB3	1	0	0.000000	0.000000
NFE2L2	4	2	−0.013385	−0.007190
BRAF	2	1	0.000000	−0.001349
KEAP1	1	0	0.000000	0.000000
MAPK1	3	0	0.000000	0.000000
RAF1	3	1	−0.107673	−0.000448

Note. Missing predictors with a zero Elastic Net coefficient cannot alter the projection. A nonzero coefficient can still give a zero zero-fill shift when its training median is zero or contributions cancel. For each target, the shift equals the sum over missing features of coefficient × (alternative replacement − training median) / training scale. Direct model predictions agreed with this constant-offset calculation to within 0.000002; the maximum error of an independent full coefficient replay was below 0.000001.

The original tumor–normal comparison was repeated with 5,000 case bootstrap resamples (seed 20260830), two-sided Mann–Whitney tests and Benjamini–Hochberg adjustment across 20 targets. Median differences and bootstrap limits changed by less than 0.000001; p-values, q-values and all 15 significant classifications were unchanged. Constant target-specific offsets preserve within-target sample ranks and tumor–normal differences algebraically; their invariance is not independent evidence of robustness or accuracy.

Reproducibility. The accompanying package directory 08_tiriac_imputation_sensitivity_revision/ contains run_analysis.py, the retrospective protocol, source hashes, all sample and case projections, per-profile ranking comparisons, missing-feature contributions, tumor–normal results and validation checks. This retrospective sensitivity analysis was not prespecified in the study protocol. It cannot determine which filling method is most accurate without measured Tiriac dependency outcomes.

Table S22. Broad cohort-size reconciliation and donor metadata coverage.

Audit item	Observed count or finding	Interpretation
Archived Broad 3DO records	95 unique models	One screen record per ModelID.
QC exclusion	ACH-003044; PassesQC = False	94 passing ModelIDs exactly matched the frozen analytical registry.
Analysis-ready matrices	94 × 20 predictions and outcomes	1,880 unique model–target pairs; original macro ρ = 0.1518231406 reproduced.
Supplied DepMap Model.csv	2,154 source models; 16 Broad matches; 15 PatientID groups	Known pair: ACH-002883 and ACH-002884; PatientID PT-N65Hvq.
Archived Sanger exclusion registry	9 additional Broad models; 9 source-specific donor IDs	No ModelID overlap with the 16 DepMap-mapped records.
Combined donor-documented subset	25 models; 24 source-specific groups	Cross-source donor equivalence remains unresolved.
Unresolved donor metadata	69 of 94 models	Absence of a known linkage does not establish independence.
Conditional full-cohort resampling	93 groups	Known pair clustered; 69 unresolved models treated as singleton units by assumption.

The supplied metadata were matched exactly by ModelID. Donor identifiers were namespaced by source; no links were inferred from lineage or model-name similarity. The complete 94-row mapping, source hashes and QC-excluded record are provided in the application package. These counts distinguish model availability from donor ascertainment.

Table S23. Retrospective Broad target-wise donor sensitivity analyses.

Scenario	Profiles / units	Expression macro ρ (95% CI)	Lineage macro ρ (95% CI)	Paired difference (95% CI)
Full 94, model replay	94 / 94	0.1518 (0.0993 to 0.2016)	0.0951 (0.0469 to 0.1424)	0.0567 (0.0005 to 0.1120)
Full 94, partial clustering	94 / 93	0.1518 (0.1009 to 0.2007)	0.0951 (0.0473 to 0.1415)	0.0567 (0.0027 to 0.1081)
Omit ACH-002883	93 / 93	0.1516 (0.1006 to 0.2008)	0.0947 (0.0459 to 0.1418)	0.0569 (0.0025 to 0.1092)
Omit ACH-002884	93 / 93	0.1539 (0.1026 to 0.2028)	0.0963 (0.0479 to 0.1430)	0.0576 (0.0028 to 0.1099)
Known 25, model replay	25 / 25	0.0741 (-0.0150 to 0.1647)	0.1313 (0.0468 to 0.2131)	-0.0571 (-0.1465 to 0.0300)
Known 25, donor clustering	25 / 24	0.0741 (-0.0154 to 0.1618)	0.1313 (0.0484 to 0.2147)	-0.0571 (-0.1502 to 0.0305)
Known subset, donor means	24 / 24	0.0727 (-0.0195 to 0.1629)	0.1331 (0.0449 to 0.2218)	-0.0604 (-0.1599 to 0.0334)

All values use the same frozen 20-target panel. Confidence intervals are percentiles from 5,000 paired bootstrap resamples within observed lineage strata (seed 20260929). Complete source-specific donor groups were retained in cluster resampling. The two model-level replays are matched references with the new seed, not replacements for the original reported intervals. The full-cohort partial-cluster scenario assumes singleton units for all 69 unresolved models. Omission scenarios remove one known replicate but cannot exclude other donor links. The donor-mean scenario averages prediction, outcome and reference vectors before reranking, uses equal group weights, and changes the estimand. Other estimates retain organoid weighting. Undefined target correlations contribute zero as in the archived implementation. Comparisons are retrospective and unadjusted for multiple sensitivity analyses.

The 25-model subset has different model and lineage composition from Broad-94 and is not a representative replacement cohort. Its 24 source-specific groups are not proof of 24 unique biological donors across both data sources. Neither 93 conditional resampling groups nor 24 source-specific groups is an estimate of the verified effective sample size of Broad-94.

Table S24. Retrospective Broad within-organoid ranking sensitivities.

Scenario	Mean within-profile ρ (95% CI)	ρ difference vs lineage (95% CI)	Top-three overlap % (95% CI)	Difference vs lineage pp (95% CI)
Full 94, model replay	0.3428 (0.3033 to 0.3813)	0.0684 (0.0332 to 0.1052)	34.04 (29.79 to 38.30)	-0.71 (-4.96 to 3.19)
Full 94, partial clustering	0.3428 (0.3047 to 0.3817)	0.0684 (0.0324 to 0.1030)	34.04 (30.11 to 37.99)	-0.71 (-4.86 to 3.19)
Omit ACH-002883	0.3392 (0.3007 to 0.3782)	0.0663 (0.0306 to 0.1005)	33.69 (29.75 to 37.99)	-1.08 (-5.02 to 2.87)
Omit ACH-002884	0.3450 (0.3060 to 0.3845)	0.0666 (0.0308 to 0.1006)	34.41 (30.11 to 38.35)	-0.72 (-5.02 to 3.23)
Known 25, model replay	0.3305 (0.2557 to 0.4101)	-0.0097 (-0.0728 to 0.0502)	33.33 (25.33 to 41.33)	-2.67 (-10.67 to 5.33)
Known 25, donor clustering	0.3305 (0.2558 to 0.4017)	-0.0097 (-0.0775 to 0.0546)	33.33 (26.39 to 40.28)	-2.67 (-11.11 to 5.13)
Known subset, donor means	0.3291 (0.2543 to 0.4050)	-0.0225 (-0.0814 to 0.0343)	33.33 (26.39 to 40.28)	-2.78 (-11.11 to 5.56)

Scenarios, grouping assumptions, weighting and bootstrap settings follow Table S23. Within-profile Spearman correlation ranks the 20 genes within each profile and is averaged across profiles. Top-three overlap is the fraction of the three most negative measured scores recovered among the three most negative predicted scores. Differences use the frozen lineage-only reference and paired resamples. No top-three boundary ties occurred. The donor-mean row ranks the averaged 20-gene vectors; other rows average individual organoid endpoints. pp, percentage points. These secondary comparisons are retrospective, restricted to the selected panel and not adjusted for multiplicity. They do not resolve the remaining donor uncertainty or establish prospective experimental benefit.
